# RNA profiling of laser microdissected human trophoblast subtypes at mid-gestation reveals a role for cannabinoid signaling in invasion

**DOI:** 10.1242/dev.199626

**Published:** 2021-10-19

**Authors:** Matthew Gormley, Oliver Oliverio, Mirhan Kapidzic, Katherine Ona, Steven Hall, Susan J. Fisher

**Affiliations:** 1 Department of Obstetrics, Gynecology, and Reproductive Sciences, Center for Reproductive Sciences, University of California, San Francisco, CA 94143, USA; 2 Eli and Edythe Broad Center for Regeneration Medicine and Stem Cell Research, University of California, San Francisco, CA 94143, USA; 3 Department of Anatomy, University of California, San Francisco, CA 94143, USA

**Keywords:** Human, Placenta, Trophoblast, Transcriptomics, Proteomics, Cannabinoid signaling

## Abstract

Human placental architecture is complex. Its surface epithelium, specialized for transport, forms by fusion of cytotrophoblast progenitors into multinucleated syncytiotrophoblasts. Near the uterine surface, these progenitors assume a different fate, becoming cancer-like cells that invade its lining and blood vessels. The latter process physically connects the placenta to the mother and shunts uterine blood to the syncytiotrophoblasts. Isolation of trophoblast subtypes is technically challenging. Upon removal, syncytiotrophoblasts disintegrate and invasive cytotrophoblasts are admixed with uterine cells. We used laser capture to circumvent these obstacles. This enabled isolation of syncytiotrophoblasts and two subpopulations of invasive cytotrophoblasts from cell columns and the endovascular compartment of spiral arteries. Transcriptional profiling revealed numerous genes, the placental or trophoblast expression of which was not known, including neurotensin and *C4ORF36*. Using mass spectrometry, discovery of differentially expressed mRNAs was extended to the protein level. We also found that invasive cytotrophoblasts expressed cannabinoid receptor 1. Unexpectedly, screening agonists and antagonists showed that signals from this receptor promote invasion. Together, these results revealed previously unseen gene expression patterns that translate to the protein level. Our data also suggested that endogenous and exogenous cannabinoids can affect human placental development.

## Abstract (precis)

**Summary:** Transcriptomic and proteomic profiling of laser captured human trophoblasts showed that placental cells lining uterine arteries express cannabinoid receptor 1. Functional analyses suggest that endogenous/exogenous cannabinoids could affect placentation.

## INTRODUCTION

At birth, the human placenta is the newborn's largest organ (Pryce et al., 2014), its weight being approximately double that of the heaviest organ of the body, the brain (Boyd and Hamilton, 1967). During the last six months of pregnancy, placental weight increases over sevenfold (Boyd and Hamilton, 1967) while simultaneously supporting over a thirtyfold increase in that of the fetus (Armitage et al., 1967).

The placenta's ability to transport the very substantial resources that are needed for its own growth and that of the embryo/fetus is enabled by its unique structure. Its surface area is increased by many orders of macroscopically visible branches – the chorionic villi. At term, the total area in direct contact with maternal blood is estimated to be ∼12 m^2^ (Boyd, 1984). At an electron microscopic level, the syncytiotrophoblasts (STBs) that form the outer surface of the villi are covered with branched microvilli, which substantially add to the surface area, by our estimate producing at least a tenfold increase (Wislocki and Dempsey, 1955). Furthermore, the human placenta is hemochorial, i.e., in direct contact with uterine blood, minimizing the cellular barriers between the maternal source of nutrients and oxygen, and the placental vasculature that carries these substances to the embryo/fetus.

**Fig. 1.**
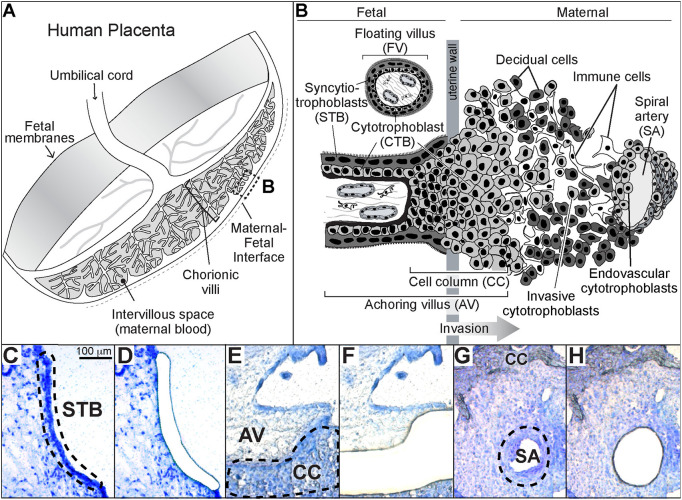
**Human placenta trophoblast subtypes targeted for laser microdissection.** (A) Diagram of the human placenta in the second trimester of pregnancy. The boxed area (B) indicates the region biopsied for these studies. (B) View of the maternal-fetal interface at the cellular level. Shown are the two classes of chorionic villi. Floating villi (FV), which are covered by multinucleated syncytiotrophoblasts (STB), transport substances between the mother and the embryo/fetus while producing growth factors and hormones essential to the unique symbiosis of pregnancy. Anchoring villi (AV), contain mononuclear cytotrophoblast progenitors that either fuse to form STBs or exit the placenta proper through cell columns (CCs) that attach the embryo/fetus to the uterus. Endovascular cytotrophoblasts mediate an increase in the terminal diameter of the maternal spiral arteries, enabling increased blood flow to the placenta. The arrow indicates the direction of trophoblast invasion (modified from Damsky et al., 1994). (C-G) Photomicrographs taken before and after STB, CTB and ENDO were isolated using LMD. The sections were stained with Toluidine Blue, which enabled visualization of the cellular architecture. The dotted lines indicate the three trophoblast-containing regions that were targeted. SA, spiral artery.

At a cellular level, human placental structure is established by differentiation of its progenitor population, villous cytotrophoblasts (CTBs; Fig. 1A,B; reviewed by Maltepe and Fisher, 2015). In one pathway, the cells fuse to produce multinucleated STBs. In the other pathway, the cells leave the placenta, forming bridges (termed cell columns) that connect to the uterus and are the conduit for CTBs that invade its wall. The process is accompanied by a dramatic phenotypic switch in which the formerly epithelial cells adopt many vascular properties (Damsky and Fisher, 1998). Remarkably, these cells also breach uterine vessels that lie in their path. They penetrate the end of veins and migrate in a retrograde fashion up spiral arteries, the walls of which they occupy throughout much of their intrauterine segments. The latter process establishes the funnel-like structure of spiral arteries that reduces the flow rate and increases the volume of maternal blood perfusing the placenta.

The cellular structure of the human placenta makes isolation of the various trophoblast subtypes difficult to impossible. Upon enzyme dissociation, the syncytium disintegrates and extravillous CTBs are only a small fraction of the mononuclear cells that can be isolated. To solve this problem, we have used laser microdissection (LMD) to capture STBs and column, endovascular or smooth CTBs as well as decidual cells for RNA profiling in the context of severe preeclampsia (Garrido-Gomez et al., 2017; Gormley et al., 2017). Here, we used this approach to study gene expression during the 2nd trimester of normal pregnancy. The results revealed numerous molecules that were not previously known to be expressed by STBs and/or CTBs. Among them was the cannabinoid receptor 1 (CNR1; also known as CB1), which was highly expressed by the endovascular CTB subpopulation. We went on to show that the downstream signals can influence invasion.

## RESULTS

### RNA profiling of human trophoblast subtypes at mid-gestation

Blocks were prepared from biopsies of the 2nd trimester (15-20 weeks gestation) maternal-fetal interface. The focus was on areas that by macroscopic inspection included spiral arteries with dilated termini, a sign of CTB invasion. LMD enabled the capture of syncytium, cell columns and the endovascular compartment. The experimental strategy, which we used previously in an analysis of the same cell types from severe preeclampsia (Gormley et al., 2017), is depicted in Fig. 1. The dashed lines drawn on the photomicrographs to the left show the trophoblast subpopulations that were targeted for removal as shown to the right: STBs (Fig. 1C,D), cell column CTBs (CC; Fig. 1E,F), and the endovascular compartment (ENDO), containing CTBs and other cell types in spiral arteries (SA; Fig. 1G,H).

Global profiling of RNA isolated from the samples and pair-wise comparisons of the results revealed 2986 genes that were differentially expressed (DE; *adjP*-value <0.05) by one of the trophoblast subtypes. Principal component analysis (PCA; Fig. S1) showed that data from the same cell type clustered together separate from the other samples. Thus, consistent with their different functions, the various trophoblast subtypes analyzed had distinct transcriptomes.

**Fig. 2.**
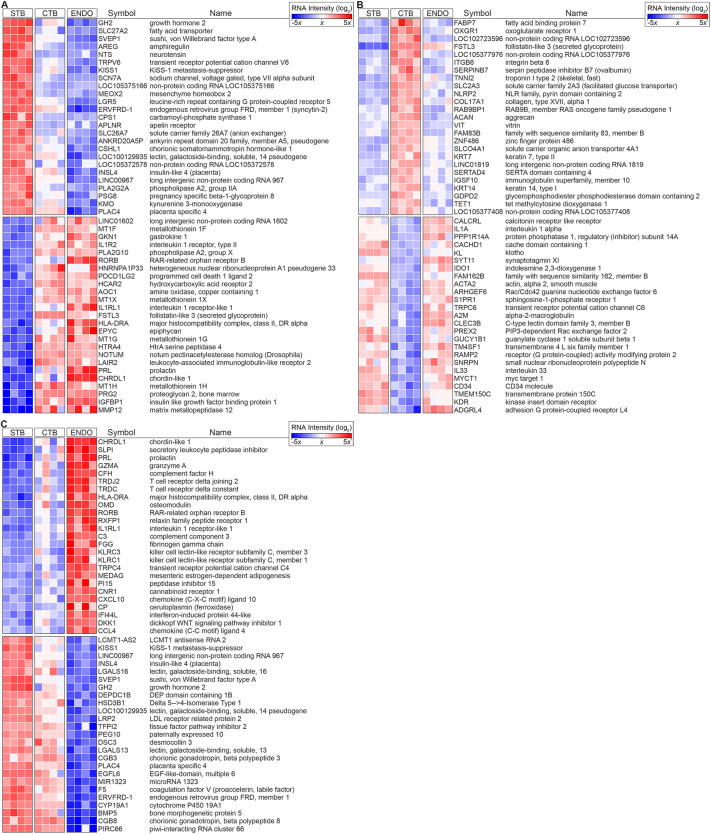
**Heatmaps of differentially expressed STB, CTB and ENDO RNAs.** (A) The 25 most highly upregulated and 25 most highly downregulated transcripts in syncytiotrophoblasts (STB) compared with cytotrophoblasts (CTB) and endovascular cytotrophoblasts (ENDO) (total=2708, Table S2). (B) Heatmap of the most highly differentially expressed transcripts in CTB (total=2070, Table S2) relative to STB and ENDO. (C) Heatmap of the most highly differentially expressed transcripts in ENDO (total=2753, Table S2) relative to STB and CTB. The data are displayed as a heatmap showing the relative RNA fold change as a variation in color from blue (decreased expression) to white (average expression) to red (increased expression). Cells captured from four placentas were analyzed.

Compared with the two CTB subpopulations, 2708 genes were differentially expressed in STBs (Fig. 2A; see Fig. S2 for entire heatmap and fold changes). As expected, the upregulated category included genes encoding growth-promoting molecules (e.g. *GH2*, *AREG*, *CSHL1*, *INSL4*), numerous transporters (e.g. *SLC27A2*, *SLC26A7*), ion channels (e.g. *TRPV6*, *SCN7A*) and syncytin 2 (*ERVFRD-1*)*.* The transcript for *MEOX2* was also differentially expressed in STBs, suggesting the possibility that this homeobox gene plays a role in fate specification or maintenance of this cell type. Unexpectedly, the expression of *LGR5*, which encodes a cell surface molecule that marks stem cells (e.g. intestinal and colonic; Leung et al., 2018), was unique to STBs. Of interest was detection of the mRNA encoding neurotensin (NTS), raising the possibility that this small neuropeptide is released from the surface of the placenta into maternal blood during pregnancy, following which it seems likely that enteric and other effects are possible (Bugni and Pothoulakis, 2013).

With regard to cell column CTBs, 2070 genes were differentially expressed in this subpopulation (Fig. 2B; see Fig. S2 for entire heatmap and fold changes). Receptors for metabolites were the most highly upregulated. They included *FABP7a*. We have previously described CTB expression of this molecule at the maternal-fetal interface (Winn et al., 2007). The alpha-ketoglutarate receptor (*OXGR1*) was highly expressed. This result suggested that CTBs might be using metabolic products in the basal plate as an energy source, which could couple the initial stages of invasion to maternal metabolism. As shown by our previous work (reviewed by Maltepe and Fisher, 2015), molecules that are involved in adhesion/de-adhesion were upregulated in columns (*ITGB6*, *COL17A1*, *ACAN*, *VIT*). An mRNA encoding troponin I (*TNNI2*), a protein involved in muscle contraction, was DE, possible evidence of its involvement in movement of these cells through the columns and into the uterus. In accord with the dramatic phenotypic changes the cells undergo, the DNA demethylase *TET1* was upregulated, suggesting that alterations at the level of the epigenome might play a role in specifying the fate of this CTB population.

Overall, 2753 genes were differentially expressed in the endovascular compartment (Fig. 2C; see Fig. S2 for entire heatmap and fold changes). The most highly upregulated genes included the BMP4 antagonist *CHRDL1*, which mitigates migration and invasion of breast cancer cells (Cyr-Depauw et al., 2016), the secretory leukocyte peptidase inhibitor *SLPI*, which plays a role in host defense (Majchrzak-Gorecka et al., 2016) and a calcium channel involved in cell signaling (*TRPC4*), which promotes vessel relaxation and permeability (Freichel et al., 2004). We detected expression of molecules that could control leakage of blood from modified spiral arteries (*FGG*, *CP*) and the complement cascade (*CFH*, *C3*). To the best of our knowledge, this list included previously unseen identifications: *OMD*, *RORB* and *MEDAG*. *CNR1* was also in this category, which raised the possibility that endogenous and exogenous cannabinoids could impact CTB invasion and remodeling of the uterine vasculature.

Finally, we detected expression of mRNAs that were indicative of contamination with other cell types, not unexpected because endovascular CTBs lie adjacent to the decidua, which expresses relaxin (RLN1) (Goldsmith and Weiss, 2009) and *PRL* (Jones et al., 1998), which we detected. NK cells infiltrate the walls of spiral arteries (Moffett and Colucci, 2015), which we found evidence for in the expression of molecules these immune cells produce, including *GZMA*, *KLRC1* and *KLRC3*. We also detected molecules expressed by T cells (*TRDJ2*, *TRDC*).

In parallel, we have been developing a mass spectrometry (MS)-based proteomic method for analyzing the same trophoblast subpopulations, captured by LMD, which were the focus of the transcriptomic analysis. Given that typical estimates of correlations between mRNA and protein expression are below 50% (Maier et al., 2009; Vogel and Marcotte, 2012), we were interested in those that were significant at both levels, making them more likely to have functional effects. For these experiments, we employed a shotgun proteomic approach in which the number of times a peptide is identified enables relative quantification of the parent protein (i.e. spectral counting; Zhang et al., 2013). In all, we showed that 108 differentially expressed mRNAs had similar relative protein abundances within STB, CTB or ENDO (Fig. S3).

**Fig. 3.**
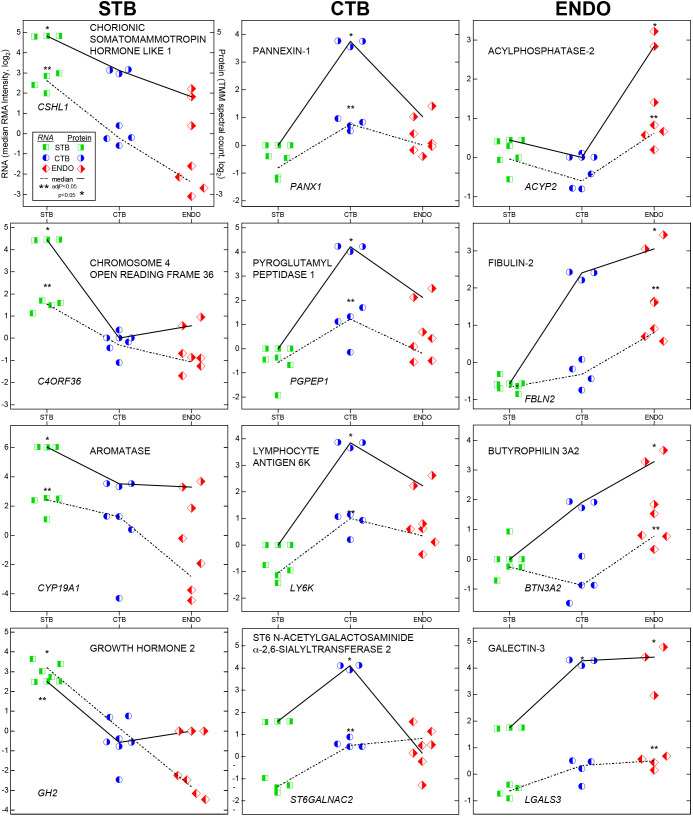
**Plots of relative RNA and protein abundances in trophoblast subtypes.** The upper left hand panel illustrates the labeling scheme. Data are organized in columns. Molecules that were upregulated in STBs are shown to the left, cell column CTBs in the middle and endovascular CTBs to the right. Each cell type is designated by a different bisected symbol (square, circle or diamond). Left or right shading denotes RNA or protein values, respectively, which is colored (green, blue or red) to aid interpretation. RNA data were from four biological replicates, protein data were from three biological replicates.

Examples of these data are shown in Fig. 3. The upper left hand panel illustrates the labeling scheme, which was subsequently omitted for the sake of simplification. Data are organized in columns. Molecules that were upregulated in STB are shown to the left, CTB in the middle and ENDO to the right. Each cell type is designated by a different bisected symbol (square, circle or diamond). Left or right shading denotes RNA or protein values, respectively, which is colored (green, blue or red) to aid interpretation.

With regard to STBs (Fig. 3, left column), CSHL1 was the most highly expressed differentially expressed molecule at both mRNA and protein levels. We validated translation of an open reading frame (chromosome 4 open reading frame 36; *C4ORF36*). High placental levels of this mRNA (ENSG00000163633) have been reported (Fagerberg et al., 2014). Its fungal protein analog (MGG_01005) is dynein light chain Tctex-type 1 (dynlt1/3; Li et al., 2018), which transports various types of cellular cargo. *CYP19A1* (aromatase) and *GH2* (growth hormone 2) were also expressed at higher levels in the syncytium.

With regards cell column CTBs (Fig. 3, middle column), the highly-expressed differentially expressed molecules included several identifications that, to our knowledge, were not known to be produced by the placenta. They included pannexin 1 (a major ATP release and nucleotide eflux channel; Chekeni et al., 2010; Elliott et al., 2009), pyroglutamyl-peptidase I (*PGPEP1*; an exopeptidase; Cummins and O'Connor, 1998) and lymphocyte antigen 6K (*LY6K*; a biomarker of lung and esophageal carcinomas; Ishikawa et al., 2007). We were also interested to find that a sialyltransferase (alpha-n-acetylgalactosaminide alpha-2,6-sialyltransferase 6; Samyn-Petit et al., 2000) was upregulated in this CTB subpopulation. Its identification may be related to the observation that placental proteins carry unusual forms of glycosylation not found on the same molecules produced by other cell types (McMaster et al., 1998).

With regard to the endovascular compartment (Fig. 3, right column), the highly-expressed differentially expressed molecules included the hydrolase acylphosphatase 2, and fibulin 2, a secreted extracellular matrix glycoprotein that can stabilize the basement membranes of epithelial cells (Ibrahim et al., 2018). We also confirmed the differential expression of immune molecules: butyrophilin 3A2, which can inhibit T-cell activity, as do other members of this family (Messal et al., 2011; Rhodes et al., 2016) and galectin 3, which has numerous other functions, including a role in cell adhesion (Pugliese et al., 2015) and epithelial-stromal signaling (AbuSamra et al., 2019).

**Fig. 4.**
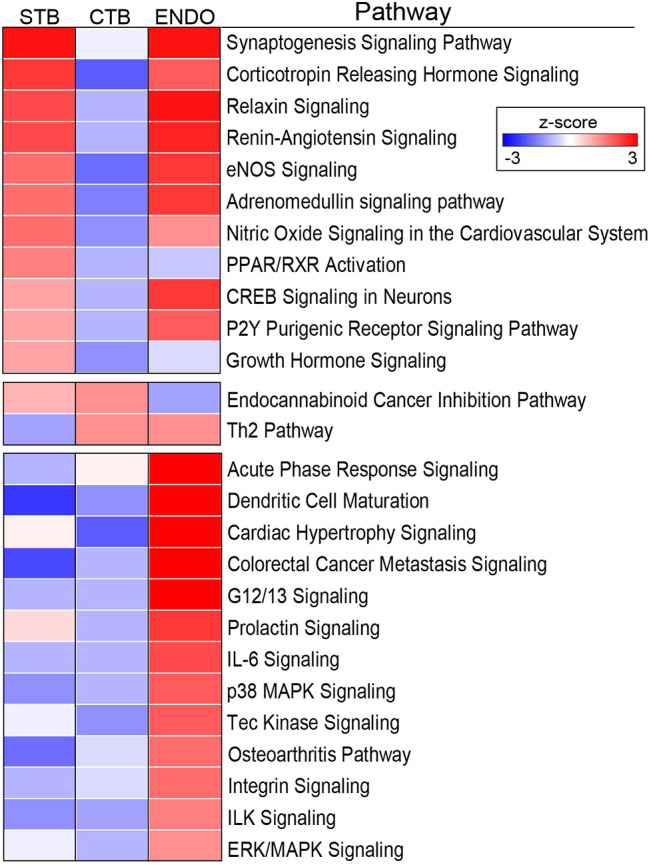
**Ingenuity Pathway Analysis of the differentially expressed genes among trophoblast subpopulations.** Pathways were assigned a *z*-score based on the predicted impact of DEGs, either activation (+) or deactivation (−). Significant pathways had a −log(*P*-value)>1.3, an absolute *z*-score>2, and contained at least four members in a unique combination. The data are displayed as a heatmap showing the *z*-score as a variation in color from blue (decreased expression) to red (increased expression) for each trophoblast subtype. The results were notable for the number signaling pathways that were identified.

Next, we used Ingenuity Pathways Analysis (IPA) to infer functional similarities and differences among the trophoblast subtypes. Pathways were assigned a *z*-score based on the predicted impact of differentially expressed genes (DEGs), either activation (+) or deactivation (−). Those shown in Fig. 4 were significantly over-represented (either positively or negatively) in all of the trophoblast subpopulations. Again, there was significant overlap between the *in silico* predictions of the pathways that were activated in STB and ENDO versus column CTBs. It seems possible that this convergence could be driven, in part, by the fact that these two regions are in direct contact with maternal blood. In keeping with this theory, many of the pathways identified as potentially activated were involved in responding to signals from circulating molecules: corticotropin releasing hormone, relaxin, renin, angiotensin, NO, adrenomedullin, fatty acids (via PPAR, RXR), purines and pyrimidines (via P2Y receptors) or growth hormone. We also found potential positive regulation of pathways that are involved in neuronal functions, including synaptogenesis and CREB signaling. The STB and column CTB data suggested that these cells may be able to respond to cannabinoids; cell columns and endovascular compartments upregulated the expression of genes that are involved in TH2 (tolerogenic) responses. There were a large number of unique pathways that were predicted to be activated solely by the endovascular trophoblasts and other cells types of the remodeled spiral arteries (ENDO). The putative pathways were involved in immune functions (acute phase signaling, dendritic cell maturation, IL6, Tec kinase signaling or osteoarthritis), cell movement (colorectal cancer metastasis as well as G12/13, integrin and ILK signaling), MAPK signaling, vascular biology (cardiac hypertrophy) and prolactin responses.

**Fig. 5.**
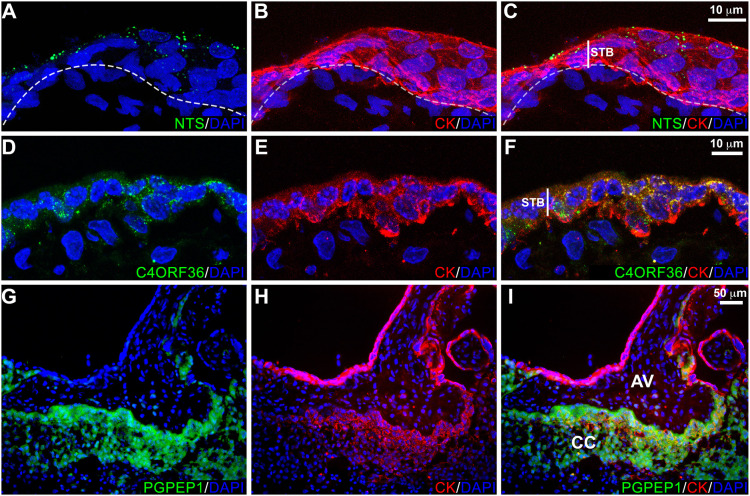
**Immunolocalization of differentially expressed proteins: NTS, C4ORF36 and PGPEP1.** Tissue sections were immunostained for the antigen of interest and cytokeratin (CK), a trophoblast marker. Nuclei were localized with DAPI. Each panel was a confocal *z*-stack maximum intensity projection. (A-C) Neurotensin (NTS) was detected in a vesicular pattern localized to the apical region of syncytiotrophoblasts (STB). Within any one tissue section the vesicle density varied among regions, although no areas lacked immunoreactivity. Furthermore, no differences based on gestational age were observed. (D-F) C4ORF36, which localized to STBs, had a uniformly dense punctate pattern. No variation was observed across the second trimester. (G-I) PGPEP1 was highly upregulated in cytotrophoblast columns (CC) of anchoring villi (AV). The results shown were represented of the immunostaining observed in a minimum of three biological replicates.

Given that the gene expression data were specific to various trophoblast subtypes, we were interested in showing that the differentially expressed molecules had the expected immunolocalization patterns. With regard to STBs, we chose neurotensin, a small peptide gut hormone with brain and nervous system effects including analgesia (Cordomi et al., 2016; Pérez de Vega et al., 2018; Tschumi and Beckstead, 2019), which to our knowledge was not known to be produced by the human placenta. The immunolocalization signal for this molecule (Fig. 5A-C) was detected as a vesicular pattern in the syncytium at higher density in the apical region of the cells, suggesting possible release into the maternal circulation. In addition, we were interested in the expression of C4orf36, also in STBs, the peptide product of which was detected by mass spectrometry. Immunostaining revealed a dense punctate pattern in STBs (Fig. 5D-F). Within the cell columns, we confirmed the expression of *PGPEP1* by the cytotrophoblasts in that region (Fig. 5G-I).

**Fig. 6.**
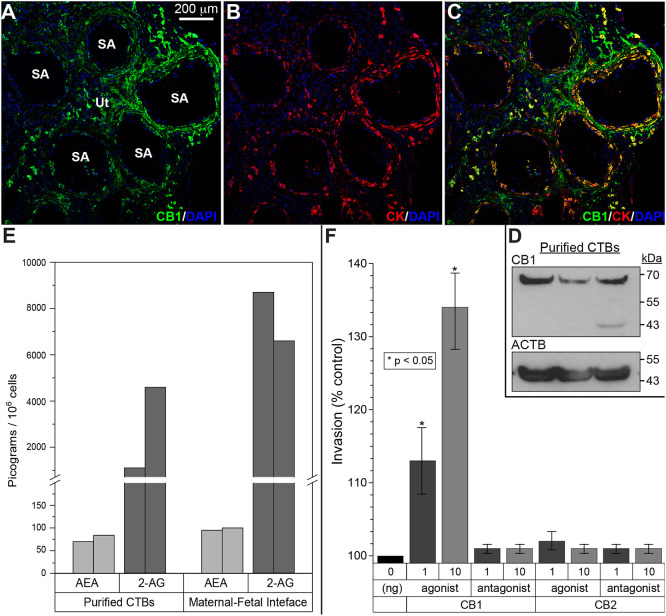
**Expression of cannabinoid ligands and receptors at the maternal-fetal interface and the functional consequences.** (A-C) Tissue sections were immunostained for CNR1 (CB1) and cytokeratin (CK), a trophoblast marker. Nuclei were localized with DAPI. Each panel was a confocal *z*-stack maximum intensity projection. Anti-CNR1 specifically reacted with invasive/endovascular CTBs in the walls of uterine (Ut) spiral arteries (SA). Cytotrophoblasts (CTBs) in the cell columns showed variable CNR1 expression, while syncytiotrophoblasts were negative (data not shown). The results shown were representative of the immunostaining observed in a minimum of three biological replicates. (D) Immunoblotting with the same antibody detected a band of the expected molecular weight in CTB lysates. Protein loading was estimated by immunoblotting with anti-actin-β (ACTB). Cells from three placentas were analyzed. (E) Mass spectrometry enabled measuring the absolute amounts of the major mammalian endogenous cannabinoids, anandamide (AEA) and 2-arachidonoylglycerol (2-AG), in isolated CTBs and biopsies of the maternal-fetal interface that included invasive CTBs. The results from replicate samples are shown. (F) Among the CNR1 and CNR2 agonists and antagonists assayed, only a CNR1 agonist had a dose-dependent effect increasing invasion (*n*=3 biological replicates, *P<0.05, unpaired, two-tailed Student's *t*-test).

To begin to explore the role of CTB CNR1 within the uterine wall, we confirmed the cell expression of this receptor by using antibody-based methods. Immunolocalization with an antibody that was specific for this antigen showed binding to a subset of cytokeratin (CK)-positive CTBs within the uterine wall and in the endovascular compartment (Fig. 6A-C). Immunoblotting with the same antibody detected a band of the expected molecular weight in CTB lysates (Fig. 6D).

Next, we measured the absolute amounts of the major mammalian endogenous cannabinoids, anandamide (AEA) and 2-arachidonoylglycerol (2-AG), in isolated CTBs and biopsies of the maternal-fetal interface that included invasive CTBs (Fig. 6E). AEA was detected (50-100 pg/10^6^ cells) and 2-AG was present at much higher concentrations (1000-5000 pg/10^6^ cells).

We then explored CNR1 function in terms of CTB invasion. CNR1 and CNR2 (CB2) agonists and antagonists (see Table S2) were added to the assay. Among the compounds tested, only a CNR1 agonist had a dose-dependent effect, increasing invasion (Fig. 6F).

## DISCUSSION

Here, we used a laser capture approach combined with analyses at RNA and protein levels to learn more about the biology of human trophoblast subtypes during the second trimester of pregnancy. Our focus was on cells that are difficult to isolate by other means. Thus, much of the existing data are from analysis of tissue samples in which the cell type of interest is only a relatively minor component (e.g. biopsies of the placenta or placental bed) or from *in vitro* models (e.g. cell lines) whose fidelity to the cognate cell type(s) *in vivo* is largely unproven. Global transcriptional profiling of microdissected syncytium, cell columns and endovascular compartments revealed many expected identifications as well as those that were, to the best of our knowledge, previously undescribed.

With regard to STBs, we expected to detect, at RNA and protein levels, well known abundant products such as *CSHL1* (chorionic somatomammotropin hormone-like 1), *CYP19A1* and *GH2*. We were also interested to find the expression of novel molecules such as neurotensin. Immunostaining for this molecule showed a vesicle-like pattern (Fig. 5A-C) with the stronger signal in the apical regions of the cells, suggesting release into maternal blood where the circulating molecule could induce systemic effects. Neurotensin was first isolated based on its ability to lower blood pressure (Carraway and Leeman, 1973). Later on, its role in promoting prolactin release and inhibiting luteinizing hormone secretion was discovered (McCann et al., 1982). Since then, other effects on the gut and brain have been described, which could account for a portion of the physiologic changes that occur during pregnancy (Bugni et al., 2012; Cordomi et al., 2016; Pérez de Vega et al., 2018; Tschumi and Beckstead, 2019). It would be very interesting to assay levels of this peptide in normal pregnancy and pregnancy complications with vascular (e.g. preeclampsia) or gastrointestinal (e.g. hyperemesis gravidarum) signs.

With regard to column CTBs, the highly-expressed differentially expressed molecules included pannexin 1. To our knowledge, placental expression has not been described. The structure and diverse roles of the three mammalian pannexins was recently reviewed (Makarenkova et al., 2018; Penuela et al., 2013; Whyte-Fagundes and Zoidl, 2018). These gap junction hemichannels release relatively small amounts of ATP under steady state conditions, which can substantially increase in pathological situations. In parallel, their roles vary from protective to harmful. Within these broad categories are many functions that are relevant to the initial stages of CTB invasion in which the cells form aggregates (i.e. columns) that connect the placenta to the uterus. In parallel, they execute an unusual differentiation program in which they transition from an epithelial to a vascular/mesenchymal phenotype. In keeping with the structural and functional changes CTBs undergo in columns, pannexin 1 has numerous physiologic functions, mediated by low levels of ATP release that could facilitate column formation, such as promoting differentiation and survival. However, stressors such as mechanostimulation can ramp up these levels producing inflammation and pyroptotic cell death.

Other novel identifications, at mRNA and protein levels, were associated with column CTBs. They included PGPEP1, an exopeptidase (Fig. 5G-I) that cleaves N-terminal pyroglutamyl residues (Dando et al., 2003), a modification that protects many chemokines and peptide hormones (gastrin, thyrotropin releasing hormone and neurotensin) from degradation and inactivation. In this regard, it is interesting to note that column CTBs make an enzyme that degrades an STB product (e.g. neurotensin), suggesting that they might be immune to its effects. Other novel mRNAs/proteins that were upregulated in this population included LY6K, which marks the progression of many cancers and has been implicated in immune escape (Upadhyay, 2019).

Trophoblasts within the endovascular compartment were notable for the expression of CNR1. The history of the endocannabinoid signaling field was recently reviewed (Maccarrone et al., 2015). Establishing the lipid Δ⁹-tetrahydrocannabinol as the psychoactive ingredient in cannabis enabled identification of a receptor, CNR1, which was the entry into the signaling pathways this ligand activates. Subsequently, a second receptor (CNR2) was described and there may be others. The existence of an exogenous ligand led to the discovery of endogenous lipids that bind these receptors, e.g. AEA and 2-AG, which are produced from multiple membrane lipids whenever the need arises. In this regard, we used a mass spectrometry approach to quantify AED and 2-AG in isolated CTBs and biopsies of the maternal-fetal interface that included invasive CTBs. In both cases, 2-AG was the dominant species (Fig. 6E).

Most of the work on endocannabinoid signaling during pregnancy has been carried out in the mouse (reviewed by Sun and Dey, 2012). On the maternal side, deletion of *Cnr1* and *Cnr2* reduces fertility secondary to impaired implantation owing to heightened edema around the implantation site (Li et al., 2019). In contrast, prolongation of endocannabinoid signaling results in premature decidual aging and preterm birth (Sun et al., 2016). On the embryo side, aberrant endocannabinoid signaling triggers premature trophoblast stem cell differentiation, and consequently, impairs trophoblast invasion. As a result, fetal development is retarded (Sun et al., 2010).

Together, these results suggest that this signaling system could play an important role in human placentation. Thus, we were interested to find that endovascular CTBs expressed *CNR1*. Analyses at the protein level showed widespread expression of this receptor among invasive CTBs. As a result, we asked whether agonists or antagonists impacted invasion, assayed by an *in vitro* model of this process. Unexpectedly, we found that a CNR1 agonist substantially increased invasion. Of the many molecules whose functions we have tested in this system, very few have had this effect. Notably, perturbing integrin α5/β1 interactions with fibronectin also increased invasion, suggesting that CTBs have systems for accelerating and braking invasion (Damsky et al., 1994). With regard to physiological regulators, we showed that uterine hypoxia, produced by aortic constriction, very significantly increases CTB invasion in the rhesus macaque (Zhou et al., 1993). Whether the parallel increases in cannabinoid signaling and invasion are related to either of these observations remains to be determined.

In summary, our LMD/transcriptomic analysis of human STBs and invasive CTBs identified the expression of mRNAs encoding molecules known to be expressed by each cell type as well as novel transcripts that were also differentially expressed at the protein level. By using a mass spectrometry-based approach to validate DEGs we also confirmed that *C4ORF36* is translated. Immunolocalization revealed a cytoplasmic staining pattern (Fig. 5D-F) that was consistent with the function of its fungal protein analog, dynein light chain, which is involved in intracellular transport (Li et al., 2018). We also showed that invasive CTBs express CNR1. Furthermore, endocannabinoid signaling in these cells appears to increase invasion, suggesting that this system may play the important role of facilitating deep placentation in human pregnancy. This finding raises the possibility that exogenous cannabinoids could also impact CTB invasion. Given the rapid rate of marijuana legalization in the US combined with the increased availability of synthetic cannabinoids with enhanced activity, the results of our study suggest that women should be cautioned against using these drugs during pregnancy.

## MATERIALS AND METHODS

### Tissue collection and preparation; CTB isolation

The University of California, San Francisco (UCSF) Institutional Review Board (Committee on Human Research) approved this study. Written informed consent was obtained from all donors. Samples were processed within 1 h of obtaining the tissue. Biopsies of the maternal-fetal interface were taken from four placentas with gestational ages stratified across the second trimester (15, 17, 18 and 20 weeks). Spiral arteries from the same cases were identified using a dissection microscope (Leica MZ16). Biopsied specimens (1×1×0.5 cm) were rinsed in ice-cold PBS, transferred to a 1:1 (v:v) PBS:OCT (Optimal Cutting Temperature compound, Thermo Fisher Scientific) semi-frozen slurry for 15 min, oriented in an OCT-filled peel-a-way disposable embedding mold (Thermo Fisher Scientific) and frozen over a mixture of dry ice and ethanol. For immunolocalization, biopsies were fixed [3% paraformaldehyde in PBS (wt/vol)] and passed through a sucrose gradient (5, 10 and 15% in PBS). Then they were placed in a mold and frozen in OCT as described above. For immunoblotting, unfixed specimens were frozen in a mortar over dry ice, pulverized and stored at −80°C.

CTBs were isolated from 2nd trimester placentas as previously described (Hunkapiller and Fisher, 2008). Briefly, chorionic villi were subjected to a series of enzymatic digestion steps that sequentially removed the outer STB layer and the underlying CTBs, which were isolated by centrifugation on a Percoll density gradient. Only cell preparations that were >80% pure as determined by CK staining were used in experiments.

### Laser microdissection

Frozen blocks were sectioned (−20°C) using a Leica CM3050 cryostat. Sections (20 μm) were mounted on different slides depending on the intended analysis. For subsequent RNA isolation, we used poly-L-lysine (Sigma Chemical Company)-coated, UV-treated, metal-frame, polyethylene naphthalate-membrane (PEN) slides (Thermo Fisher Scientific). For subsequent protein isolation, we used poly-L-lysine-treated Director slides (Expression Pathology; Hembrough et al., 2012). Slides with sections were kept under dry ice until LMD the same day. Sections on slides were manually defrosted in room air (30 s), immersed in PBS until the OCT was completely removed (∼2 min), dipped in 0.1% Toluidine Blue for 30 s, washed in ice-cold PBS, dehydrated (30 s/treatment) in a graded ethanol series (75%×2, 95%, 100%), then rapidly dried with compressed nitrogen. All solutions were made with nuclease-free reagents for RNA isolation or HPLC grade solutions for protein isolation in sterile single-use 50 ml conical tubes (Falcon). Regions of interest were identified using a 20× objective and circled on a touch-screen monitor (Wacom) coupled to a color camera (Leica DFC310 FX), which triggered a solid state UV laser (LMD 7000) enabling dissection (samples for RNA isolation) or rasterisation (samples for protein isolation) into a buffer-filled cap suspended immediately below. Buffer RLT Plus (Qiagen) was used for subsequent RNA isolation. An alkaline surfactant-containing solution [50 mM triethylammonium bicarbonate (Sigma-Aldrich), 5 mM tris(2-carboxyethyl)phosphine (Bond-Breaker, Thermo Fisher Scientific), and 0.1% (w/v) RapiGest SF (Waters)] was used for subsequent protein isolation.

For RNA, ten slides with four sections per slide were prepared for each specimen. For protein, five slides with eight sections per slide were prepared. To limit degradation, each slide used for RNA isolation was microdissected for no more than 30 min, which was extended to 1 h for protein recovery. The regions of interest were collected into different caps and pooled across all 40 tissue sections.

### RNA isolation

Total RNA was isolated according to the manufacturer's protocol (Qiagen RNeasy Plus Micro Kit) with the added step of an on-column DNase digestion of bound RNA to avoid any DNA contamination. The concentration of total RNA was measured photometrically (NanoDrop 2000c). RNA integrity was determined via microfluidic phoresis (Agilent Tapestation). The samples were stored at −80°C before analysis.

### Global RNA profiling

We used a microarray approach (Affymetrix GeneChip HuGene 2.0 ST) and protocols that were devised by the UCSF Gladstone (National Heart, Lung, and Blood Institute) Genomics Core Facility. Gene expression data quality was confirmed, robust multiarray averaging (RMA) normalized (Bolstad et al., 2003; Irizarry et al., 2003; Wu et al., 2004) and summarized (oligo package) (Carvalho and Irizarry, 2010) in R/Bioconductor (Ihaka and Gentleman, 1996; R Core Team, 2013). The data were modeled and contrasts between STB, CTB and ENDO determined using the limma package (Ritchie et al., 2015). DEGs had an absolute linear fold change ≥2 and an adjusted *P*-value<0.05 for any pairwise comparison (STB versus CTB, STB versus ENDO, ENDO versus CTB). Four biological replicates of each subpopulation were analyzed (15, 17, 18 and 20 weeks gestation). The data were deposited in GEO (accession number GSE156766).

### Global protein profiling: trophoblast capture; protein isolation and digestion

Three biological replicates of each cell type (STB, CTB and ENDO; 17, 18, and 20 weeks gestation) were isolated by LMD from the same placentas that were subjected to global transcriptional profiling (see previous section). The samples were incubated in the alkaline surfactant-containing solution described above at 60°C with rigorous vortexing every 15 min for 1 h. Iodoacetamide (Thermo Fisher Scientific) was added to 15 mM and incubated at room temperature in the dark for 30 min. Proteins were digested overnight at 37°C with trypsin (20 ng/µl, Pierce), then centrifuged at 16,000 ***g*** (Eppendorf) for 10 min. Trifluoroacetic acid (Pierce) was added to the supernatant such that the final concentration was 0.5%. Duplicate technical replicates were performed along with a control that consisted of a blank DIRECTOR slide that was carried through the entire sample preparation protocol.

### Global protein profiling: identification, relative quantification and tandem assembly

Using published methods (Branson and Freitas, 2016), each trypsin digestion was analyzed by reversed phase high performance liquid chromatography tandem mass spectrometry (RP HPLC MS/MS) using a nanoLC Ultra system (Eksigent Technologies) interfaced with a LTQ Orbitrap Velos mass spectrometer (Thermo Fisher Scientific). Peptides were separated using an Acclaim PepMap100 C18 column (75 µm i.d.×15 cm, 3 µm particle size, 100 Å pores, Thermo Fisher Scientific) via mobile phase A (acetonitrile/water, 2:98, v/v, 0.1% formic acid) and mobile phase B (acetonitrile/water, 98:2, v/v, 0.1% formic acid) in conjunction with a linear gradient of 2-40% B over 90 min. The mobile phase flow rate was 550 nl/min. Using electrospray ionization, the LTQ Orbitrap Velos was operated in data-dependent acquisition mode for MS and MS/MS data collection. An initial survey scan was acquired (m/z 300-1500) in the Orbitrap analyzer at mass resolution 30,000, followed by collision-induced dissociation of precursor ions in the ion trap to produce MS/MS spectra for the 20 most abundant precursor ions. The instrument was operated with an isolation width of 3.0, normalized collision energy of 35, an ActQ of 0.25, and an MS/MS activation time of 10 ms.

The DEGs observed in the mRNA data were used to create a FASTA database of reviewed, canonical protein sequences (Pundir et al., 2017). Protein identification was accomplished by searching this database with the peptide MS/MS spectra using MS Amanda 2.0 and Sequest HT search algorithms (Proteome Discoverer, v2.2). The false discovery rate (FDR) was controlled by Percolator, a semi-supervised machine learning algorithm (Käll et al., 2007), using a target-decoy database strategy (Elias and Gygi, 2010). Criteria for identified proteins (IPs) included a minimum of two unique corresponding peptide spectra counted at least twice each, in two or more biological replicates, at an FDR<0.10% (Proteome Discoverer, v2.2).

All subsequent analyses were carried out in R (Ihaka and Gentleman, 1996; R Core Team, 2013). The spectral counts were quantified (Branson and Freitas, 2016) and modeled using negative binomials (Robinson and Smyth, 2008). The differentially expressed proteins (STB versus CTB, STB versus ENDO, ENDO versus CTB) were identified using the exact test in edgeR (Robinson et al., 2010) (*P*<0.05). The coordinately differentially expressed genes and proteins were termed tandems.

All raw files have been uploaded to the Center for Computational Mass Spectrometry, MassIVE (https://massive.ucsd.edu), dataset MSV000085995. Data uploads include the protein identification details.

### Ingenuity pathway analysis

The tandems for each trophoblast subtype were uploaded to IPA and a core expression analysis performed, followed by a comparison analysis of the results. A significant canonical pathway had a −log(*P*-value)>1.3, an absolute *z*-score>2, and contained at least four tandems in a unique combination (no pathway redundancy).

### Immunolocalization

Portions of second trimester placentas that included anchoring chorionic villi were fixed in 3% paraformaldehyde for 30 min, washed three times in PBS, infiltrated with 5 to 15% sucrose followed by OCT medium, and frozen in liquid nitrogen. Tissues were sectioned (10 μm) using a cryostat (Leica CM3050) and collected on charged glass slides (Thermo Fisher Scientific). The OCT was removed by washing in ice-cold PBS. Nonspecific binding was inhibited by incubation for 30 min. with blocking buffer: 3% (w/v) bovine serum albumin (BSA), 0.1% cold fish skin gelatin, 0.1% Triton X-100 and 0.05% Tween 20 (Thermo Fisher Scientific) in PBS. Primary and secondary antibodies, their sources/catalogue numbers, the species in which they were raised and the concentrations used in the immunolocalization experiments are listed in Table S1. As to the general methods, a primary unconjugated antibody and rat anti-cytokeratin were diluted together in blocking buffer, incubated overnight with tissue sections in a humidified chamber at 4°C, washed (3×10 min) in PBS, incubated with species-specific fluorescently tagged secondary antibodies and washed again in PBS. Mounting medium with 4′,6-diamidino-2-phenylindole (DAPI; Vector Laboratories) was applied and the sections were coverslipped. Controls included omission of the primary or secondary antibody. The tissue sections were examined and images captured using a confocal Leica DM5000 B microscope. *Z*-stacks were created in 1 µm increments at 1024×1024 pixel resolution and line averaged three times.

### Immunoblotting

Proteins from isolated CTB cell pellets were solubilized in M-PER (Thermo Fisher Scientific) according to the manufacturer's protocol and protein concentrations determined spectrophotometrically (Shimadzu). Samples (5 μg) were electrophoretically separated on 4-12% Bis-Tris protein gels (1.5 mm, Invitrogen) and transferred to nitrocellulose membranes, followed by blocking for 1 h with 5% nonfat powdered milk in PBS-Tween-20 (0.1%) before incubating overnight at 4°C with primary antibody. Then the membranes were washed three times in PBS-Tween-20 and incubated with the appropriate species-specific secondary antibody for 1 h. Immunoreactive bands were detected with ECL 2 Western Blotting Substrate (Thermo Fisher Scientific, Pierce) and ECL high performance chemiluminescence film (Amersham, GE Healthcare). Equal sample loading in all the lanes was confirmed by anti-α-actin immunoreactivity.

### Mass spectrometry quantification of endogenous cannabinoids (AEA and 2-AG)

Cell or tissue (maternal-fetal interface) samples were prepared using a Precellys 24 homogenizer with a Cryolys cooling unit (Bertin Technologies). Individual samples were placed in 2 ml vials with 10 vol water (10-fold dilution) and seven homogenization beads. Homogenization was conducted for three cycles (20 s each) at 5000 rpm (with 30 s breaks) at <10°C. Then 50 µl of each homogenized sample was pipetted into 13×100 mm tubes after which 150 µl of a deuterated internal standard solution was added, before vortexing for 1 min and centrifugation (3000 ***g***) for 10 min.

Then the supernatant (5 µl) was separated on a Shimadzu Prominence UFLC system equipped with a binary pump and a SIL-20AC autosampler. Separation was achieved on an XBridge C18 column (4.6×50 mm). A gradient separation was used consisting of mobile phase A [0.05% H_2_O/5 mM MNH_4_ (15%)] and mobile phase B [ACN/2.5 mM MNH_4_ (85%)] at a flow rate of 1.0 ml/min.

Mass spectrometric detection was performed using an Applied Biosystems/MDS SCIEX API 5000 triple quadrupole mass spectrometer, which was operated in multiple reaction monitoring (MRM) mode via the negative electrospray ionization interface using the transitions (*m/z*) 379.4→287.4 for 2-AG, 386.4→293.4 for 2-AG-d8, 348.5→62.2 for AEA, and 356.5→163.2 for AEA-d8. Data acquisition and quantitative processing were accomplished using Applied Biosystems Analyst version 1.5.1 software.

### Invasion assay

Cytotrophoblasts (2nd trimester) were isolated according to methods described above. The cells (250,000) were plated in 500 µl of DME H-21/2% Nutridoma on Matrigel-coated 24-well Transwell inserts (Costar) with 8.0 mm pores for 1 h. Nonadherent cells were removed by washing with warm medium before adding DME H-21/2% Nutridoma containing 1 or 10 ng/ml of a CNR1 or CNR2 agonist or antagonist, or the vehicle in which they were dissolved as a control (0.1% Tocrisolve, Tocris). The compounds and their activities are shown in Table S2. Each condition was tested in triplicate. After culturing for 72 h, the inserts were washed with PBS and fixed for 20 min in 3% PFA. They were stored at 4°C in PBS or immediately immunostained as described above with anti-cytokeratin 7. Then the membrane inserts were removed with surgical scissors and mounted on slides with DAPI-containing medium such that the underside of the membrane faced up. They were imaged with a fluorescent microscope (Leica DM3000B). The number of cytotrophoblast projections that reached the underside of the filter was counted and the data were expressed as experimental values relative to controls. The assay was carried out three times (biological replicates). An unpaired, two-tailed Student's *t*-test (*P*<0.05) was used to determine significance.

### Creation of graphics

Tables and heatmaps were constructed in Excel (Microsoft) and scatter and parallel plots were generated in OriginPro (OriginLabs).

## Supplementary Material

Supplementary information

Reviewer comments
