# Supplementary material for: RNA profiling of laser microdissected human trophoblast subtypes at mid-gestation reveals a role for cannabinoid signaling in invasion
Source: Development. 2021 Oct 19;148(20):dev199626. doi: 10.1242/dev.199626 (PMC8572005; doi:10.1242/dev.199626)
Supplement: Supplementary information [file develop-148-199626-s1.pdf]

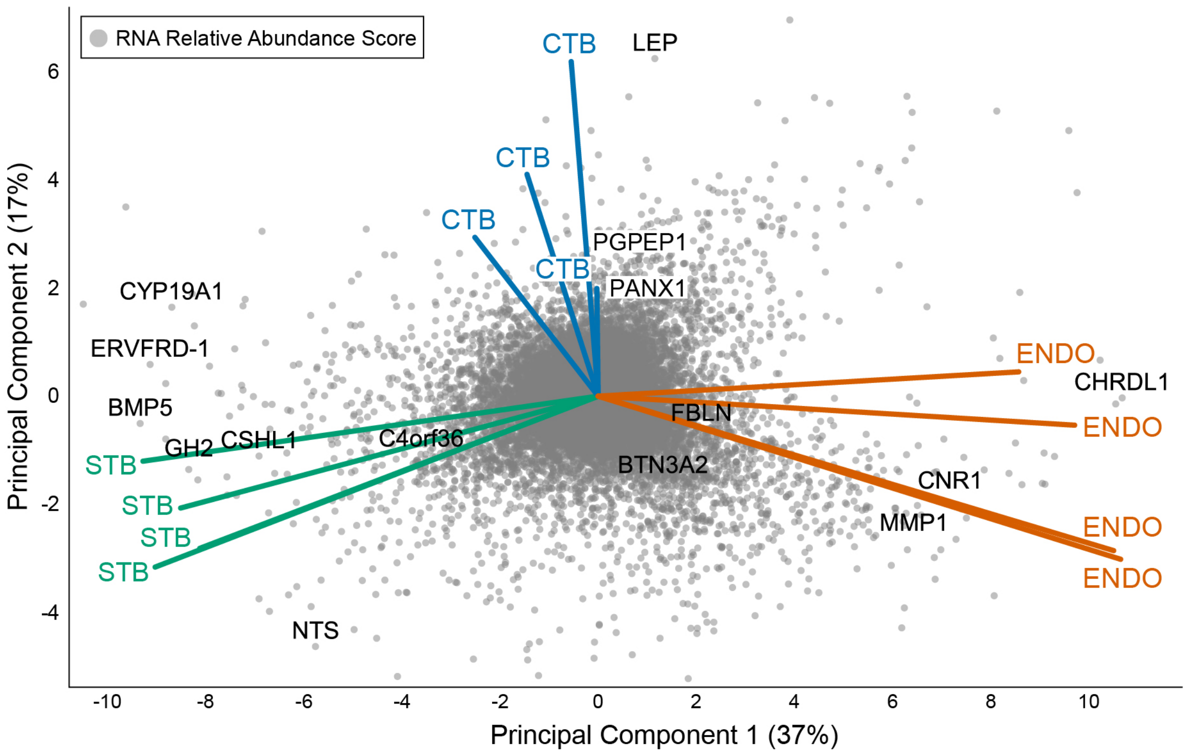

Fig. S1.

Development • Supplementary information

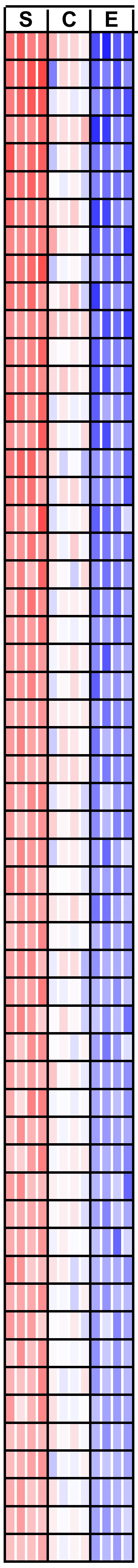

Symbol	Name	Linear Fold Change (*ns*=no significant difference) S vs C	S vs E	E vs C
ERVFRD-1	endogenous retrovirus group FRD, member 1	3.5	77.7	-21.9
GH2	growth hormone 2	11.6	64.7	-5.6
SVEP1	sushi, von Willebrand factor type A, EGF and pentraxin domain containing 1	9.3	57.8	-6.2
PLAC4	placenta specific 4	2.7	49.8	-18.1
KISS1	KiSS-1 metastasis-suppressor	9.0	44.5	-4.9
LGR5	leucine-rich repeat containing G protein-coupled receptor 5	8.1	41.2	-5.1
LOC100129935	lectin, galactoside-binding, soluble, 14 pseudogene	4.0	41.0	-10.2
LRP2	LDL receptor related protein 2	3.4	39.5	-11.5
TRPV6	transient receptor potential cation channel, subfamily V, member 6	10.3	39.5	-3.8
INSL4	insulin-like 4 (placenta)	4.3	37.9	-8.8
PEG10	paternally expressed 10	2.3	34.2	-15.0
LINC00967	long intergenic non-protein coding RNA 967	4.8	34.0	-7.1
DEPDC1B	DEP domain containing 1B	3.0	33.2	-11.2
CSHL1	chorionic somatomammotropin hormone-like 1	6.6	30.7	-4.7
LCMT1-AS2	LCMT1 antisense RNA 2	4.0	29.6	-7.4
ANKRD20A5P	ankyrin repeat domain 20 family, member A5, pseudogene	8.0	27.7	-3.5
KMO	kynurenine 3-monooxygenase (kynurenine 3-hydroxylase)	5.4	26.6	-5.0
PSG8	pregnancy specific beta-1-glycoprotein 8	5.4	26.5	-4.9
TCL6	T-cell leukemia	3.6	24.3	-6.8
GABRE	gamma-aminobutyric acid (GABA) A receptor, epsilon	4.2	21.2	-5.1
LOC100128988	uncharacterized LOC100128988	4.4	21.1	-4.8
SEMA6A	semaphorin 6A	4.4	18.5	-4.2
CRYBG1	crystallin beta-gamma domain containing 1	3.3	18.2	-5.5
PSG9	pregnancy specific beta-1-glycoprotein 9	4.2	17.3	-4.1
GPC3	glypican 3	2.8	16.2	-5.8
LOC102724687	uncharacterized LOC102724687	3.8	15.7	-4.1
FAM184A	family with sequence similarity 184, member A	2.1	15.0	-7.3
HSD17B1	hydroxysteroid (17-beta) dehydrogenase 1	4.4	14.4	-3.2
PKIB	protein kinase (cAMP-dependent, catalytic) inhibitor beta	2.8	14.2	-5.1
ERV3-1	endogenous retrovirus group 3, member 1	4.3	13.5	-3.1
MPP7	membrane protein, palmitoylated 7	3.8	13.3	-3.5
EXPH5	exophilin 5	2.4	13.3	-5.6
IGSF5	immunoglobulin superfamily, member 5	3.4	12.1	-3.6
ABCG2	ATP binding cassette subfamily G member 2 (Junior blood group)	3.9	11.9	-3.1
SLC13A4	solute carrier family 13 (sodium	5.2	11.8	-2.3
MICU3	mitochondrial calcium uptake family, member 3	3.4	11.8	-3.4
SLC13A3	solute carrier family 13 (sodium-dependent dicarboxylate transporter), member 3	3.5	11.2	-3.2
LINC00845	long intergenic non-protein coding RNA 845	2.4	10.9	-4.6
HSD11B2	hydroxysteroid (11-beta) dehydrogenase 2	3.8	10.9	-2.9
ESRRG	estrogen-related receptor gamma	2.9	10.8	-3.7
SLC43A2	solute carrier family 43 (amino acid system L transporter), member 2	2.6	10.7	-4.1
LOC105370058	uncharacterized LOC105370058	3.0	10.4	-3.5
ANK3	ankyrin 3, node of Ranvier (ankyrin G)	3.1	10.4	-3.4
LOC105369669	uncharacterized LOC105369669	3.3	10.4	-3.2
LOC105379298	uncharacterized LOC105379298	3.3	10.2	-3.1
TACC2	transforming, acidic coiled-coil containing protein 2	3.9	10.0	-2.6
PSG10P	pregnancy specific beta-1-glycoprotein 10, pseudogene	3.2	9.7	-3.0
SEMA6A-AS1	SEMA6A antisense RNA 1	3.2	9.5	-2.9
PLEKHA6	pleckstrin homology domain containing, family A member 6	2.9	9.3	-3.2
CKMT1B	creatine kinase, mitochondrial 1B	2.8	9.0	-3.2
RYBP	RING1 and YY1 binding protein	2.1	8.3	-4.0
LOC255187	uncharacterized LOC255187	3.8	8.1	-2.1
MIR503HG	MIR503 host gene	3.1	7.9	-2.6
LOC105370057	uncharacterized LOC105370057	2.4	7.3	-3.0
ZFAT	zinc finger and AT hook domain containing	2.2	7.2	-3.3

Development • Supplementary information

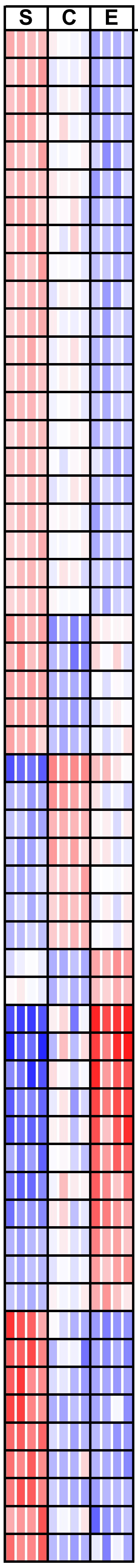

Symbol	Name	Linear Fold Change (*ns*=no significant difference) S vs C	S vs E	E vs C
PLEKHH1	pleckstrin homology domain containing, family H (with MyTH4 domain) member 1	2.7	7.1	-2.6
ARHGAP26	Rho GTPase activating protein 26	3.1	7.0	-2.3
BPGM	2,3-bisphosphoglycerate mutase	2.9	6.8	-2.4
SLC22A3	solute carrier family 22 (organic cation transporter), member 3	2.4	6.7	-2.8
NHSL1	NHS-like 1	2.5	6.6	-2.6
PSG3	pregnancy specific beta-1-glycoprotein 3	2.1	6.1	-2.9
GGH	gamma-glutamyl hydrolase (conjugase, folylpolygammaglutamyl hydrolase)	2.5	6.1	-2.4
LOC105377029	uncharacterized LOC105377029	2.6	6.0	-2.3
SIAH1	siah E3 ubiquitin protein ligase 1	2.7	5.9	-2.2
POGLUT3	protein O-glucosyltransferase 3	2.5	5.6	-2.3
TBX3	T-box 3	2.5	5.2	-2.1
GULP1	GULP, engulfment adaptor PTB domain containing 1	2.2	5.2	-2.3
NECTIN3	nectin cell adhesion molecule 3	2.2	5.2	-2.4
KDM7A	lysine (K)-specific demethylase 7A	2.5	5.1	-2.0
GRIP1	glutamate receptor interacting protein 1	2.1	4.9	-2.3
ACADL	acyl-CoA dehydrogenase, long chain	2.3	4.8	-2.1
MAN1C1	mannosidase, alpha, class 1C, member 1	2.1	4.7	-2.3
ADRB1	adrenoceptor beta 1	2.1	4.7	-2.2
CAP2	CAP, adenylate cyclase-associated protein, 2 (yeast)	2.1	4.7	-2.2
LNPEP	leucyl	2.3	4.6	-2.0
DUSP16	dual specificity phosphatase 16	2.1	4.3	-2.1
TMEM150C	transmembrane protein 150C	12.7	2.6	4.9
GUCY1B1	guanylate cyclase 1 soluble subunit beta 1	10.9	2.5	4.3
FAM162B	family with sequence similarity 162, member B	9.2	3.6	2.5
KL	klotho	8.4	3.4	2.5
DACH1	dachshund family transcription factor 1	6.5	2.9	2.3
FSTL3	follistatin-like 3 (secreted glycoprotein)	-38.5	-14.2	-2.7
SLC2A3	solute carrier family 2 (facilitated glucose transporter), member 3	-9.5	-2.3	-4.1
ZNF486	zinc finger protein 486	-7.4	-2.8	-2.7
SLCO4A1	solute carrier organic anion transporter family, member 4A1	-7.0	-2.6	-2.7
CLDN19	claudin 19	-5.5	-2.3	-2.4
CXCR6	chemokine (C-X-C motif) receptor 6	-5.1	-2.0	-2.5
IL1RAP	interleukin 1 receptor accessory protein	-5.0	-2.2	-2.3
SYT11	synaptotagmin XI	2.2	-4.3	9.4
HDGFL3	HDGF like 3	2.4	-2.4	5.8
CHRDL1	chordin-like 1	-8.7	-191.1	22.0
PRL	prolactin	-10.3	-150.8	14.7
HLA-DRA	major histocompatibility complex, class II, DR alpha	-7.8	-74.8	9.6
RORB	RAR-related orphan receptor B	-4.1	-63.4	15.7
IL1RL1	interleukin 1 receptor-like 1	-7.3	-59.9	8.2
IFI44L	interferon-induced protein 44-like	-3.4	-31.5	9.1
GKN1	gastrokine 1	-8.3	-29.7	3.6
F2R	coagulation factor II (thrombin) receptor	-4.2	-23.1	5.5
GPRC5B	G protein-coupled receptor, class C, group 5, member B	-2.3	-9.2	4.0
TMEM45A	transmembrane protein 45A	-2.1	-8.5	4.1
PDE6H	phosphodiesterase 6H, cGMP-specific, cone, gamma	-2.5	-6.2	2.4
AREG	amphiregulin	15.4	34.9	*ns*
SLC27A2	solute carrier family 27 (fatty acid transporter), member 2	18.4	31.6	*ns*
SCN7A	sodium channel, voltage gated, type VII alpha subunit	15.9	25.0	*ns*
NTS	neurotensin	23.3	21.1	*ns*
MEOX2	mesenchyme homeobox 2	17.0	23.2	*ns*
SLC26A7	solute carrier family 26 (anion exchanger), member 7	11.3	21.1	*ns*
LOC105375166	uncharacterized LOC105375166	19.4	20.4	*ns*
SH3TC2	SH3 domain and tetratricopeptide repeats 2	5.7	18.2	*ns*
APLNR	apelin receptor	18.1	14.2	*ns*

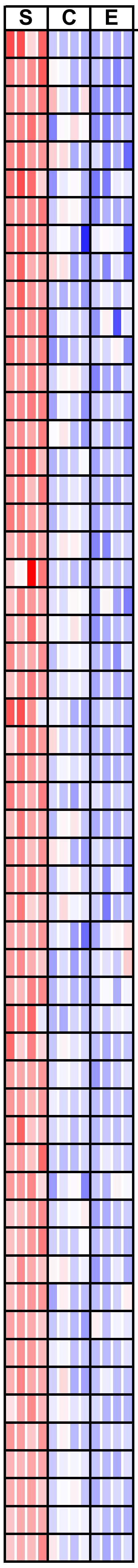

Symbol	Name	Linear Fold Change (*ns*=no significant difference) S vs C	S vs E	E vs C
CPS1	carbamoyl-phosphate synthase 1	15.0	18.0	*ns*
PLA2G2A	phospholipase A2, group IIA (platelets, synovial fluid)	8.5	17.5	*ns*
MIR4713	microRNA 4713	4.3	16.9	*ns*
GSTA3	glutathione S-transferase alpha 3	6.2	16.5	*ns*
SNORD113-4	small nucleolar RNA, C	5.7	16.2	*ns*
LOC105372578	uncharacterized LOC105372578	10.8	15.2	*ns*
LOC105369382	uncharacterized LOC105369382	6.6	15.0	*ns*
P2RY1	purinergic receptor P2Y, G-protein coupled, 1	13.8	9.1	*ns*
SNORD113-5	small nucleolar RNA, C	6.3	13.1	*ns*
LOC101927482	uncharacterized LOC101927482	12.7	7.7	*ns*
LINC00474	long intergenic non-protein coding RNA 474	9.0	12.6	*ns*
HIGD1B	HIG1 hypoxia inducible domain family, member 1B	12.2	8.4	*ns*
USP27X	ubiquitin specific peptidase 27, X-linked	4.7	11.9	*ns*
ABCB1	ATP binding cassette subfamily B member 1	11.7	11.0	*ns*
SNORD113-1	small nucleolar RNA, C	6.9	11.4	*ns*
TTPA	tocopherol (alpha) transfer protein	8.4	11.3	*ns*
AGTR1	angiotensin II receptor, type 1	7.1	10.7	*ns*
LOC730101	uncharacterized LOC730101	6.9	10.7	*ns*
ZNF554	zinc finger protein 554	4.1	10.6	*ns*
MUCL1	mucin-like 1	10.1	9.9	*ns*
ISM1-AS1	ISM1 antisense RNA 1	4.3	9.9	*ns*
GNGT1	guanine nucleotide binding protein (G protein), gamma transducing activity polypeptide 1	4.1	9.8	*ns*
SMAGP	small cell adhesion glycoprotein	5.4	9.7	*ns*
ENDOU	endonuclease, polyU-specific	5.2	9.6	*ns*
CRISPLD1	cysteine-rich secretory protein LCCL domain containing 1	8.7	9.6	*ns*
MIR181B1	microRNA 181b-1	5.1	9.6	*ns*
LOC100128386	uncharacterized LOC100128386	5.4	9.5	*ns*
COX4I2	cytochrome c oxidase subunit IV isoform 2 (lung)	9.5	8.5	*ns*
LOC100129345	uncharacterized LOC100129345	4.8	9.2	*ns*
SNHG24	small nucleolar RNA host gene 24	5.7	9.0	*ns*
PHYHIPL	phytanoyl-CoA 2-hydroxylase interacting protein-like	5.3	8.9	*ns*
SNORD114-31	small nucleolar RNA, C	3.5	8.9	*ns*
HSD17B2	hydroxysteroid (17-beta) dehydrogenase 2	8.8	4.1	*ns*
ADAMTS19	ADAM metallopeptidase with thrombospondin type 1 motif 19	8.6	4.0	*ns*
PCDH11X	protocadherin 11 X-linked	8.5	6.3	*ns*
CRHBP	corticotropin releasing hormone binding protein	8.4	5.5	*ns*
IL22RA2	interleukin 22 receptor, alpha 2	4.9	8.3	*ns*
ZNF91	zinc finger protein 91	5.1	8.2	*ns*
FRAS1	Fraser extracellular matrix complex subunit 1	5.4	8.0	*ns*
COLEC10	collectin sub-family member 10 (C-type lectin)	6.4	7.9	*ns*
EGFEM1P	EGF-like and EMI domain containing 1, pseudogene	7.7	6.8	*ns*
GCNT4	glucosaminyl (N-acetyl) transferase 4, core 2	7.7	5.1	*ns*
TRIM40	tripartite motif containing 40	3.6	7.7	*ns*
TBX5	T-box 5	5.5	7.7	*ns*
LOC105376429	uncharacterized LOC105376429	3.1	7.0	*ns*
LINC01336	long intergenic non-protein coding RNA 1336	6.9	6.1	*ns*
RAB38	RAB38, member RAS oncogene family	6.8	3.8	*ns*
EPHA3	EPH receptor A3	5.3	6.7	*ns*
SNORD114-28	small nucleolar RNA, C	6.3	6.7	*ns*
MINDY4B	MINDY family member 4B	3.4	6.6	*ns*
LOC100507661	uncharacterized LOC100507661	5.7	6.5	*ns*
SLC6A4	solute carrier family 6 (neurotransmitter transporter), member 4	4.1	6.4	*ns*
CLRN1	clarin 1	3.9	6.3	*ns*
MAP7D2	MAP7 domain containing 2	3.6	6.3	*ns*
MIR181A1HG	MIR181A1 host gene	4.0	6.3	*ns*

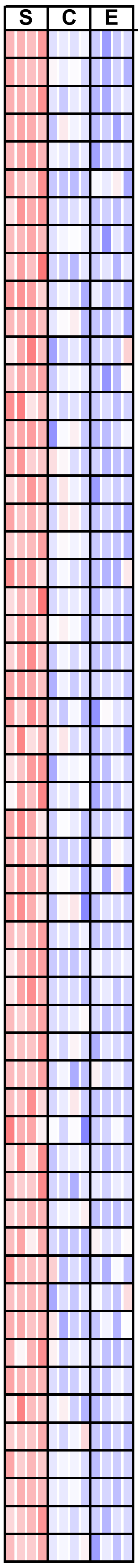

Symbol	Name	Linear Fold Change (*ns*=no significant difference) S vs C	S vs E	E vs C
MTMR10	myotubularin related protein 10	3.7	6.2	*ns*
LINC01118	long intergenic non-protein coding RNA 1118	3.1	6.2	*ns*
ACSS1	acyl-CoA synthetase short-chain family member 1	4.5	6.1	*ns*
C4orf36	chromosome 4 open reading frame 36	3.5	6.1	*ns*
LINC02365	long intergenic non-protein coding RNA 2365	4.0	6.1	*ns*
CCDC102B	coiled-coil domain containing 102B	6.1	3.8	*ns*
ADAMTS18	ADAM metallopeptidase with thrombospondin type 1 motif 18	4.5	5.9	*ns*
MED12L	mediator complex subunit 12 like	3.2	5.9	*ns*
AFF2	AF4	5.8	5.7	*ns*
HSPA4L	heat shock 70kDa protein 4-like	5.2	5.8	*ns*
AHR	aryl hydrocarbon receptor	3.5	5.8	*ns*
NRCAM	neuronal cell adhesion molecule	5.7	3.7	*ns*
SLC20A1	solute carrier family 20 (phosphate transporter), member 1	3.8	5.7	*ns*
HS6ST2	heparan sulfate 6-O-sulfotransferase 2	4.9	5.7	*ns*
SERPINB2	serpin peptidase inhibitor, clade B (ovalbumin), member 2	5.0	5.6	*ns*
CROT	carnitine O-octanoyltransferase	2.9	5.6	*ns*
FAM3B	family with sequence similarity 3, member B	3.1	5.4	*ns*
TCHH	trichohyalin	2.7	5.4	*ns*
MEG3	maternally expressed 3 (non-protein coding)	2.9	5.4	*ns*
SLC16A12	solute carrier family 16, member 12	4.2	5.3	*ns*
CADM3	cell adhesion molecule 3	4.0	5.3	*ns*
PMAIP1	phorbol-12-myristate-13-acetate-induced protein 1	3.0	5.3	*ns*
LOC105375161	uncharacterized LOC105375161	4.0	5.3	*ns*
ARHGAP42	Rho GTPase activating protein 42	5.3	4.9	*ns*
IGHA1	immunoglobulin heavy constant alpha 1	5.0	5.2	*ns*
WNT2	wingless-type MMTV integration site family member 2	3.0	5.2	*ns*
FLJ90680	FLJ90680 protein	3.7	5.1	*ns*
VXN	vexin	3.2	5.1	*ns*
TMEM133	transmembrane protein 133	4.2	5.0	*ns*
GPR183	G protein-coupled receptor 183	5.0	3.2	*ns*
AGPAT5	1-acylglycerol-3-phosphate O-acyltransferase 5	4.1	5.0	*ns*
TWIST1	twist family bHLH transcription factor 1	4.5	5.0	*ns*
ISM1	isthmin 1, angiogenesis inhibitor	3.8	4.9	*ns*
LOC101927139	uncharacterized LOC101927139	4.9	3.4	*ns*
MIR181A1	microRNA 181a-1	4.9	4.6	*ns*
SEMA3B	semaphorin 3B	3.2	4.8	*ns*
PART1	prostate androgen-regulated transcript 1 (non-protein coding)	3.3	4.8	*ns*
PTPRD	protein tyrosine phosphatase, receptor type, D	4.8	3.4	*ns*
OTOGL	otogelin-like	3.3	4.8	*ns*
CSTA	cystatin A (stefin A)	4.8	4.6	*ns*
NRAD1	non-coding RNA in the aldehyde dehydrogenase 1A pathway	3.8	4.8	*ns*
MPP1	membrane protein, palmitoylated 1	4.8	3.6	*ns*
AFF1	AF4	2.6	4.7	*ns*
ITGA9	integrin alpha 9	4.7	2.7	*ns*
BLID	BH3-like motif containing, cell death inducer	3.9	4.7	*ns*
SEL1L3	sel-1 suppressor of lin-12-like 3 (C. elegans)	4.7	3.1	*ns*
ARHGAP28	Rho GTPase activating protein 28	4.7	4.3	*ns*
CKMT1A	creatine kinase, mitochondrial 1A	3.1	4.6	*ns*
RFX6	regulatory factor X, 6	4.6	4.6	*ns*
SNORD114-25	small nucleolar RNA, C	3.9	4.6	*ns*
MYLIP	myosin regulatory light chain interacting protein	2.5	4.6	*ns*
CCK	cholecystokinin	3.0	4.6	*ns*
HACE1	HECT domain and ankyrin repeat containing E3 ubiquitin protein ligase 1	3.3	4.5	*ns*
CADM3-AS1	CADM3 antisense RNA 1	3.3	4.5	*ns*
PRKCZ	protein kinase C, zeta	2.6	4.5	*ns*

Development: doi:10.1242/dev.199626: Supplementary information

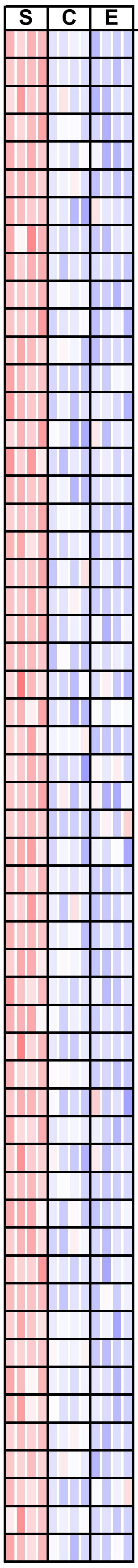

		Linear Fold Change (*ns*=no significant difference)		
Symbol	Name	S vs C	S vs E	E vs C
SH3BGRL2	SH3 domain binding glutamate-rich protein like 2	3.1	4.5	*ns*
FRMD6	FERM domain containing 6	3.6	4.5	*ns*
MIR30A	microRNA 30a	2.8	4.5	*ns*
USP46	ubiquitin specific peptidase 46	3.0	4.5	*ns*
PPIP5K1	diphosphoinositol pentakisphosphate kinase 1	3.0	4.5	*ns*
LRP8	LDL receptor related protein 8	2.7	4.5	*ns*
GATM	glycine amidinotransferase (L-arginine:glycine amidinotransferase)	4.4	2.8	*ns*
SCNN1B	sodium channel, non voltage gated 1 beta subunit	4.0	4.4	*ns*
DYSF	dysferlin	4.1	4.4	*ns*
GSTA4	glutathione S-transferase alpha 4	2.5	4.4	*ns*
ANXA6	annexin A6	3.2	4.4	*ns*
SLC26A2	solute carrier family 26 (anion exchanger), member 2	2.9	4.4	*ns*
CA8	carbonic anhydrase VIII	4.3	3.3	*ns*
LINC01119	long intergenic non-protein coding RNA 1119	4.3	4.3	*ns*
PLAGL1	pleiomorphic adenoma gene-like 1	4.3	3.2	*ns*
GS1-594A7.3	uncharacterized LOC104798195	4.2	3.6	*ns*
CHN1	chimerin 1	4.2	3.6	*ns*
ADHFE1	alcohol dehydrogenase, iron containing 1	2.7	4.2	*ns*
MCOLN3	mucolipin 3	3.4	4.1	*ns*
PAPPA-AS2	PAPPA antisense RNA 2	3.0	4.1	*ns*
LOC101927354	uncharacterized LOC101927354	2.9	4.1	*ns*
KANK1	KN motif and ankyrin repeat domains 1	3.9	4.0	*ns*
IQGAP2	IQ motif containing GTPase activating protein 2	4.0	3.2	*ns*
MIR4659A	microRNA 4659a	4.0	4.0	*ns*
LINC00472	long intergenic non-protein coding RNA 472	4.0	2.4	*ns*
HSPB8	heat shock 22kDa protein 8	2.0	4.0	*ns*
ANKH	ANKH inorganic pyrophosphate transport regulator	4.0	2.4	*ns*
MIR30C2	microRNA 30c-2	3.4	4.0	*ns*
PLEKHG1	pleckstrin homology domain containing, family G (with RhoGef domain) member 1	4.0	2.4	*ns*
COLEC12	collectin sub-family member 12	4.0	3.9	*ns*
ELF5	E74-like factor 5 (ets domain transcription factor)	2.9	4.0	*ns*
ATP6V0A4	ATPase, H+ transporting, lysosomal V0 subunit a4	2.8	4.0	*ns*
FAM171B	family with sequence similarity 171, member B	3.3	4.0	*ns*
OR2F1	olfactory receptor, family 2, subfamily F, member 1 (gene	2.7	3.9	*ns*
GALNT11	polypeptide N-acetylgalactosaminyltransferase 11	3.3	3.9	*ns*
LOC105370312	uncharacterized LOC105370312	3.1	3.9	*ns*
CNTN5	contactin 5	3.9	3.9	*ns*
SYT7	synaptotagmin VII	2.1	3.9	*ns*
MIR3120	microRNA 3120	3.9	3.5	*ns*
CDH3	cadherin 3, type 1, P-cadherin (placental)	2.7	3.9	*ns*
LINC00597	long intergenic non-protein coding RNA 597	3.8	3.2	*ns*
MPP5	membrane protein, palmitoylated 5	2.7	3.8	*ns*
LOC105378239	uncharacterized LOC105378239	3.5	3.8	*ns*
ANKFN1	ankyrin-repeat and fibronectin type III domain containing 1	3.1	3.8	*ns*
TGFB3	transforming growth factor beta 3	3.3	3.8	*ns*
GRB14	growth factor receptor bound protein 14	3.8	3.3	*ns*
CARD18	caspase recruitment domain family, member 18	2.4	3.7	*ns*
SERINC5	serine incorporator 5	2.0	3.7	*ns*
ISL1	ISL LIM homeobox 1	2.8	3.7	*ns*
LOC101929325	uncharacterized LOC101929325	2.7	3.7	*ns*
CEP85	centrosomal protein 85kDa	2.2	3.7	*ns*
CLRN1-AS1	CLRN1 antisense RNA 1	2.2	3.6	*ns*
LRP6	LDL receptor related protein 6	3.6	2.3	*ns*
PLCXD2-AS1	PLCXD2 antisense RNA 1	2.6	3.6	*ns*
TMEM88	transmembrane protein 88	3.6	3.6	*ns*

Development • Supplementary information

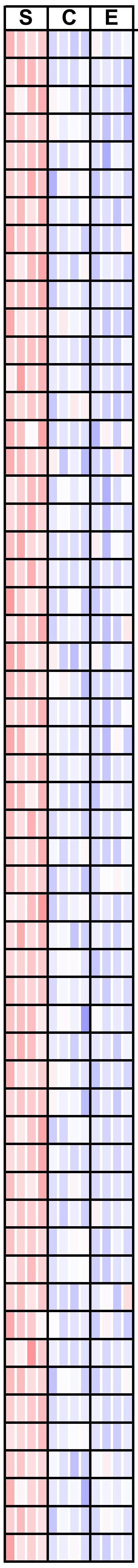

Symbol	Name	Linear Fold Change (*ns*=no significant difference) S vs C	S vs E	E vs C
INTS6L	integrator complex subunit 6 like	3.5	2.5	*ns*
FUT9	fucosyltransferase 9 (alpha (1,3) fucosyltransferase)	3.1	3.5	*ns*
ARL4C	ADP-ribosylation factor like GTPase 4C	2.4	3.5	*ns*
CDKL5	cyclin-dependent kinase-like 5	2.0	3.5	*ns*
SORBS1	sorbin and SH3 domain containing 1	2.8	3.5	*ns*
NCMAP	noncompact myelin associated protein	2.8	3.5	*ns*
CD99P1	CD99 molecule pseudogene 1	2.7	3.4	*ns*
PNP	purine nucleoside phosphorylase	3.4	3.2	*ns*
TFCP2L1	transcription factor CP2-like 1	3.1	3.4	*ns*
ARHGAP17	Rho GTPase activating protein 17	3.3	3.4	*ns*
CECR2	cat eye syndrome chromosome region, candidate 2	2.1	3.4	*ns*
GABRA3	gamma-aminobutyric acid (GABA) A receptor, alpha 3	3.2	3.3	*ns*
LOC105378032	uncharacterized LOC105378032	2.9	3.3	*ns*
PPP1R14C	protein phosphatase 1, regulatory (inhibitor) subunit 14C	2.2	3.3	*ns*
MAPK4	mitogen-activated protein kinase 4	3.3	3.1	*ns*
NOSTRIN	nitric oxide synthase trafficking	3.3	3.2	*ns*
C2CD3	C2 calcium-dependent domain containing 3	2.5	3.3	*ns*
AP1S3	adaptor-related protein complex 1 sigma 3 subunit	2.5	3.2	*ns*
PDE5A	phosphodiesterase 5A, cGMP-specific	3.2	2.6	*ns*
TMEM155	transmembrane protein 155	2.9	3.2	*ns*
LOC105369592	uncharacterized LOC105369592	3.2	3.0	*ns*
RALGAPA2	Ral GTPase activating protein, alpha subunit 2 (catalytic)	3.1	3.2	*ns*
FREM2	FRAS1 related extracellular matrix protein 2	3.2	2.8	*ns*
BTG3	BTG family, member 3	2.6	3.2	*ns*
ELMO1	engulfment and cell motility 1	2.7	3.2	*ns*
LOC105369683	uncharacterized LOC105369683	2.5	3.2	*ns*
NOS3	nitric oxide synthase 3 (endothelial cell)	2.6	3.2	*ns*
SCN11A	sodium channel, voltage gated, type XI alpha subunit	3.1	3.1	*ns*
PP12613	uncharacterized LOC100192379	3.1	3.0	*ns*
ST3GAL1	ST3 beta-galactoside alpha-2,3-sialyltransferase 1	2.4	3.1	*ns*
BMP2	bone morphogenetic protein 2	3.1	2.1	*ns*
FURIN	furin (paired basic amino acid cleaving enzyme)	2.7	3.1	*ns*
LOC105374693	spidroin-2-like	3.1	3.0	*ns*
AZIN1	antizyme inhibitor 1	2.1	3.1	*ns*
STARD4-AS1	STARD4 antisense RNA 1	2.6	3.1	*ns*
GCLM	glutamate-cysteine ligase, modifier subunit	3.1	2.6	*ns*
SLC16A12-AS1	SLC16A12 antisense RNA 1	3.1	3.0	*ns*
HOXA13	homeobox A13	2.1	3.1	*ns*
PHACTR2	phosphatase and actin regulator 2	2.5	3.0	*ns*
WWC1	WW and C2 domain containing 1	2.8	3.0	*ns*
NCBP3	nuclear cap binding subunit 3	2.8	3.0	*ns*
CTB-99A3.1	uncharacterized LOC105378215	2.6	3.0	*ns*
CHSY1	chondroitin sulfate synthase 1	3.0	2.3	*ns*
LOC102723465	uncharacterized LOC102723465	2.2	3.0	*ns*
LOC105369748	uncharacterized LOC105369748	2.1	2.9	*ns*
TMTC2	transmembrane and tetratricopeptide repeat containing 2	2.9	2.1	*ns*
GOLT1A	golgi transport 1A	2.6	2.9	*ns*
SLC4A4	solute carrier family 4 (sodium bicarbonate cotransporter), member 4	2.9	2.7	*ns*
LINC00882	long intergenic non-protein coding RNA 882	2.9	2.8	*ns*
SLC25A35	solute carrier family 25, member 35	2.7	2.9	*ns*
SCAPER	S-phase cyclin A-associated protein in the ER	2.0	2.9	*ns*
TSHZ1	teashirt zinc finger homeobox 1	2.9	2.0	*ns*
MIR4501	microRNA 4501	2.8	2.1	*ns*
LYPD8	LY6	2.8	2.3	*ns*
KDM7A-DT	KDM7A divergent transcript	2.4	2.8	*ns*

Development • Supplementary information

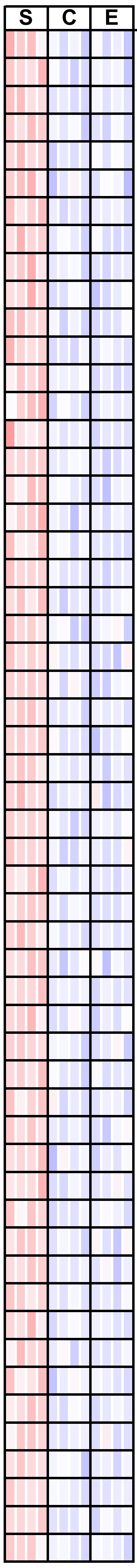

Symbol	Name	Linear Fold Change (*ns*=no significant difference) S vs C	S vs E	E vs C
CRNDE	colorectal neoplasia differentially expressed (non-protein coding)	2.8	2.8	*ns*
CADM1	cell adhesion molecule 1	2.8	2.6	*ns*
ATP2B1	ATPase, Ca++ transporting, plasma membrane 1	2.8	2.4	*ns*
CALHM4	calcium homeostasis modulator family member 4	2.6	2.8	*ns*
MYLK	myosin light chain kinase	2.8	2.2	*ns*
GPR171	G protein-coupled receptor 171	2.8	2.7	*ns*
SLC18B1	solute carrier family 18, subfamily B, member 1	2.8	2.5	*ns*
MIR199A2	microRNA 199a-2	2.6	2.7	*ns*
ETFDH	electron-transferring-flavoprotein dehydrogenase	2.5	2.7	*ns*
FAM234B	family with sequence similarity 234, member B	2.4	2.7	*ns*
PRTG	protogenin	2.7	2.7	*ns*
ABCC2	ATP binding cassette subfamily C member 2	2.7	2.5	*ns*
STAT5B	signal transducer and activator of transcription 5B	2.0	2.7	*ns*
PRKCE	protein kinase C, epsilon	2.7	2.4	*ns*
C16orf74	chromosome 16 open reading frame 74	2.2	2.7	*ns*
CAST	calpastatin	2.1	2.6	*ns*
ETV5	ets variant 5	2.2	2.6	*ns*
NRP2	neuropilin 2	2.6	2.4	*ns*
LOC105370632	uncharacterized LOC105370632	2.1	2.6	*ns*
PARP1	poly(ADP-ribose) polymerase 1	2.4	2.6	*ns*
RYR2	ryanodine receptor 2 (cardiac)	2.6	2.3	*ns*
WFDC1	WAP four-disulfide core domain 1	2.6	2.2	*ns*
MRPS31P5	mitochondrial ribosomal protein S31 pseudogene 5	2.1	2.6	*ns*
MAGEA10	MAGE family member A10	2.3	2.6	*ns*
AK3	adenylate kinase 3	2.5	2.4	*ns*
CTDSPL	CTD small phosphatase like	2.3	2.5	*ns*
CASP4	caspase 4	2.2	2.5	*ns*
PNPO	pyridoxamine 5-phosphate oxidase	2.5	2.5	*ns*
BHLHE41	basic helix-loop-helix family, member e41	2.5	2.0	*ns*
JCAD	junctional cadherin 5 associated	2.5	2.2	*ns*
PGAP6	post-glycosylphosphatidylinositol attachment to proteins 6	2.3	2.5	*ns*
CATSPER2	cation channel, sperm associated 2	2.2	2.5	*ns*
NREP	neuronal regeneration related protein	2.4	2.5	*ns*
DOCK8	dedicator of cytokinesis 8	2.5	2.4	*ns*
DGKD	diacylglycerol kinase, delta 130kDa	2.1	2.5	*ns*
EID2	EP300 interacting inhibitor of differentiation 2	2.3	2.4	*ns*
RARB	retinoic acid receptor, beta	2.3	2.4	*ns*
BMP1	bone morphogenetic protein 1	2.4	2.3	*ns*
NPAS2	neuronal PAS domain protein 2	2.3	2.4	*ns*
ZSCAN12P1	zinc finger and SCAN domain containing 12 pseudogene 1	2.4	2.4	*ns*
CDYL2	chromodomain protein, Y-like 2	2.4	2.4	*ns*
SPRY4	sprouty RTK signaling antagonist 4	2.4	2.1	*ns*
COQ9	coenzyme Q9	2.4	2.1	*ns*
MAGI3	membrane associated guanylate kinase, WW and PDZ domain containing 3	2.4	2.2	*ns*
ELK1	ELK1, member of ETS oncogene family	2.3	2.3	*ns*
LOC105375423	uncharacterized LOC105375423	2.3	2.3	*ns*
LOC101928092	uncharacterized LOC101928092	2.3	2.3	*ns*
MMP16	matrix metallopeptidase 16 (membrane-inserted)	2.2	2.3	*ns*
LOC105379091	uncharacterized LOC105379091	2.2	2.3	*ns*
VSIR	V-set immunoregulatory receptor	2.2	2.3	*ns*
KIAA0319	KIAA0319	2.2	2.2	*ns*
ORMDL1	ORMDL sphingolipid biosynthesis regulator 1	2.1	2.2	*ns*
PDE4D	phosphodiesterase 4D, cAMP-specific	2.1	2.2	*ns*
CDK6	cyclin-dependent kinase 6	2.2	2.1	*ns*
NETO2	neuropilin (NRP) and tolloid (TLL)-like 2	2.2	2.1	*ns*

Development • Supplementary information

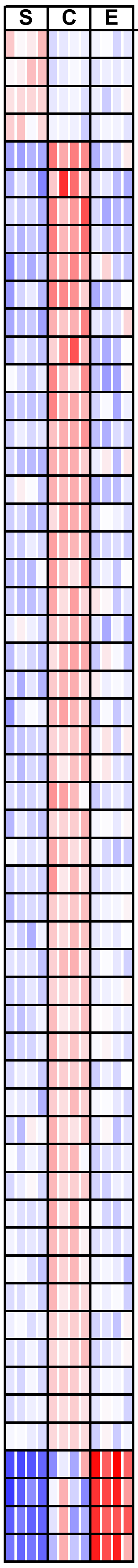

Symbol	Name	Linear Fold Change (*ns*=no significant difference) S vs C	S vs E	E vs C
NAV2-AS4	NAV2 antisense RNA 4	2.2	2.1	*ns*
ASB2	ankyrin repeat and SOCS box containing 2	2.1	2.1	*ns*
FNDC3A	fibronectin type III domain containing 3A	2.1	2.1	*ns*
LDHB	lactate dehydrogenase B	2.1	2.0	*ns*
OXGR1	oxoglutarate (alpha-ketoglutarate) receptor 1	-15.0	*ns*	*-7.4*
FABP7	fatty acid binding protein 7, brain	-12.6	*ns*	*-9.7*
LOC102723596	uncharacterized LOC102723596	-9.3	*ns*	*-11.5*
LOC105377976	uncharacterized LOC105377976	-10.3	*ns*	*-8.0*
TNNI2	troponin I type 2 (skeletal, fast)	-10.1	*ns*	*-4.6*
ITGB6	integrin beta 6	-8.3	*ns*	*-7.0*
COL17A1	collagen, type XVII, alpha 1	-8.2	*ns*	*-3.7*
SERPINB7	serpin peptidase inhibitor, clade B (ovalbumin), member 7	-7.1	*ns*	*-7.3*
RAB9BP1	RAB9B, member RAS oncogene family pseudogene 1	-4.4	*ns*	*-6.9*
NLRP2	NLR family, pyrin domain containing 2	-6.8	*ns*	*-5.3*
ACAN	aggrecan	-4.6	*ns*	*-6.2*
SERTAD4	SERTA domain containing 4	-5.9	*ns*	*-3.0*
FAM83B	family with sequence similarity 83, member B	-3.4	*ns*	*-5.7*
LOC102723854	uncharacterized LOC102723854	-5.2	*ns*	*-3.4*
VIT	vitrin	-4.7	*ns*	*-5.1*
KRT7	keratin 7, type II	-5.0	*ns*	*-3.7*
LOC105377408	uncharacterized LOC105377408	-4.8	*ns*	*-2.7*
TET1	tet methylcytosine dioxygenase 1	-2.9	*ns*	*-4.7*
KRT14	keratin 14, type I	-4.7	*ns*	*-3.5*
SRI	sorcin	-4.6	*ns*	*-2.3*
IGSF10	immunoglobulin superfamily, member 10	-4.2	*ns*	*-4.1*
CMTM4	CKLF-like MARVEL transmembrane domain containing 4	-4.2	*ns*	*-2.3*
SEMA4C	semaphorin 4C	-4.0	*ns*	*-2.3*
GDPD2	glycerophosphodiester phosphodiesterase domain containing 2	-4.0	*ns*	*-3.8*
EFNA1	ephrin-A1	-3.8	*ns*	*-2.1*
LINC00456	long intergenic non-protein coding RNA 456	-2.9	*ns*	*-3.7*
CCDC81	coiled-coil domain containing 81	-3.6	*ns*	*-2.7*
PKP2	plakophilin 2	-3.6	*ns*	*-2.1*
BMS1P18	BMS1 ribosome biogenesis factor pseudogene 18	-3.6	*ns*	*-2.4*
VLDLR	very low density lipoprotein receptor	-3.5	*ns*	*-2.9*
PTBP3	polypyrimidine tract binding protein 3	-3.2	*ns*	*-2.0*
GATA3	GATA binding protein 3	-3.2	*ns*	*-2.0*
APOBR	apolipoprotein B receptor	-3.1	*ns*	*-2.2*
PPARG	peroxisome proliferator-activated receptor gamma	-3.1	*ns*	*-2.9*
INHA	inhibin alpha	-3.0	*ns*	*-2.4*
DLX3	distal-less homeobox 3	-2.8	*ns*	*-3.0*
CTSV	cathepsin V	-2.6	*ns*	*-3.0*
NOCT	nocturnin	-2.6	*ns*	*-2.9*
LOC101927431	uncharacterized LOC101927431	-2.8	*ns*	*-2.9*
GPR37	G protein-coupled receptor 37 (endothelin receptor type B-like)	-2.7	*ns*	*-2.8*
ZNF257	zinc finger protein 257	-2.7	*ns*	*-2.8*
TRABD2A	TraB domain containing 2A	-2.8	*ns*	*-2.3*
LOC105379110	uncharacterized LOC105379110	-2.3	*ns*	*-2.7*
SYDE1	synapse defective 1, Rho GTPase, homolog 1 (C. elegans)	-2.7	*ns*	*-2.3*
RFK	riboflavin kinase	-2.6	*ns*	*-2.3*
RTN2	reticulon 2	-2.3	*ns*	*-2.1*
PIK3CB	phosphatidylinositol-4,5-bisphosphate 3-kinase, catalytic subunit beta	-2.0	*ns*	*-2.3*
SLPI	secretory leukocyte peptidase inhibitor	*ns*	*-157.1*	*24.7*
GZMA	granzyme A	*ns*	*-92.6*	*15.0*
CFH	complement factor H	*ns*	*-83.1*	*16.5*
TRDC	T cell receptor delta constant	*ns*	*-76.6*	*17.0*

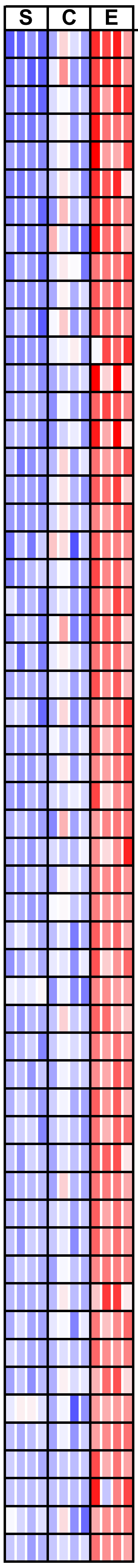

Symbol	Name	Linear Fold Change (*ns*=no significant difference) S vs C	S vs E	E vs C
TRDJ2	T cell receptor delta joining 2	*ns*	*-75.4*	*19.3*
OMD	osteomodulin	*ns*	*-64.4*	*14.7*
RXFP1	relaxin	*ns*	*-57.4*	*17.9*
C3	complement component 3	*ns*	*-44.8*	*15.5*
FGG	fibrinogen gamma chain	*ns*	*-35.1*	*18.2*
KLRC1	killer cell lectin-like receptor subfamily C, member 1	*ns*	*-34.8*	*14.2*
MEDAG	mesenteric estrogen-dependent adipogenesis	*ns*	*-34.7*	*12.1*
KLRC3	killer cell lectin-like receptor subfamily C, member 3	*ns*	*-33.6*	*16.2*
CXCL10	chemokine (C-X-C motif) ligand 10	*ns*	*-29.6*	*11.6*
CNR1	cannabinoid receptor 1 (brain)	*ns*	*-28.0*	*13.4*
DKK1	dickkopf WNT signaling pathway inhibitor 1	*ns*	*-27.5*	*9.5*
ANKRD1	ankyrin repeat domain 1 (cardiac muscle)	*ns*	*-26.4*	*8.9*
CP	ceruloplasmin (ferroxidase)	*ns*	*-24.8*	*15.9*
TRPC4	transient receptor potential cation channel, subfamily C, member 4	*ns*	*-24.1*	*23.2*
PI15	peptidase inhibitor 15	*ns*	*-23.3*	*18.2*
CYP7B1	cytochrome P450, family 7, subfamily B, polypeptide 1	*ns*	*-22.9*	*9.8*
NKG7	natural killer cell granule protein 7	*ns*	*-22.7*	*11.1*
NDP	Norrie disease (pseudoglioma)	*ns*	*-21.0*	*9.2*
APOD	apolipoprotein D	*ns*	*-20.3*	*8.9*
CPXM1	carboxypeptidase X (M14 family), member 1	*ns*	*-19.4*	*13.0*
CCL4	chemokine (C-C motif) ligand 4	*ns*	*-19.2*	*16.3*
PROK1	prokineticin 1	*ns*	*-19.1*	*9.9*
HSPB6	heat shock protein, alpha-crystallin-related, B6	*ns*	*-18.6*	*8.1*
TRDJ1	T cell receptor delta joining 1	*ns*	*-17.8*	*11.1*
ALDH1A1	aldehyde dehydrogenase 1 family, member A1	*ns*	*-17.7*	*12.9*
PRUNE2	prune homolog 2 (Drosophila)	*ns*	*-16.6*	*9.0*
TRBV3-1	T cell receptor beta variable 3-1	*ns*	*-16.0*	*10.4*
RGS1	regulator of G-protein signaling 1	*ns*	*-15.9*	*7.6*
CTSK	cathepsin K	*ns*	*-15.2*	*9.6*
RBP4	retinol binding protein 4, plasma	*ns*	*-14.8*	*8.9*
GZMK	granzyme K	*ns*	*-14.7*	*9.3*
MAOB	monoamine oxidase B	*ns*	*-14.5*	*6.7*
ABCA8	ATP binding cassette subfamily A member 8	*ns*	*-11.2*	*14.4*
LOC100507639	uncharacterized LOC100507639	*ns*	*-14.4*	*8.9*
PALMD	palmdelphin	*ns*	*-5.0*	*14.1*
MACC1-AS1	MACC1 antisense RNA 1	*ns*	*-13.9*	*6.8*
MAP3K5	mitogen-activated protein kinase kinase kinase 5	*ns*	*-13.9*	*7.6*
FPR3	formyl peptide receptor 3	*ns*	*-13.9*	*10.1*
KLRC4-KLRK1	KLRC4-KLRK1 readthrough	*ns*	*-13.9*	*11.4*
IFI44	interferon-induced protein 44	*ns*	*-13.5*	*12.3*
TRGJP1	T cell receptor gamma joining P1	*ns*	*-13.4*	*12.6*
PGR	progesterone receptor	*ns*	*-13.3*	*9.8*
SULF2	sulfatase 2	*ns*	*-13.1*	*10.5*
PARM1	prostate androgen-regulated mucin-like protein 1	*ns*	*-13.0*	*12.1*
TRGJP2	T cell receptor gamma joining P2	*ns*	*-13.0*	*11.4*
CST11	cystatin 11	*ns*	*-12.9*	*9.3*
ABI3BP	ABI family, member 3 (NESH) binding protein	*ns*	*-12.8*	*8.4*
AOX1	aldehyde oxidase 1	*ns*	*-12.7*	*11.5*
XCL2	chemokine (C motif) ligand 2	*ns*	*-12.6*	*6.5*
SPARCL1	SPARC like 1	*ns*	*-3.9*	*12.5*
HSD11B1	hydroxysteroid (11-beta) dehydrogenase 1	*ns*	*-11.8*	*12.4*
IL15	interleukin 15	*ns*	*-12.4*	*11.8*
FGA	fibrinogen alpha chain	*ns*	*-11.2*	*12.2*
COX7A1	cytochrome c oxidase subunit VIIa polypeptide 1 (muscle)	*ns*	*-7.1*	*12.1*
CD96	CD96 molecule	*ns*	*-12.1*	*9.0*

Development • Supplementary information

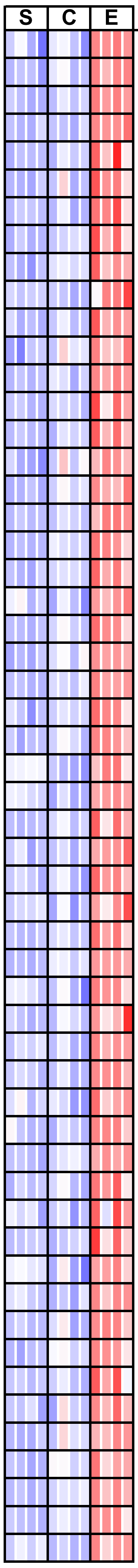

Symbol	Name	Linear Fold Change (*ns*=no significant difference) S vs C	S vs E	E vs C
SQOR	sulfide quinone oxidoreductase	*ns*	-12.1	9.5
PLPP1	phospholipid phosphatase 1	*ns*	-11.7	7.4
GALNT15	polypeptide N-acetylgalactosaminyltransferase 15	*ns*	-11.6	8.7
CLIC2	chloride intracellular channel 2	*ns*	-11.6	10.9
FHL5	four and a half LIM domains 5	*ns*	-11.4	8.7
GZMB	granzyme B	*ns*	-11.4	7.5
STAP1	signal transducing adaptor family member 1	*ns*	-10.9	11.2
AVPR1A	arginine vasopressin receptor 1A	*ns*	-11.1	7.5
WT1	Wilms tumor 1	*ns*	-11.0	5.6
LOC105372762	uncharacterized LOC105372762	*ns*	-8.5	10.9
PTPN22	protein tyrosine phosphatase, non-receptor type 22 (lymphoid)	*ns*	-10.8	8.8
CHI3L2	chitinase 3-like 2	*ns*	-10.6	4.4
IL2RG	interleukin 2 receptor, gamma	*ns*	-10.6	7.9
GPR174	G protein-coupled receptor 174	*ns*	-10.6	8.6
PDGFD	platelet derived growth factor D	*ns*	-10.5	8.3
MXRA7	matrix-remodelling associated 7	*ns*	-10.5	4.1
HLA-DPA1	major histocompatibility complex, class II, DP alpha 1	*ns*	-10.4	5.6
SULF1	sulfatase 1	*ns*	-10.3	8.5
PRF1	perforin 1 (pore forming protein)	*ns*	-10.1	6.2
ZNF727	zinc finger protein 727	*ns*	-10.1	6.9
FGF2	fibroblast growth factor 2 (basic)	*ns*	-5.9	10.0
PILRA	paired immunoglobin-like type 2 receptor alpha	*ns*	-10.0	6.7
CD74	CD74 molecule, major histocompatibility complex, class II invariant chain	*ns*	-10.0	5.0
ME1	malic enzyme 1, NADP(+)-dependent, cytosolic	*ns*	-10.0	6.6
GZMH	granzyme H	*ns*	-9.7	6.9
IGFBP2	insulin like growth factor binding protein 2	*ns*	-9.7	6.6
PLN	phospholamban	*ns*	-3.7	9.7
TM6SF1	transmembrane 6 superfamily member 1	*ns*	-5.4	9.6
CABCOCO1	ciliary associated calcium binding coiled-coil 1	*ns*	-9.5	6.5
TMTC1	transmembrane and tetratricopeptide repeat containing 1	*ns*	-9.5	9.2
PTPRC	protein tyrosine phosphatase, receptor type, C	*ns*	-9.3	9.4
SEMA3A	semaphorin 3A	*ns*	-6.4	9.3
SCARA5	scavenger receptor class A, member 5	*ns*	-9.3	7.0
SPOCK1	sparc	*ns*	-9.3	5.1
CLEC2B	C-type lectin domain family 2, member B	*ns*	-5.7	9.3
TRAV20	T cell receptor alpha variable 20	*ns*	-9.3	7.4
CFI	complement factor I	*ns*	-6.8	9.2
SYNPO2	synaptopodin 2	*ns*	-8.5	9.2
PLEKHH2	pleckstrin homology domain containing, family H (with MyTH4 domain) member 2	*ns*	-5.9	9.1
ABCA6	ATP binding cassette subfamily A member 6	*ns*	-7.7	9.1
PTHLH	parathyroid hormone-like hormone	*ns*	-9.1	5.6
CTSW	cathepsin W	*ns*	-9.0	7.7
SLC1A1	solute carrier family 1 (neuronal	*ns*	-7.0	9.0
FGB	fibrinogen beta chain	*ns*	-9.0	8.7
P2RY14	purinergic receptor P2Y, G-protein coupled, 14	*ns*	-4.1	8.9
PTPN13	protein tyrosine phosphatase, non-receptor type 13 (APO-1	*ns*	-6.6	8.9
IGFBP4	insulin like growth factor binding protein 4	*ns*	-8.8	7.3
IGFBP6	insulin like growth factor binding protein 6	*ns*	-8.8	4.4
GPR82	G protein-coupled receptor 82	*ns*	-8.7	8.2
RNASE2	ribonuclease, RNase A family, 2 (liver, eosinophil-derived neurotoxin)	*ns*	-8.6	6.9
PDZK1IP1	PDZK1 interacting protein 1	*ns*	-8.6	4.5
SERPINA3	serpin peptidase inhibitor, clade A (alpha-1 antiproteinase, antitrypsin), member 3	*ns*	-8.4	4.2
DEPTOR	DEP domain containing MTOR-interacting protein	*ns*	-8.4	5.1
PTPRCAP	protein tyrosine phosphatase, receptor type, C-associated protein	*ns*	-8.2	5.4
NAV3	neuron navigator 3	*ns*	-5.3	8.2

Development • Supplementary information

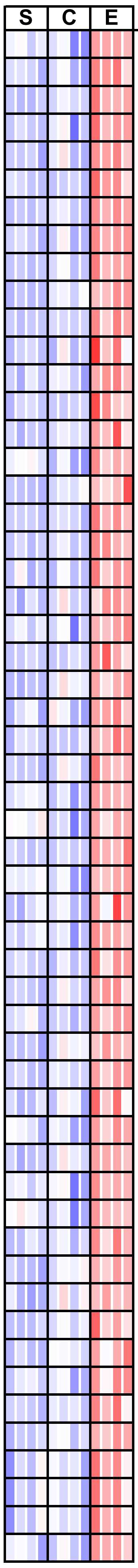

Symbol	Name	Linear Fold Change (*ns*=no significant difference) S vs C	S vs E	E vs C
PLCL1	phospholipase C-like 1	*ns*	-4.1	8.1
IGFBP7	insulin like growth factor binding protein 7	*ns*	-7.2	8.1
CD52	CD52 molecule	*ns*	-8.1	5.6
ADAMTS5	ADAM metallopeptidase with thrombospondin type 1 motif 5	*ns*	-7.9	8.0
GAS1	growth arrest-specific 1	*ns*	-8.0	5.8
LRRK2	leucine-rich repeat kinase 2	*ns*	-7.0	7.9
CCL4L2	chemokine (C-C motif) ligand 4-like 2	*ns*	-7.9	6.5
HAND2-AS1	HAND2 antisense RNA 1 (head to head)	*ns*	-7.8	7.6
PRDM1	PR domain containing 1, with ZNF domain	*ns*	-7.8	6.2
HAMP	hepcidin antimicrobial peptide	*ns*	-7.7	4.1
ENTPD3	ectonucleoside triphosphate diphosphohydrolase 3	*ns*	-7.7	6.4
REN	renin	*ns*	-7.7	6.8
MYL9	myosin light chain 9	*ns*	-7.5	6.6
CCL21	chemokine (C-C motif) ligand 21	*ns*	-7.5	7.2
LOC105379342	uncharacterized LOC105379342	*ns*	-7.4	5.0
ITGB8	integrin beta 8	*ns*	-3.2	7.4
HLA-DQA2	major histocompatibility complex, class II, DQ alpha 2	*ns*	-7.3	4.2
DOCK10	dedicator of cytokinesis 10	*ns*	-7.3	6.7
DTNA	dystrobrevin, alpha	*ns*	-7.3	6.1
CD226	CD226 molecule	*ns*	-6.7	7.2
ADRA2C	adrenoceptor alpha 2C	*ns*	-7.1	4.5
TLR4	toll-like receptor 4	*ns*	-4.9	7.1
TRDJ3	T cell receptor delta joining 3	*ns*	-7.1	6.6
PRR15	proline rich 15	*ns*	-7.0	3.0
ASPN	asporin	*ns*	-7.0	5.9
NPR3	natriuretic peptide receptor 3	*ns*	-6.9	7.0
CXCL13	chemokine (C-X-C motif) ligand 13	*ns*	-7.0	4.9
ZEB1	zinc finger E-box binding homeobox 1	*ns*	-3.9	7.0
TXNIP	thioredoxin interacting protein	*ns*	-2.6	7.0
TRGV10	T cell receptor gamma variable 10 (non-functional)	*ns*	-6.9	6.8
RGS2	regulator of G-protein signaling 2	*ns*	-3.4	6.9
TMEM47	transmembrane protein 47	*ns*	-6.9	6.8
EMILIN2	elastin microfibril interfacer 2	*ns*	-4.7	6.9
CD2	CD2 molecule	*ns*	-6.3	6.8
RGS22	regulator of G-protein signaling 22	*ns*	-6.8	5.1
DPYD	dihydropyrimidine dehydrogenase	*ns*	-5.0	6.8
OSGIN2	oxidative stress induced growth inhibitor family member 2	*ns*	-6.7	3.1
SAMD9L	sterile alpha motif domain containing 9-like	*ns*	-6.7	4.9
KIR2DL3	killer cell immunoglobulin-like receptor, two domains, long cytoplasmic tail, 3	*ns*	-6.6	5.6
RNASE6	ribonuclease, RNase A family, k6	*ns*	-4.7	6.6
CXCL16	chemokine (C-X-C motif) ligand 16	*ns*	-6.6	3.8
CD248	CD248 molecule, endosialin	*ns*	-4.3	6.6
PDGFRA	platelet-derived growth factor receptor, alpha polypeptide	*ns*	-2.9	6.6
KCND2	potassium channel, voltage gated Shal related subfamily D, member 2	*ns*	-6.5	5.6
ANG	angiogenin, ribonuclease, RNase A family, 5	*ns*	-6.5	3.1
METRN	meteorin, glial cell differentiation regulator	*ns*	-6.5	2.9
GLB1L2	galactosidase beta 1 like 2	*ns*	-6.5	4.1
GPR155	G protein-coupled receptor 155	*ns*	-6.5	3.5
OGN	osteoglycin	*ns*	-5.3	6.4
KLRB1	killer cell lectin-like receptor subfamily B, member 1	*ns*	-6.4	5.0
EMP3	epithelial membrane protein 3	*ns*	-6.3	4.3
LOC102724994	putative POM121-like protein 1-like	*ns*	-6.3	5.5
LOC441081	POM121 membrane glycoprotein (rat) pseudogene	*ns*	-6.3	5.5
LOC728093	putative POM121-like protein 1-like	*ns*	-6.3	5.5
CTSS	cathepsin S	*ns*	-5.1	6.3

Development • Supplementary information

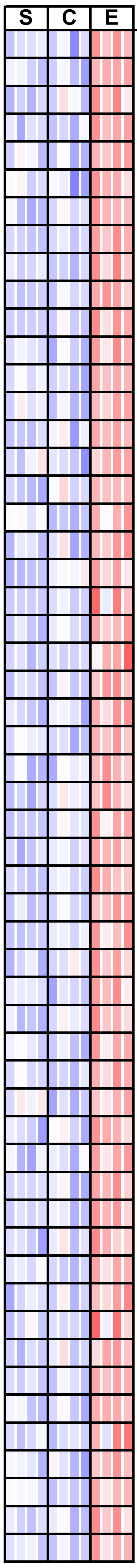

Symbol	Name	Linear Fold Change (*ns*=no significant difference) S vs C	S vs E	E vs C
LAPTM5	lysosomal protein transmembrane 5	*ns*	-4.9	6.3
MS4A6A	membrane-spanning 4-domains, subfamily A, member 6A	*ns*	-4.7	6.2
TUBA3E	tubulin, alpha 3e	*ns*	-6.2	3.9
SCPEP1	serine carboxypeptidase 1	*ns*	-5.8	6.1
FGL2	fibrinogen-like 2	*ns*	-4.2	6.1
CCL2	chemokine (C-C motif) ligand 2	*ns*	-3.5	6.1
CERS6-AS1	CERS6 antisense RNA 1	*ns*	-6.1	4.1
GREB1	growth regulation by estrogen in breast cancer 1	*ns*	-6.1	5.3
APOBEC3C	apolipoprotein B mRNA editing enzyme, catalytic polypeptide-like 3C	*ns*	-4.5	6.0
KLF9	Kruppel-like factor 9	*ns*	-6.0	4.9
EVA1C	eva-1 homolog C (C. elegans)	*ns*	-5.9	5.2
STAMBPL1	STAM binding protein-like 1	*ns*	-4.1	5.9
MCTP1	multiple C2 domains, transmembrane 1	*ns*	-3.7	5.9
MS4A7	membrane-spanning 4-domains, subfamily A, member 7	*ns*	-3.5	5.9
ISLR	immunoglobulin superfamily containing leucine-rich repeat	*ns*	-5.8	4.1
THBS1	thrombospondin 1	*ns*	-3.6	5.8
RBP1	retinol binding protein 1, cellular	*ns*	-5.8	2.9
MECOM	MDS1 and EVI1 complex locus	*ns*	-2.6	5.7
MGP	matrix Gla protein	*ns*	-5.7	5.4
DHRS3	dehydrogenase	*ns*	-5.7	2.4
KERA	keratocan	*ns*	-5.7	4.8
HTR2B	5-hydroxytryptamine (serotonin) receptor 2B, G protein-coupled	*ns*	-5.7	4.1
TNFRSF11B	tumor necrosis factor receptor superfamily, member 11b	*ns*	-5.7	5.2
LOC105376125	uncharacterized LOC105376125	*ns*	-5.7	5.1
CAB39L	calcium binding protein 39-like	*ns*	-5.7	4.8
FCER1G	Fc fragment of IgE, high affinity I, receptor for gamma polypeptide	*ns*	-3.5	5.6
LYZ	lysozyme	*ns*	-5.5	4.1
CST7	cystatin F (leukocystatin)	*ns*	-5.5	3.0
MGST1	microsomal glutathione S-transferase 1	*ns*	-5.5	3.6
MFGE8	milk fat globule-EGF factor 8 protein	*ns*	-5.5	3.8
HAND2	heart and neural crest derivatives expressed 2	*ns*	-5.5	4.2
S100A3	S100 calcium binding protein A3	*ns*	-5.5	4.6
CD38	CD38 molecule	*ns*	-5.5	3.6
MACC1	metastasis associated in colon cancer 1	*ns*	-5.5	3.7
LMOD1	leiomodin 1 (smooth muscle)	*ns*	-3.6	5.5
CEP126	centrosomal protein 126kDa	*ns*	-5.4	3.3
CD53	CD53 molecule	*ns*	-3.6	5.4
MAP1LC3C	microtubule associated protein 1 light chain 3 gamma	*ns*	-4.1	5.4
BTBD3	BTB (POZ) domain containing 3	*ns*	-2.4	5.3
TLR1	toll-like receptor 1	*ns*	-5.3	4.1
CERS6	ceramide synthase 6	*ns*	-5.3	4.4
TYROBP	TYRO protein tyrosine kinase binding protein	*ns*	-4.7	5.3
AXL	AXL receptor tyrosine kinase	*ns*	-5.0	5.2
CCDC170	coiled-coil domain containing 170	*ns*	-5.2	3.5
LCP2	lymphocyte cytosolic protein 2	*ns*	-3.5	5.2
LINC00707	long intergenic non-protein coding RNA 707	*ns*	-5.2	4.8
TSPAN1	tetraspanin 1	*ns*	-4.9	5.2
LOC389033	placenta-specific 9 pseudogene	*ns*	-5.1	3.5
APOO	apolipoprotein O	*ns*	-5.0	5.1
OTULINL	OTU deubiquitinase with linear linkage specificity like	*ns*	-4.6	5.0
APCS	amyloid P component, serum	*ns*	-4.5	5.0
TLR7	toll-like receptor 7	*ns*	-3.9	4.9
MYO5A	myosin VA	*ns*	-2.8	4.9
C1QC	complement component 1, q subcomponent, C chain	*ns*	-4.7	4.8
GTF2A1L	general transcription factor IIA 1-like	*ns*	-3.9	4.8

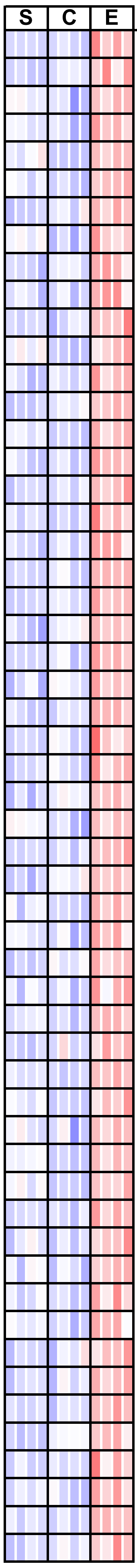

Symbol	Name	S vs C	S vs E	E vs C
		Linear Fold Change (*ns*=no significant difference)		
PRPS2	phosphoribosyl pyrophosphate synthetase 2	*ns*	*-4.7*	*4.8*
NCAM1	neural cell adhesion molecule 1	*ns*	*-4.8*	*3.5*
SH3BGRL	SH3 domain binding glutamate-rich protein like	*ns*	*-2.5*	*4.8*
C1QA	complement component 1, q subcomponent, A chain	*ns*	*-3.1*	*4.8*
MSN	moesin	*ns*	*-2.5*	*4.8*
ODC1	ornithine decarboxylase 1	*ns*	*-3.1*	*4.8*
CD68	CD68 molecule	*ns*	*-4.8*	*3.0*
LONRF2	LON peptidase N-terminal domain and ring finger 2	*ns*	*-2.3*	*4.8*
SETDB2	SET domain, bifurcated 2	*ns*	*-4.8*	*3.3*
HOXA11	homeobox A11	*ns*	*-4.5*	*4.7*
GBP5	guanylate binding protein 5	*ns*	*-4.6*	*4.7*
ICAM2	intercellular adhesion molecule 2	*ns*	*-2.2*	*4.7*
C1GALT1C1L	C1GALT1-specific chaperone 1 like	*ns*	*-4.7*	*3.8*
TMEM132C	transmembrane protein 132C	*ns*	*-4.7*	*2.9*
SYTL4	synaptotagmin-like 4	*ns*	*-3.0*	*4.6*
C1S	complement component 1, s subcomponent	*ns*	*-4.5*	*4.6*
GPRIN3	GPRIN family member 3	*ns*	*-4.6*	*3.8*
EOMES	eomesodermin	*ns*	*-4.0*	*4.6*
C5AR1	complement component 5a receptor 1	*ns*	*-4.6*	*4.0*
C1QB	complement component 1, q subcomponent, B chain	*ns*	*-4.6*	*4.1*
C1R	complement component 1, r subcomponent	*ns*	*-4.6*	*3.6*
MTDH	metadherin	*ns*	*-4.5*	*2.4*
NNMT	nicotinamide N-methyltransferase	*ns*	*-4.5*	*4.2*
PLA2R1	phospholipase A2 receptor 1	*ns*	*-4.5*	*2.8*
EMCN	endomucin	*ns*	*-4.5*	*3.9*
CCN4	cellular communication network factor 4	*ns*	*-4.5*	*3.6*
TLR2	toll-like receptor 2	*ns*	*-4.5*	*4.0*
TIPARP	TCDD-inducible poly(ADP-ribose) polymerase	*ns*	*-4.5*	*2.5*
MAP1B	microtubule associated protein 1B	*ns*	*-2.1*	*4.5*
CD3D	CD3d molecule, delta (CD3-TCR complex)	*ns*	*-4.5*	*4.3*
FOXO1	forkhead box O1	*ns*	*-4.4*	*2.3*
HCST	hematopoietic cell signal transducer	*ns*	*-3.2*	*4.4*
PRKACB	protein kinase, cAMP-dependent, catalytic, beta	*ns*	*-3.0*	*4.4*
ANOS1	anosmin 1	*ns*	*-4.4*	*2.6*
LOC105369863	uncharacterized LOC105369863	*ns*	*-3.7*	*4.4*
DDR2	discoidin domain receptor tyrosine kinase 2	*ns*	*-3.3*	*4.4*
LOC101926934	uncharacterized LOC101926934	*ns*	*-4.4*	*3.2*
CCL23	chemokine (C-C motif) ligand 23	*ns*	*-3.4*	*4.4*
VWF	von Willebrand factor	*ns*	*-2.7*	*4.4*
LOC101927414	uncharacterized LOC101927414	*ns*	*-2.8*	*4.4*
IFI16	interferon, gamma-inducible protein 16	*ns*	*-2.4*	*4.4*
EMP1	epithelial membrane protein 1	*ns*	*-2.3*	*4.4*
FBLN5	fibulin 5	*ns*	*-4.3*	*4.4*
MIR4263	microRNA 4263	*ns*	*-3.5*	*4.3*
PCBD1	pterin-4 alpha-carbinolamine dehydratase	*ns*	*-3.0*	*4.3*
STK39	serine threonine kinase 39	*ns*	*-3.4*	*4.3*
OLFML2B	olfactomedin like 2B	*ns*	*-2.6*	*4.3*
RNF144B	ring finger protein 144B	*ns*	*-4.3*	*3.1*
RGCC	regulator of cell cycle	*ns*	*-4.2*	*3.8*
LSAMP	limbic system-associated membrane protein	*ns*	*-4.1*	*4.2*
LOC100505851	uncharacterized LOC100505851	*ns*	*-4.2*	*2.2*
DPYD-AS1	DPYD antisense RNA 1	*ns*	*-4.2*	*3.8*
JUN	jun proto-oncogene	*ns*	*-3.4*	*4.2*
CCL4L1	chemokine (C-C motif) ligand 4-like 1	*ns*	*-3.7*	*4.2*
COPZ2	coatomer protein complex subunit zeta 2	*ns*	*-4.2*	*3.0*

Development • Supplementary information

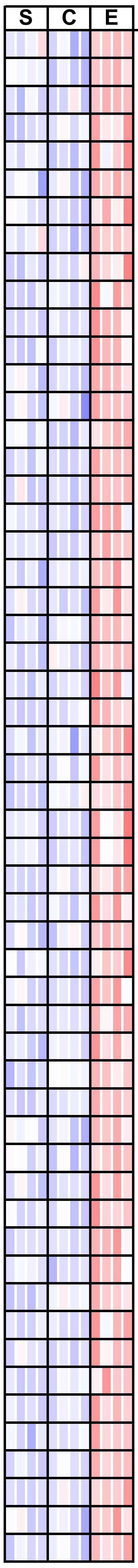

Symbol	Name	Linear Fold Change (*ns*=no significant difference) S vs C	S vs E	E vs C
IFITM2	interferon induced transmembrane protein 2	*ns*	-2.4	4.2
LPAR1	lysophosphatidic acid receptor 1	*ns*	-2.4	4.2
IL13RA2	interleukin 13 receptor, alpha 2	*ns*	-4.2	3.9
ALDH1A2	aldehyde dehydrogenase 1 family, member A2	*ns*	-4.2	2.9
WNT5A	wingless-type MMTV integration site family, member 5A	*ns*	-3.1	4.2
TPST1	tyrosylprotein sulfotransferase 1	*ns*	-3.9	4.1
ST6GALNAC3	ST6 (alpha-N-acetyl-neuraminyl-2,3-beta-galactosyl-1,3)-N-acetylgalactosaminide alpha-2,6-sialyltransferase 3	*ns*	-2.9	4.1
PDGFRB	platelet-derived growth factor receptor, beta polypeptide	*ns*	-2.3	4.1
CXCL11	chemokine (C-X-C motif) ligand 11	*ns*	-4.1	3.0
PTGER4	prostaglandin E receptor 4 (subtype EP4)	*ns*	-4.1	3.3
ARL4D	ADP-ribosylation factor like GTPase 4D	*ns*	-4.1	3.3
PTGDR	prostaglandin D2 receptor (DP)	*ns*	-4.1	3.6
GNPDA2	glucosamine-6-phosphate deaminase 2	*ns*	-3.2	4.1
FAP	fibroblast activation protein alpha	*ns*	-3.7	4.1
ZNF667-AS1	ZNF667 antisense RNA 1 (head to head)	*ns*	-2.8	4.1
APCDD1	adenomatosis polyposis coli down-regulated 1	*ns*	-3.9	4.1
FYN	FYN proto-oncogene, Src family tyrosine kinase	*ns*	-3.7	4.1
CFD	complement factor D (adipsin)	*ns*	-3.5	4.0
ARMC9	armadillo repeat containing 9	*ns*	-3.3	4.0
RRAS	related RAS viral (r-ras) oncogene homolog	*ns*	-4.0	3.0
ARHGAP15	Rho GTPase activating protein 15	*ns*	-3.2	4.0
MSR1	macrophage scavenger receptor 1	*ns*	-4.0	2.9
INPP4B	inositol polyphosphate-4-phosphatase type II B	*ns*	-3.9	3.1
KIR2DL4	killer cell immunoglobulin-like receptor, two domains, long cytoplasmic tail, 4	*ns*	-3.9	3.8
CXCL12	chemokine (C-X-C motif) ligand 12	*ns*	-3.9	3.0
XGY2	Xg pseudogene, Y-linked 2	*ns*	-3.7	3.9
CILP	cartilage intermediate layer protein, nucleotide pyrophosphohydrolase	*ns*	-3.9	3.1
SLC25A30	solute carrier family 25, member 30	*ns*	-3.9	2.3
TRGC2	T cell receptor gamma constant 2	*ns*	-3.2	3.9
TRGV9	T cell receptor gamma variable 9	*ns*	-3.2	3.9
TIMP4	TIMP metallopeptidase inhibitor 4	*ns*	-3.9	2.9
CCL3L3	chemokine (C-C motif) ligand 3-like 3	*ns*	-3.9	3.4
IL18	interleukin 18	*ns*	-3.9	3.4
PLA1A	phospholipase A1 member A	*ns*	-2.8	3.9
PIK3CG	phosphatidylinositol-4,5-bisphosphate 3-kinase, catalytic subunit gamma	*ns*	-3.3	3.9
FAM155A	family with sequence similarity 155, member A	*ns*	-3.8	3.4
BIRC3	baculoviral IAP repeat containing 3	*ns*	-3.8	3.0
APBB1IP	amyloid beta (A4) precursor protein-binding, family B, member 1 interacting protein	*ns*	-3.8	2.1
SKAP1	src kinase associated phosphoprotein 1	*ns*	-3.8	3.2
CD58	CD58 molecule	*ns*	-2.6	3.8
FAM229B	family with sequence similarity 229, member B	*ns*	-2.7	3.8
CFAP300	cilia and flagella associated protein 300	*ns*	-3.7	3.8
AOAH	acyloxyacyl hydrolase (neutrophil)	*ns*	-3.2	3.8
CLECL1	C-type lectin-like 1	*ns*	-3.8	3.6
CD209	CD209 molecule	*ns*	-2.8	3.8
GADD45A	growth arrest and DNA-damage-inducible, alpha	*ns*	-3.8	2.1
SIPA1L2	signal-induced proliferation-associated 1 like 2	*ns*	-3.7	3.8
IL1B	interleukin 1 beta	*ns*	-3.1	3.8
P2RY10	purinergic receptor P2Y, G-protein coupled, 10	*ns*	-3.8	2.8
ZNF804A	zinc finger protein 804A	*ns*	-3.7	3.4
PYHIN1	pyrin and HIN domain family, member 1	*ns*	-3.7	3.1
NIPAL2	NIPA-like domain containing 2	*ns*	-3.7	3.2
LOC102723721	uncharacterized LOC102723721	*ns*	-3.7	3.1
GLIDR	glioblastoma down-regulated RNA	*ns*	-3.0	3.7
PAMR1	peptidase domain containing associated with muscle regeneration 1	*ns*	-3.7	3.4

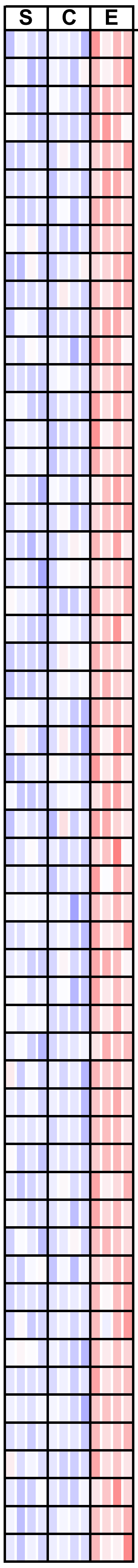

Symbol	Name	Linear Fold Change (*ns*=no significant difference) S vs C	S vs E	E vs C
LOC101928461	uncharacterized LOC101928461	*ns*	-3.7	3.4
IL18R1	interleukin 18 receptor 1	*ns*	-3.7	2.8
MNDA	myeloid cell nuclear differentiation antigen	*ns*	-3.7	3.2
SNORA78	small nucleolar RNA, H	*ns*	-3.4	3.6
UCHL1	ubiquitin C-terminal hydrolase L1	*ns*	-3.6	3.1
TPM2	tropomyosin 2 (beta)	*ns*	-3.2	3.6
CD48	CD48 molecule	*ns*	-3.6	3.5
ZNF25	zinc finger protein 25	*ns*	-2.8	3.6
CDA	cytidine deaminase	*ns*	-3.6	2.9
KCNH1	potassium channel, voltage gated eag related subfamily H, member 1	*ns*	-3.6	3.0
PWWP3B	PWWP domain containing 3B	*ns*	-3.4	3.5
NPR1	natriuretic peptide receptor 1	*ns*	-2.7	3.5
SOD2	superoxide dismutase 2, mitochondrial	*ns*	-3.5	2.7
TASL	TLR adaptor interacting with endolysosomal SLC15A4	*ns*	-3.3	3.5
TAGAP	T-cell activation RhoGTPase activating protein	*ns*	-2.8	3.5
DOCK2	dedicator of cytokinesis 2	*ns*	-2.6	3.5
SEMA3B-AS1	SEMA3B antisense RNA 1 (head to head)	*ns*	-3.5	2.9
MPZL2	myelin protein zero-like 2	*ns*	-3.3	3.5
RASD1	RAS, dexamethasone-induced 1	*ns*	-3.5	2.6
PRPS1	phosphoribosyl pyrophosphate synthetase 1	*ns*	-3.5	2.3
MDM1	Mdm1 nuclear protein	*ns*	-2.7	3.5
OXT	oxytocin	*ns*	-3.5	2.9
BRINP2	bone morphogenetic protein	*ns*	-3.5	2.2
TNC	tenascin C	*ns*	-3.5	2.3
C8orf88	chromosome 8 open reading frame 88	*ns*	-2.6	3.5
SLC46A3	solute carrier family 46, member 3	*ns*	-3.2	3.4
EMX2	empty spiracles homeobox 2	*ns*	-3.4	2.8
DNLZ	DNL-type zinc finger	*ns*	-3.4	2.7
ID4	inhibitor of DNA binding 4, dominant negative helix-loop-helix protein	*ns*	-3.4	3.1
TAGLN	transgelin	*ns*	-3.1	3.4
SORL1	sortilin-related receptor, L(DLR class) A repeats containing	*ns*	-2.8	3.4
FOLR2	folate receptor 2 (fetal)	*ns*	-2.2	3.4
POLR1F	RNA polymerase I subunit F	*ns*	-2.9	3.4
TMSB4X	thymosin beta 4, X-linked	*ns*	-3.4	3.3
TRPS1	trichorhinophalangeal syndrome I	*ns*	-2.4	3.4
ZBTB20	zinc finger and BTB domain containing 20	*ns*	-2.2	3.4
WDR41	WD repeat domain 41	*ns*	-3.4	2.9
LOC101928100	uncharacterized LOC101928100	*ns*	-2.7	3.3
TP53I3	tumor protein p53 inducible protein 3	*ns*	-3.0	3.3
ADA2	adenosine deaminase 2	*ns*	-2.7	3.3
QPCT	glutaminyl-peptide cyclotransferase	*ns*	-3.3	3.2
PLAT	plasminogen activator, tissue	*ns*	-3.3	2.7
TNFSF13B	tumor necrosis factor (ligand) superfamily, member 13b	*ns*	-3.0	3.3
SPAG1	sperm associated antigen 1	*ns*	-3.3	2.9
ABLIM3	actin binding LIM protein family, member 3	*ns*	-2.7	3.2
LINC00924	long intergenic non-protein coding RNA 924	*ns*	-3.2	2.8
GSTM5	glutathione S-transferase mu 5	*ns*	-3.2	3.0
ALCAM	activated leukocyte cell adhesion molecule	*ns*	-2.1	3.2
NCOA7	nuclear receptor coactivator 7	*ns*	-3.2	3.2
LCP1	lymphocyte cytosolic protein 1 (L-plastin)	*ns*	-2.5	3.2
PRSS23	protease, serine, 23	*ns*	-3.2	3.1
ACSL1	acyl-CoA synthetase long-chain family member 1	*ns*	-2.3	3.2
SMIM10	small integral membrane protein 10	*ns*	-3.2	3.2
LSAMP-AS1	uncharacterized LOC101926903	*ns*	-3.2	2.6
SNCAIP	synuclein alpha interacting protein	*ns*	-3.2	2.9

Development • Supplementary information

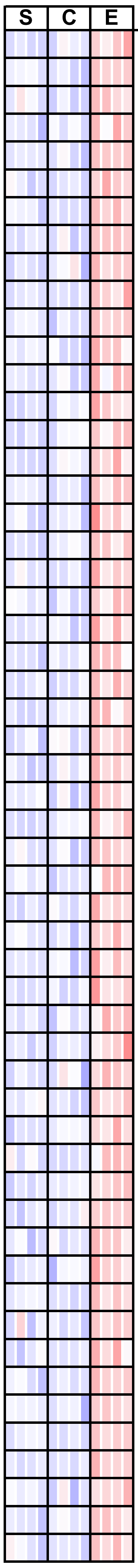

Symbol	Name	Linear Fold Change (*ns*=no significant difference) S vs C	S vs E	E vs C
PLD1	phospholipase D1, phosphatidylcholine-specific	*ns*	-3.2	2.6
MCC	mutated in colorectal cancers	*ns*	-2.4	3.2
ARMCX1	armadillo repeat containing, X-linked 1	*ns*	-2.2	3.2
CLEC2D	C-type lectin domain family 2, member D	*ns*	-3.2	2.6
LGALS1	lectin, galactoside-binding, soluble, 1	*ns*	-2.7	3.2
UG0898H09	uncharacterized LOC643763	*ns*	-2.9	3.2
SAMHD1	SAM domain and HD domain 1	*ns*	-2.7	3.2
HLA-DMB	major histocompatibility complex, class II, DM beta	*ns*	-3.0	3.2
MANEA	mannosidase, endo-alpha	*ns*	-3.1	2.5
JAKMIP2	janus kinase and microtubule interacting protein 2	*ns*	-3.0	3.1
MEF2A	myocyte enhancer factor 2A	*ns*	-2.5	3.1
C2CD4B	C2 calcium-dependent domain containing 4B	*ns*	-2.4	3.1
MIR99AHG	mir-99a-let-7c cluster host gene	*ns*	-2.9	3.1
HLA-DPB1	major histocompatibility complex, class II, DP beta 1	*ns*	-3.1	2.4
WNT4	wingless-type MMTV integration site family, member 4	*ns*	-3.1	2.2
C1RL	complement component 1, r subcomponent-like	*ns*	-3.1	2.2
PRXL2A	peroxiredoxin like 2A	*ns*	-2.5	3.1
EVI2B	ecotropic viral integration site 2B	*ns*	-3.0	3.1
RCBTB1	regulator of chromosome condensation (RCC1) and BTB (POZ) domain containing protein 1	*ns*	-3.1	2.3
KATNAL1	katanin p60 subunit A-like 1	*ns*	-2.5	3.1
MID2	midline 2	*ns*	-2.2	3.1
ADAMTS2	ADAM metallopeptidase with thrombospondin type 1 motif 2	*ns*	-2.9	3.0
INE2	inactivation escape 2 (non-protein coding)	*ns*	-3.0	2.3
KCNK6	potassium channel, two pore domain subfamily K, member 6	*ns*	-2.8	3.0
GALNT13	polypeptide N-acetylgalactosaminyltransferase 13	*ns*	-3.0	2.4
PTGDS	prostaglandin D2 synthase 21kDa (brain)	*ns*	-3.0	2.3
RAI2	retinoic acid induced 2	*ns*	-3.0	2.3
SIGLEC7	sialic acid binding Ig-like lectin 7	*ns*	-2.3	3.0
EDNRB	endothelin receptor type B	*ns*	-3.0	2.2
MSANTD4	Myb	*ns*	-2.5	3.0
CLEC14A	C-type lectin domain family 14, member A	*ns*	-2.4	3.0
FBLN2	fibulin 2	*ns*	-3.0	2.4
S100A16	S100 calcium binding protein A16	*ns*	-2.3	3.0
CYBRD1	cytochrome b reductase 1	*ns*	-2.8	3.0
FAM155A-IT1	FAM155A intronic transcript 1	*ns*	-3.0	2.8
CORIN	corin, serine peptidase	*ns*	-2.9	3.0
MLLT11	myeloid	*ns*	-3.0	2.9
TMEM220	transmembrane protein 220	*ns*	-3.0	2.7
RGL1	ral guanine nucleotide dissociation stimulator-like 1	*ns*	-2.1	3.0
SAMD3	sterile alpha motif domain containing 3	*ns*	-3.0	2.1
EPHX4	epoxide hydrolase 4	*ns*	-2.3	3.0
DNASE1L3	deoxyribonuclease I-like 3	*ns*	-3.0	2.2
NFE2L1	nuclear factor, erythroid 2-like 1	*ns*	-2.9	2.4
KIR2DS3	killer cell immunoglobulin-like receptor, two domains, short cytoplasmic tail, 3	*ns*	-2.9	2.5
ITGAX	integrin alpha X	*ns*	-2.9	2.8
HAVCR2	hepatitis A virus cellular receptor 2	*ns*	-2.2	2.9
CRYZ	crystallin zeta	*ns*	-2.4	2.9
NR1D1	nuclear receptor subfamily 1, group D, member 1	*ns*	-2.9	2.8
TTC30A	tetratricopeptide repeat domain 30A	*ns*	-2.9	2.9
EOGT	EGF domain-specific O-linked N-acetylglucosamine (GlcNAc) transferase	*ns*	-2.1	2.9
KALRN	kalirin, RhoGEF kinase	*ns*	-2.3	2.8
SMOX	spermine oxidase	*ns*	-2.8	2.8
OSBPL1A	oxysterol binding protein-like 1A	*ns*	-2.2	2.8
CFL2	cofilin 2 (muscle)	*ns*	-2.8	2.3
DNAAF4	dynein axonemal assembly factor 4	*ns*	-2.4	2.8

Development • Supplementary information

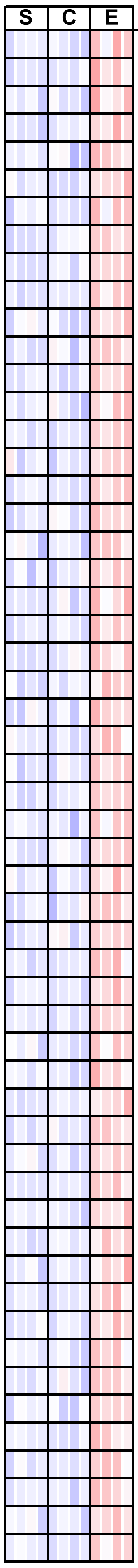

Symbol	Name	Linear Fold Change (*ns*=no significant difference) S vs C	S vs E	E vs C
RNF138P1	ring finger protein 138, E3 ubiquitin protein ligase pseudogene 1	*ns*	-2.5	2.8
IRS2	insulin receptor substrate 2	*ns*	-2.8	2.4
LOC105376272	uncharacterized LOC105376272	*ns*	-2.6	2.8
ARL3	ADP-ribosylation factor like GTPase 3	*ns*	-2.8	2.3
CCL3	chemokine (C-C motif) ligand 3	*ns*	-2.3	2.8
S1PR4	sphingosine-1-phosphate receptor 4	*ns*	-2.2	2.8
FAM110C	family with sequence similarity 110, member C	*ns*	-2.4	2.8
HLA-DMA	major histocompatibility complex, class II, DM alpha	*ns*	-2.7	2.0
NIBAN1	niban apoptosis regulator 1	*ns*	-2.7	2.2
LOC100507195	uncharacterized LOC100507195	*ns*	-2.7	2.4
ZNF287	zinc finger protein 287	*ns*	-2.2	2.7
NUPR1	nuclear protein 1, transcriptional regulator	*ns*	-2.7	2.3
ATP2B4	ATPase, Ca++ transporting, plasma membrane 4	*ns*	-2.5	2.7
TMEM98	transmembrane protein 98	*ns*	-2.3	2.7
TMEM35B	transmembrane protein 35B	*ns*	-2.5	2.7
C15orf65	chromosome 15 open reading frame 65	*ns*	-2.1	2.7
CELF2	CUGBP, Elav-like family member 2	*ns*	-2.5	2.7
CERK	ceramide kinase	*ns*	-2.7	2.2
TLR6	toll-like receptor 6	*ns*	-2.5	2.7
SLFN12	schlafen family member 12	*ns*	-2.7	2.4
GJA1	gap junction protein alpha 1	*ns*	-2.7	2.6
IL2RA	interleukin 2 receptor, alpha	*ns*	-2.7	2.6
ZNF438	zinc finger protein 438	*ns*	-2.6	2.2
HLA-A	major histocompatibility complex, class I, A	*ns*	-2.6	2.2
SOD3	superoxide dismutase 3, extracellular	*ns*	-2.6	2.6
CHST2	carbohydrate (N-acetylglucosamine-6-O) sulfotransferase 2	*ns*	-2.1	2.6
ZNF516	zinc finger protein 516	*ns*	-2.6	2.5
RBKS	ribokinase	*ns*	-2.6	2.3
C1orf162	chromosome 1 open reading frame 162	*ns*	-2.2	2.6
MRPL33	mitochondrial ribosomal protein L33	*ns*	-2.6	2.3
CDKN2C	cyclin-dependent kinase inhibitor 2C (p18, inhibits CDK4)	*ns*	-2.1	2.6
KANK2	KN motif and ankyrin repeat domains 2	*ns*	-2.6	2.5
GLB1L	galactosidase beta 1 like	*ns*	-2.6	2.0
CH25H	cholesterol 25-hydroxylase	*ns*	-2.6	2.5
ALDH3A2	aldehyde dehydrogenase 3 family, member A2	*ns*	-2.3	2.6
PLXNC1	plexin C1	*ns*	-2.3	2.5
SELPLG	selectin P ligand	*ns*	-2.1	2.5
STAB1	stabilin 1	*ns*	-2.0	2.5
HLA-DPB2	major histocompatibility complex, class II, DP beta 2 (pseudogene)	*ns*	-2.5	2.1
CACNB2	calcium channel, voltage-dependent, beta 2 subunit	*ns*	-2.5	2.2
ALKBH3	alkB homolog 3, alpha-ketoglutarate-dependent dioxygenase	*ns*	-2.1	2.5
LY75-CD302	LY75-CD302 readthrough	*ns*	-2.5	2.3
TGFBR2	transforming growth factor beta receptor II	*ns*	-2.0	2.5
XG	Xg blood group	*ns*	-2.5	2.1
ZNF271P	zinc finger protein 271, pseudogene	*ns*	-2.5	2.3
GLIPR2	GLI pathogenesis-related 2	*ns*	-2.4	2.5
SCIMP	SLP adaptor and CSK interacting membrane protein	*ns*	-2.3	2.5
EFEMP2	EGF containing fibulin-like extracellular matrix protein 2	*ns*	-2.0	2.5
ST6GALNAC6	ST6 (alpha-N-acetyl-neuraminyl-2,3-beta-galactosyl-1,3)-N-acetylgalactosaminide alpha-2,6-sialyltransferase 6	*ns*	-2.4	2.4
LARGE1	LARGE xylosyl- and glucuronyltransferase 1	*ns*	-2.2	2.4
ADCY1	adenylate cyclase 1 (brain)	*ns*	-2.1	2.4
LOC105372674	uncharacterized LOC105372674	*ns*	-2.4	2.4
SSC5D	scavenger receptor cysteine rich family, 5 domains	*ns*	-2.3	2.4
TMX4	thioredoxin-related transmembrane protein 4	*ns*	-2.1	2.4
PBX3	pre-B-cell leukemia homeobox 3	*ns*	-2.1	2.4

Development • Supplementary information

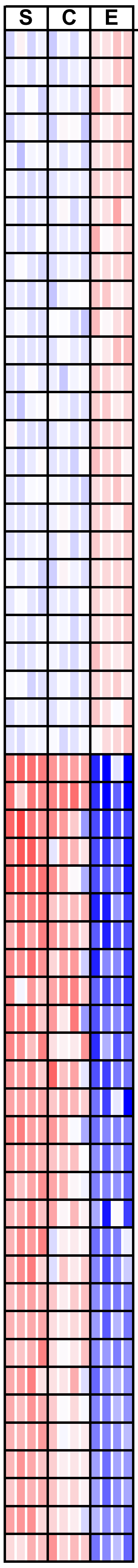

Symbol	Name	Linear Fold Change (*ns*=no significant difference) S vs C	S vs E	E vs C
RIN2	Ras and Rab interactor 2	*ns*	-2.3	2.4
TCN2	transcobalamin II	*ns*	-2.2	2.4
TRGV8	T cell receptor gamma variable 8	*ns*	-2.4	2.2
IMPA1	inositol(myo)-1(or 4)-monophosphatase 1	*ns*	-2.3	2.4
KRT37	keratin 37, type I	*ns*	-2.4	2.3
SIGLEC14	sialic acid binding Ig-like lectin 14	*ns*	-2.3	2.1
BMERB1	bMERB domain containing 1	*ns*	-2.3	2.2
SNAP91	synaptosome associated protein 91kDa	*ns*	-2.2	2.3
SGPL1	sphingosine-1-phosphate lyase 1	*ns*	-2.3	2.3
RNASET2	ribonuclease T2	*ns*	-2.3	2.1
FCGR2A	Fc fragment of IgG, low affinity IIa, receptor (CD32)	*ns*	-2.2	2.3
DNAJC12	DnaJ (Hsp40) homolog, subfamily C, member 12	*ns*	-2.3	2.2
ICAM1	intercellular adhesion molecule 1	*ns*	-2.3	2.3
NECTIN2	nectin cell adhesion molecule 2	*ns*	-2.3	2.1
TARS3	threonyl-tRNA synthetase 3	*ns*	-2.3	2.2
CTSD	cathepsin D	*ns*	-2.2	2.3
CD83	CD83 molecule	*ns*	-2.1	2.3
ATL1	atlastin GTPase 1	*ns*	-2.3	2.1
AKAP6	A kinase (PRKA) anchor protein 6	*ns*	-2.2	2.2
PLLP	plasmolipin	*ns*	-2.2	2.2
GANC	glucosidase, alpha; neutral C	*ns*	-2.2	2.0
PRNP	prion protein	*ns*	-2.1	2.1
PLXND1	plexin D1	*ns*	-2.1	2.1
HCK	HCK proto-oncogene, Src family tyrosine kinase	*ns*	-2.1	2.1
PLXNA4	plexin A4	*ns*	-2.1	2.1
ASAP2	ArfGAP with SH3 domain, ankyrin repeat and PH domain 2	*ns*	-2.1	2.0
PIRC66	piwi-interacting RNA cluster 66	*ns*	151.3	-86.8
CGB8	chorionic gonadotropin, beta polypeptide 8	*ns*	89.1	-106.9
BMP5	bone morphogenetic protein 5	*ns*	86.0	-21.1
F5	coagulation factor V (proaccelerin, labile factor)	*ns*	74.3	-20.0
EGFL6	EGF-like-domain, multiple 6	*ns*	70.0	-15.5
CYP19A1	cytochrome P450, family 19, subfamily A, polypeptide 1	*ns*	61.7	-29.4
MIR1323	microRNA 1323	*ns*	47.1	-30.7
LGALS13	lectin, galactoside-binding, soluble, 13	*ns*	43.7	-16.5
CGB3	chorionic gonadotropin subunit beta 3	*ns*	23.5	-33.7
LGALS16	lectin, galactoside-binding, soluble, 16	*ns*	29.7	-12.0
HSD3B1	hydroxy-delta-5-steroid dehydrogenase, 3 beta- and steroid delta-isomerase 1	*ns*	29.7	-12.6
DSC3	desmocollin 3	*ns*	27.2	-21.8
TFPI2	tissue factor pathway inhibitor 2	*ns*	26.0	-18.3
HGF	hepatocyte growth factor (hepapoietin A; scatter factor)	*ns*	24.1	-7.8
PAGE4	P antigen family, member 4 (prostate associated)	*ns*	19.2	-10.6
DLK1	delta-like 1 homolog (Drosophila)	*ns*	17.7	-9.6
MUC15	mucin 15, cell surface associated	*ns*	16.9	-10.2
C21orf91-OT1	C21orf91 overlapping transcript 1	*ns*	16.5	-4.3
ANGPT2	angiopoietin 2	*ns*	15.9	-6.4
CDO1	cysteine dioxygenase type 1	*ns*	15.4	-9.9
SPTLC3	serine palmitoyltransferase, long chain base subunit 3	*ns*	14.1	-7.7
ERVW-1	endogenous retrovirus group W, member 1	*ns*	13.6	-6.9
OLAH	oleoyl-ACP hydrolase	*ns*	13.3	-6.1
STS	steroid sulfatase (microsomal), isozyme S	*ns*	12.9	-5.5
RHOBTB1	Rho-related BTB domain containing 1	*ns*	12.7	-5.6
RRS1-AS1	RRS1 antisense RNA 1 (head to head)	*ns*	12.5	-6.0
SLC19A3	solute carrier family 19 (thiamine transporter), member 3	*ns*	12.3	-7.1
KATNBL1	katanin p80 subunit B-like 1	*ns*	12.1	-4.5
SIGLEC6	sialic acid binding Ig-like lectin 6	*ns*	9.8	-12.1

Development • Supplementary information

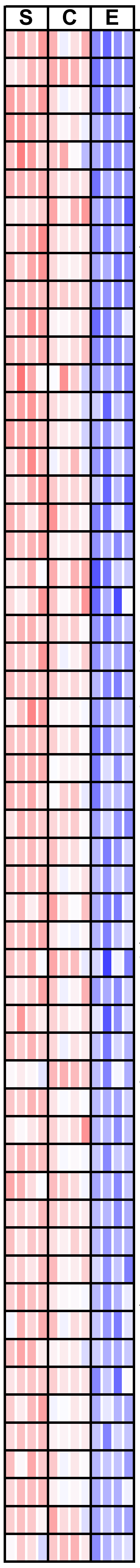

Symbol	Name	Linear Fold Change (*ns*=no significant difference) S vs C	S vs E	E vs C
LOC729739	peptidylprolyl isomerase A (cyclophilin A) pseudogene	*ns*	11.9	-8.3
PRSS12	protease, serine, 12 (neurotrypsin, motopsin)	*ns*	9.0	-11.7
ADAMTS6	ADAM metallopeptidase with thrombospondin type 1 motif 6	*ns*	11.7	-4.7
RADX	RPA1 related single stranded DNA binding protein, X-linked	*ns*	11.6	-5.0
PCDH10	protocadherin 10	*ns*	11.5	-5.0
LOC55338	uncharacterized LOC55338	*ns*	11.1	-6.4
CYP2J2	cytochrome P450, family 2, subfamily J, polypeptide 2	*ns*	7.0	-11.0
ATP6V1C2	ATPase, H+ transporting, lysosomal 42kDa, V1 subunit C2	*ns*	10.8	-6.0
CSF3R	colony stimulating factor 3 receptor	*ns*	10.8	-5.6
PEG3-AS1	PEG3 antisense RNA 1	*ns*	10.4	-7.8
GLDN	gliomedin	*ns*	10.4	-4.8
PEG3	paternally expressed 3	*ns*	10.0	-6.5
C7	complement component 7	*ns*	9.9	-5.5
KYNU	kynureninase	*ns*	9.9	-3.8
LOC101927355	uncharacterized LOC101927355	*ns*	9.5	-3.6
LOC284561	uncharacterized LOC284561	*ns*	9.5	-4.3
SPINT1	serine peptidase inhibitor, Kunitz type 1	*ns*	9.4	-6.8
MIR498	microRNA 498	*ns*	9.3	-7.8
FOXO4	forkhead box O4	*ns*	9.2	-4.7
INHBA	inhibin beta A	*ns*	6.2	-9.1
ERVH48-1	endogenous retrovirus group 48, member 1	*ns*	7.9	-8.3
SLC19A2	solute carrier family 19 (thiamine transporter), member 2	*ns*	7.9	-5.6
TP63	tumor protein p63	*ns*	7.9	-4.8
FHDC1	FH2 domain containing 1	*ns*	7.9	-5.7
TREML2	triggering receptor expressed on myeloid cells-like 2	*ns*	7.8	-3.2
KRT23	keratin 23, type I	*ns*	7.5	-4.8
PSG5	pregnancy specific beta-1-glycoprotein 5	*ns*	7.5	-3.2
PSG1	pregnancy specific beta-1-glycoprotein 1	*ns*	6.9	-4.0
LOC100505909	uncharacterized LOC100505909	*ns*	6.8	-5.1
LINC00470	long intergenic non-protein coding RNA 470	*ns*	6.7	-4.9
CMYA5	cardiomyopathy associated 5	*ns*	6.5	-3.5
LOC101927880	uncharacterized LOC101927880	*ns*	6.3	-6.5
ARHGAP32	Rho GTPase activating protein 32	*ns*	6.5	-2.9
TLR3	toll-like receptor 3	*ns*	6.1	-6.4
ERVK3-2	endogenous retrovirus group K3, member 2	*ns*	6.4	-5.0
SPDYA	speedy	*ns*	6.3	-4.7
RPS6KA5	ribosomal protein S6 kinase, 90kDa, polypeptide 5	*ns*	6.1	-3.5
ZNF732	zinc finger protein 732	*ns*	2.5	-6.0
OPHN1	oligophrenin 1	*ns*	5.9	-3.0
PLEKHA8P1	pleckstrin homology domain containing, family A member 8 pseudogene 1	*ns*	4.2	-5.9
LOC105375403	uncharacterized LOC105375403	*ns*	5.9	-2.9
LOC105377871	uncharacterized LOC105377871	*ns*	5.9	-5.0
GLDC	glycine dehydrogenase (decarboxylating)	*ns*	5.6	-4.8
GDF15	growth differentiation factor 15	*ns*	5.5	-4.3
DTL	denticleless E3 ubiquitin protein ligase homolog (Drosophila)	*ns*	5.5	-4.9
ATP10D	ATPase, class V, type 10D	*ns*	5.5	-2.7
DOCK9-AS1	DOCK9 antisense RNA 1	*ns*	5.4	-4.2
BET1	Bet1 golgi vesicular membrane trafficking protein	*ns*	5.4	-2.5
CAPN6	calpain 6	*ns*	5.4	-4.5
SDC1	syndecan 1	*ns*	5.2	-2.6
FAM13A	family with sequence similarity 13, member A	*ns*	5.1	-3.1
LARGE2	LARGE xylosyl- and glucuronyltransferase 2	*ns*	5.1	-3.3
CEMIP2	cell migration inducing hyaluronidase 2	*ns*	5.1	-2.3
RNF128	ring finger protein 128, E3 ubiquitin protein ligase	*ns*	5.1	-4.5
GPR87	G protein-coupled receptor 87	*ns*	2.9	-5.0

Development • Supplementary information

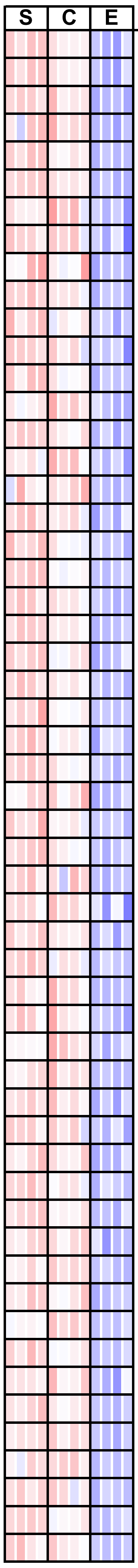

Symbol	Name	Linear Fold Change (*ns*=no significant difference) S vs C	S vs E	E vs C
SPDYE11	speedy	*ns*	4.9	-3.2
SPDYE8	speedy/RINGO cell cycle regulator family member E8	*ns*	4.9	-3.2
NEBL	nebulette	*ns*	4.9	-4.4
SLC7A5	solute carrier family 7 (amino acid transporter light chain, L system), member 5	*ns*	3.8	-4.8
SLC22A11	solute carrier family 22 (organic anion	*ns*	4.8	-3.0
MTARC1	mitochondrial amidoxime reducing component 1	*ns*	4.7	-3.5
CCNB1	cyclin B1	*ns*	3.4	-4.7
DEPDC1	DEP domain containing 1	*ns*	4.6	-4.2
TPRXL	tetra-peptide repeat homeobox-like	*ns*	4.6	-3.6
FDX1	ferredoxin 1	*ns*	4.5	-3.0
LOC101928408	uncharacterized LOC101928408	*ns*	4.5	-2.4
TOP2A	topoisomerase (DNA) II alpha	*ns*	4.4	-3.1
ATP6V1B1	ATPase, H+ transporting, lysosomal 56	*ns*	4.4	-2.6
DIAPH3	diaphanous-related formin 3	*ns*	2.8	-4.4
GAB1	GRB2-associated binding protein 1	*ns*	4.4	-3.2
ANLN	anillin actin binding protein	*ns*	2.9	-4.4
LOC105377872	uncharacterized LOC105377872	*ns*	3.0	-4.3
ST8SIA6-AS1	ST8SIA6 antisense RNA 1	*ns*	4.3	-2.9
TYMS	thymidylate synthetase	*ns*	4.3	-2.2
CLIP1	CAP-GLY domain containing linker protein 1	*ns*	4.3	-2.1
MBNL3	muscleblind-like splicing regulator 3	*ns*	4.2	-3.0
TMEM40	transmembrane protein 40	*ns*	4.2	-2.8
LOC105378019	uncharacterized LOC105378019	*ns*	4.2	-2.6
ZNF292	zinc finger protein 292	*ns*	4.2	-2.9
ZFAT-AS1	ZFAT antisense RNA 1	*ns*	4.2	-2.3
HS3ST3B1	heparan sulfate (glucosamine) 3-O-sulfotransferase 3B1	*ns*	4.1	-2.3
NEDD4L	neural precursor cell expressed, developmentally down-regulated 4-like	*ns*	4.1	-2.5
TNS4	tensin 4	*ns*	3.4	-4.1
FXYD3	FXYD domain containing ion transport regulator 3	*ns*	4.0	-2.3
MEST	mesoderm specific transcript	*ns*	4.0	-2.4
LOC105376910	uncharacterized LOC105376910	*ns*	4.0	-3.2
MET	MET proto-oncogene, receptor tyrosine kinase	*ns*	3.6	-4.0
GRAMD2A	GRAM domain containing 2A	*ns*	4.0	-3.0
MB21D2	Mab-21 domain containing 2	*ns*	4.0	-2.2
SLC38A9	solute carrier family 38, member 9	*ns*	3.4	-4.0
LOC105379521	uncharacterized LOC105379521	*ns*	4.0	-3.8
LINC01194	long intergenic non-protein coding RNA 1194	*ns*	2.2	-3.9
ITGB4	integrin beta 4	*ns*	3.2	-3.9
TENM3	teneurin transmembrane protein 3	*ns*	3.9	-3.7
CCNA2	cyclin A2	*ns*	3.9	-3.0
CDK2	cyclin-dependent kinase 2	*ns*	3.8	-3.6
STK26	serine	*ns*	3.8	-2.3
SLC38A1	solute carrier family 38, member 1	*ns*	3.8	-2.6
SLC2A1	solute carrier family 2 (facilitated glucose transporter), member 1	*ns*	3.7	-3.8
CCSAP	centriole, cilia and spindle-associated protein	*ns*	2.8	-3.8
CYP11A1	cytochrome P450, family 11, subfamily A, polypeptide 1	*ns*	3.7	-3.1
WEE1	WEE1 G2 checkpoint kinase	*ns*	2.1	-3.7
ZNF750	zinc finger protein 750	*ns*	3.7	-2.3
DPP4	dipeptidyl-peptidase 4	*ns*	3.1	-3.7
SLC5A6	solute carrier family 5 (sodium	*ns*	3.7	-2.8
FERMT1	fermitin family member 1	*ns*	3.4	-3.7
CLN3	ceroid-lipofuscinosis, neuronal 3	*ns*	2.8	-3.7
PCOLCE2	procollagen C-endopeptidase enhancer 2	*ns*	3.6	-2.9
NCOA3	nuclear receptor coactivator 3	*ns*	3.6	-2.2
LOC105377943	uncharacterized LOC105377943	*ns*	3.6	-3.0

Development • Supplementary information

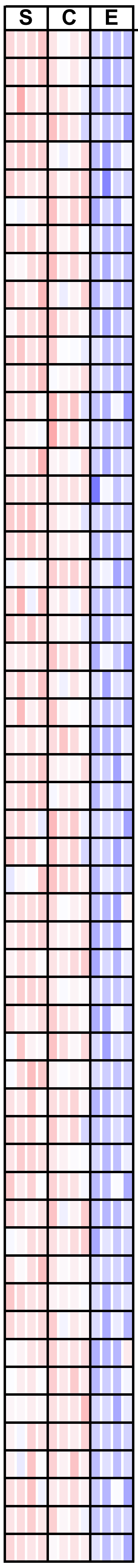

Symbol	Name	Linear Fold Change (*ns*=no significant difference) S vs C	S vs E	E vs C
FIGN	fidgetin	*ns*	3.6	-2.7
EBLN2	endogenous Bornavirus-like nucleoprotein 2	*ns*	3.6	-2.5
TBX20	T-box 20	*ns*	3.6	-2.5
PGAP1	post-GPI attachment to proteins 1	*ns*	3.6	-2.8
PHLPP1	PH domain and leucine rich repeat protein phosphatase 1	*ns*	3.6	-2.3
GDA	guanine deaminase	*ns*	3.6	-3.6
SLC29A1	solute carrier family 29 (equilibrative nucleoside transporter), member 1	*ns*	2.3	-3.6
PARD6B	par-6 family cell polarity regulator beta	*ns*	3.5	-3.0
RFC3	replication factor C subunit 3	*ns*	3.0	-3.5
FAM156B	family with sequence similarity 156, member B	*ns*	3.4	-2.6
TFAP2A-AS1	TFAP2A antisense RNA 1	*ns*	3.4	-3.2
SMAD4	SMAD family member 4	*ns*	3.4	-2.1
ADAM12	ADAM metallopeptidase domain 12	*ns*	3.4	-2.4
KNL1	kinetochore scaffold 1	*ns*	3.2	-3.4
EXO1	exonuclease 1	*ns*	2.4	-3.4
IRF6	interferon regulatory factor 6	*ns*	3.4	-2.5
CGA	glycoprotein hormones, alpha polypeptide	*ns*	3.4	-3.0
TEX10	testis expressed 10	*ns*	3.4	-2.0
RASA1	RAS p21 protein activator (GTPase activating protein) 1	*ns*	3.4	-2.5
SNORD16	small nucleolar RNA, C	*ns*	2.2	-3.3
CCDC162P	coiled-coil domain containing 162, pseudogene	*ns*	3.3	-2.5
PIP5K1B	phosphatidylinositol-4-phosphate 5-kinase, type I, beta	*ns*	3.3	-2.0
TICRR	TOPBP1-interacting checkpoint and replication regulator	*ns*	2.9	-3.3
ST7-OT4	ST7 overlapping transcript 4	*ns*	3.3	-2.1
LOC105376501	uncharacterized LOC105376501	*ns*	3.2	-2.6
SLC52A1	solute carrier family 52 (riboflavin transporter), member 1	*ns*	2.9	-3.2
IGF2BP3	insulin-like growth factor 2 mRNA binding protein 3	*ns*	3.2	-2.5
MAN1A2	mannosidase, alpha, class 1A, member 2	*ns*	3.2	-2.2
ATAD5	ATPase family, AAA domain containing 5	*ns*	2.6	-3.2
SGO1	shugoshin 1	*ns*	3.2	-2.8
LOC105374160	uncharacterized LOC105374160	*ns*	2.2	-3.2
MYH10	myosin, heavy chain 10, non-muscle	*ns*	3.2	-2.5
COBLL1	cordon-bleu WH2 repeat protein like 1	*ns*	3.1	-2.7
LRRC1	leucine rich repeat containing 1	*ns*	2.6	-3.1
RGPD2	RANBP2-like and GRIP domain containing 2	*ns*	3.1	-2.1
CEP295NL	CEP295 N-terminal like	*ns*	3.1	-2.9
STC1	stanniocalcin 1	*ns*	2.4	-3.1
ATP7B	ATPase, Cu++ transporting, beta polypeptide	*ns*	3.1	-2.0
TDRP	testis development related protein	*ns*	3.0	-3.0
UBE2T	ubiquitin conjugating enzyme E2T	*ns*	3.0	-2.5
EML4	echinoderm microtubule associated protein like 4	*ns*	3.0	-2.1
CDC6	cell division cycle 6	*ns*	3.0	-2.8
RDH13	retinol dehydrogenase 13 (all-trans	*ns*	3.0	-3.0
NET1	neuroepithelial cell transforming 1	*ns*	2.4	-3.0
P2RY6	pyrimidinergic receptor P2Y, G-protein coupled, 6	*ns*	3.0	-2.3
LINC00540	long intergenic non-protein coding RNA 540	*ns*	3.0	-2.0
TPX2	TPX2, microtubule-associated	*ns*	3.0	-2.5
SKP2	S-phase kinase-associated protein 2, E3 ubiquitin protein ligase	*ns*	2.4	-3.0
CHML	choroideremia-like (Rab escort protein 2)	*ns*	2.1	-3.0
GRHL1	grainyhead-like transcription factor 1	*ns*	2.1	-3.0
S1PR2	sphingosine-1-phosphate receptor 2	*ns*	2.5	-2.9
CCDC169	coiled-coil domain containing 169	*ns*	2.3	-2.9
STEAP4	STEAP family member 4	*ns*	2.9	-2.7
WDR76	WD repeat domain 76	*ns*	2.9	-2.4
RFESD	Rieske (Fe-S) domain containing	*ns*	2.9	-2.4

Development • Supplementary information

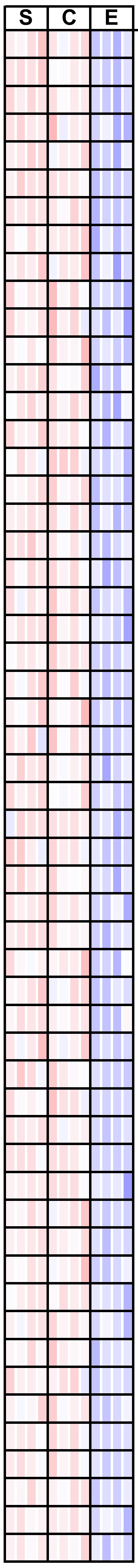

Symbol	Name	S vs C	S vs E	E vs C
		Linear Fold Change (*ns*=no significant difference)		
VGLL1	vestigial-like family member 1	*ns*	2.9	-2.6
PROSER1	proline and serine rich 1	*ns*	2.9	-2.1
ITIH5	inter-alpha-trypsin inhibitor heavy chain family, member 5	*ns*	2.9	-2.5
LOC101927853	uncharacterized LOC101927853	*ns*	2.9	-2.8
TENT5A	terminal nucleotidyltransferase 5A	*ns*	2.9	-2.4
MFSD4B	major facilitator superfamily domain containing 4B	*ns*	2.9	-2.5
LOC105377770	uncharacterized LOC105377770	*ns*	2.9	-2.8
LOC101926943	uncharacterized LOC101926943	*ns*	2.4	-2.9
GRHL2	grainyhead-like transcription factor 2	*ns*	2.8	-2.7
NUSAP1	nucleolar and spindle associated protein 1	*ns*	2.7	-2.8
CENPF	centromere protein F	*ns*	2.8	-2.8
NECTIN4	nectin cell adhesion molecule 4	*ns*	2.0	-2.8
DOCK9	dedicator of cytokinesis 9	*ns*	2.8	-2.6
TMC1	transmembrane channel like 1	*ns*	2.5	-2.8
PRC1	protein regulator of cytokinesis 1	*ns*	2.7	-2.4
SKA3	spindle and kinetochore associated complex subunit 3	*ns*	2.0	-2.7
TFAP2A	transcription factor AP-2 alpha (activating enhancer binding protein 2 alpha)	*ns*	2.4	-2.7
LOC105379715	uncharacterized LOC105379715	*ns*	2.6	-2.7
KIF2A	kinesin heavy chain member 2A	*ns*	2.7	-2.0
GTF2I	general transcription factor IIi	*ns*	2.7	-2.6
MCM5	minichromosome maintenance complex component 5	*ns*	2.3	-2.6
CENPI	centromere protein I	*ns*	2.6	-2.6
FMR1	fragile X mental retardation 1	*ns*	2.1	-2.6
EZH2	enhancer of zeste 2 polycomb repressive complex 2 subunit	*ns*	2.6	-2.6
EZR	ezrin	*ns*	2.5	-2.6
GPSM2	G-protein signaling modulator 2	*ns*	2.3	-2.6
AHSA2P	activator of HSP90 ATPase homolog 2, pseudogene	*ns*	2.6	-2.1
LIMK2	LIM domain kinase 2	*ns*	2.6	-2.1
LOC105370921	uncharacterized LOC105370921	*ns*	2.5	-2.6
IMMP2L	inner mitochondrial membrane peptidase subunit 2	*ns*	2.6	-2.4
GLCCI1	glucocorticoid induced 1	*ns*	2.6	-2.1
UBE2C	ubiquitin-conjugating enzyme E2C	*ns*	2.5	-2.5
RTTN	rotatin	*ns*	2.5	-2.1
MCTS2P	malignant T-cell amplified sequence 2, pseudogene	*ns*	2.1	-2.5
ATF3	activating transcription factor 3	*ns*	2.5	-2.2
MAST4	microtubule associated serine	*ns*	2.5	-2.2
DNMT1	DNA (cytosine-5-)-methyltransferase 1	*ns*	2.5	-2.2
MBTD1	mbt domain containing 1	*ns*	2.5	-2.3
SGO1-AS1	SGO1 antisense RNA 1	*ns*	2.0	-2.4
POLA1	polymerase (DNA directed), alpha 1, catalytic subunit	*ns*	2.4	-2.1
DCP2	decapping mRNA 2	*ns*	2.4	-2.4
DKFZP586I1420	uncharacterized protein DKFZp586I1420	*ns*	2.2	-2.4
ANGPTL4	angiopoietin like 4	*ns*	2.2	-2.3
LGALS8	lectin, galactoside-binding, soluble, 8	*ns*	2.3	-2.2
PPP1R15B	protein phosphatase 1, regulatory subunit 15B	*ns*	2.1	-2.3
SCARNA9	small Cajal body-specific RNA 9	*ns*	2.2	-2.2
ERVMER34-1	endogenous retrovirus group MER34, member 1	*ns*	2.0	-2.2
DACT2	dishevelled-binding antagonist of beta-catenin 2	*ns*	2.0	-2.2
CDCA2	cell division cycle associated 2	*ns*	2.2	-2.0
SLC29A2	solute carrier family 29 (equilibrative nucleoside transporter), member 2	*ns*	2.0	-2.2
ADK	adenosine kinase	*ns*	2.0	-2.2
TBC1D3E	TBC1 domain family, member 3E	*ns*	2.0	-2.2
CYRIA	CYFIP related Rac1 interactor A	*ns*	2.1	-2.1
TMEM30B	transmembrane protein 30B	*ns*	2.0	-2.1
FAM117B	family with sequence similarity 117, member B	*ns*	2.0	-2.1

Development • Supplementary information

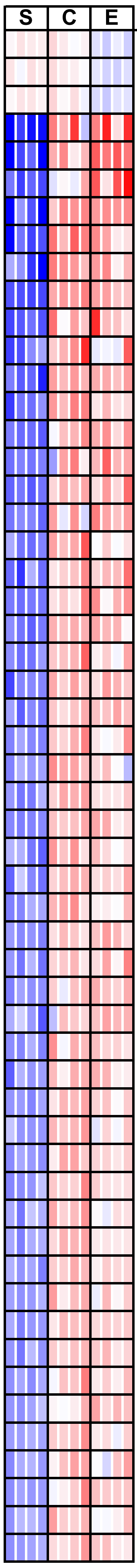

Symbol	Name	Linear Fold Change (*ns*=no significant difference) S vs C	S vs E	E vs C
ZNF808	zinc finger protein 808	*ns*	*2.1*	*-2.1*
SPC25	SPC25, NDC80 kinetochore complex component	*ns*	*2.0*	*-2.1*
KIF23	kinesin family member 23	*ns*	*2.0*	*-2.0*
MMP12	matrix metallopeptidase 12	-75.0	*-173.6*	*ns*
IGFBP1	insulin like growth factor binding protein 1	-53.3	*-151.0*	*ns*
EPYC	epiphycan	-11.1	*-74.0*	*ns*
PRG2	proteoglycan 2, bone marrow (natural killer cell activator, eosinophil granule major basic protein)	-66.5	*-73.7*	*ns*
MT1H	metallothionein 1H	-67.7	*-30.5*	*ns*
LAIR2	leukocyte-associated immunoglobulin-like receptor 2	-45.2	*-31.5*	*ns*
NOTUM	notum pectinacetylesterase homolog (Drosophila)	-44.8	*-31.3*	*ns*
MT1G	metallothionein 1G	-26.2	*-32.8*	*ns*
HNRNPA1P33	heterogeneous nuclear ribonucleoprotein A1 pseudogene 33	-32.1	*-10.2*	*ns*
HTRA4	HtrA serine peptidase 4	-30.0	*-28.7*	*ns*
HCAR2	hydroxycarboxylic acid receptor 2	-29.7	*-12.5*	*ns*
AOC1	amine oxidase, copper containing 1	-16.6	*-22.5*	*ns*
LINC01602	long intergenic non-protein coding RNA 1602	-9.7	*-22.4*	*ns*
MT1X	metallothionein 1X	-18.9	*-21.9*	*ns*
MAMDC2	MAM domain containing 2	-9.4	*-21.0*	*ns*
DIO2	deiodinase, iodothyronine, type II	-20.9	*-6.7*	*ns*
PDCD1LG2	programmed cell death 1 ligand 2	-16.0	*-20.7*	*ns*
IL1R2	interleukin 1 receptor, type II	-14.1	*-17.6*	*ns*
MT1F	metallothionein 1F	-17.2	*-14.0*	*ns*
RASGRF2	Ras protein-specific guanine nucleotide-releasing factor 2	-17.1	*-8.7*	*ns*
PLA2G10	phospholipase A2, group X	-16.4	*-15.2*	*ns*
ISG15	ISG15 ubiquitin-like modifier	-11.9	*-14.0*	*ns*
TNFSF10	tumor necrosis factor (ligand) superfamily, member 10	-12.8	*-7.7*	*ns*
HAPLN3	hyaluronan and proteoglycan link protein 3	-12.6	*-4.3*	*ns*
PLAC8	placenta specific 8	-12.5	*-7.1*	*ns*
HSPG2	heparan sulfate proteoglycan 2	-12.3	*-11.0*	*ns*
DEPDC7	DEP domain containing 7	-12.3	*-7.1*	*ns*
GLIPR1	GLI pathogenesis-related 1	-11.7	*-9.4*	*ns*
SLC16A6	solute carrier family 16, member 6	-11.4	*-5.7*	*ns*
RFPL4B	ret finger protein-like 4B	-8.5	*-11.2*	*ns*
DAPP1	dual adaptor of phosphotyrosine and 3-phosphoinositides	-8.7	*-11.0*	*ns*
MMP2	matrix metallopeptidase 2	-7.7	*-10.8*	*ns*
PLA2G7	phospholipase A2, group VII (platelet-activating factor acetylhydrolase, plasma)	-5.2	*-10.4*	*ns*
GPR32	G protein-coupled receptor 32	-10.1	*-7.8*	*ns*
FAT2	FAT atypical cadherin 2	-9.8	*-8.1*	*ns*
NOG	noggin	-9.7	*-6.9*	*ns*
CST6	cystatin E	-9.6	*-4.4*	*ns*
SLC4A7	solute carrier family 4, sodium bicarbonate cotransporter, member 7	-9.4	*-6.9*	*ns*
TCF7L2	transcription factor 7-like 2 (T-cell specific, HMG-box)	-9.3	*-6.0*	*ns*
SDC4	syndecan 4	-9.2	*-3.9*	*ns*
PTGES	prostaglandin E synthase	-9.1	*-4.8*	*ns*
CCR1	chemokine (C-C motif) receptor 1	-8.8	*-6.2*	*ns*
MYCNUT	MYCN upstream transcript (non-protein coding)	-8.7	*-5.2*	*ns*
JPT1	Jupiter microtubule associated homolog 1	-8.6	*-7.4*	*ns*
QSOX1	quiescin Q6 sulfhydryl oxidase 1	-8.5	*-7.6*	*ns*
REPS2	RALBP1 associated Eps domain containing 2	-8.5	*-6.3*	*ns*
SERPING1	serpin peptidase inhibitor, clade G (C1 inhibitor), member 1	-4.9	*-8.3*	*ns*
FLNB	filamin B, beta	-8.3	*-4.5*	*ns*
UPK1B	uroplakin 1B	-8.3	*-5.2*	*ns*
IL2RB	interleukin 2 receptor, beta	-7.3	*-8.2*	*ns*
GREB1L	growth regulation by estrogen in breast cancer-like	-7.7	*-4.0*	*ns*
ADAM19	ADAM metallopeptidase domain 19	-7.6	*-6.2*	*ns*

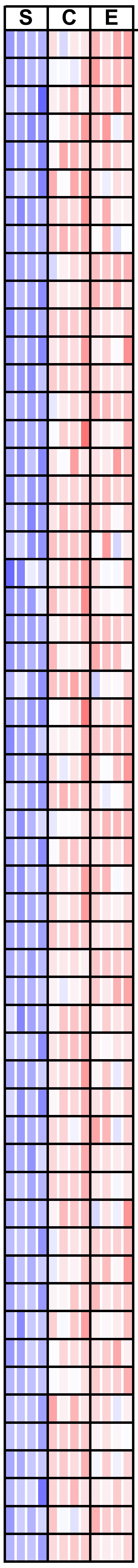

Symbol	Name	Linear Fold Change (*ns*=no significant difference) S vs C	S vs E	E vs C
PLAUR	plasminogen activator, urokinase receptor	-3.4	-7.5	ns
CRISPLD2	cysteine-rich secretory protein LCCL domain containing 2	-3.3	-7.5	ns
SAMD9	sterile alpha motif domain containing 9	-5.2	-7.4	ns
SPRR2G	small proline-rich protein 2G	-6.6	-7.3	ns
GALNT3	polypeptide N-acetylgalactosaminyltransferase 3	-7.3	-6.1	ns
IL10RA	interleukin 10 receptor, alpha	-7.2	-3.6	ns
SNAI1	snail family zinc finger 1	-7.1	-4.9	ns
CCNE1	cyclin E1	-7.0	-3.4	ns
IFI27	interferon, alpha-inducible protein 27	-3.5	-7.0	ns
CITED2	Cbp	-5.3	-7.0	ns
FN1	fibronectin 1	-6.9	-5.5	ns
TGFB2	transforming growth factor beta 2	-6.8	-6.3	ns
C12orf75	chromosome 12 open reading frame 75	-6.8	-5.4	ns
HUNK	hormonally up-regulated Neu-associated kinase	-4.0	-6.8	ns
CLNK	cytokine-dependent hematopoietic cell linker	-6.7	-4.7	ns
IFIT1	interferon-induced protein with tetratricopeptide repeats 1	-5.5	-6.7	ns
TAP1	transporter 1, ATP-binding cassette, sub-family B (MDR	-5.9	-6.6	ns
GALNT7	polypeptide N-acetylgalactosaminyltransferase 7	-6.6	-5.7	ns
RAB6C-AS1	RAB6C antisense RNA 1	-6.6	-3.7	ns
HTRA1	HtrA serine peptidase 1	-6.5	-4.4	ns
TPM1	tropomyosin 1 (alpha)	-6.5	-3.7	ns
HLA-G	major histocompatibility complex, class I, G	-5.3	-6.5	ns
MXRA8	matrix-remodelling associated 8	-4.7	-6.4	ns
MIR181A2HG	MIR181A2 host gene	-6.4	-2.7	ns
GPRC5A	G protein-coupled receptor, class C, group 5, member A	-6.4	-6.2	ns
TIMP3	TIMP metallopeptidase inhibitor 3	-4.4	-6.2	ns
MX1	MX dynamin-like GTPase 1	-5.1	-6.2	ns
CHRM5	cholinergic receptor, muscarinic 5	-6.2	-3.6	ns
UBE2L6	ubiquitin-conjugating enzyme E2L 6	-2.8	-5.9	ns
KDELR3	KDEL (Lys-Asp-Glu-Leu) endoplasmic reticulum protein retention receptor 3	-5.4	-5.8	ns
ISM2	isthmin 2	-5.8	-4.9	ns
LGALS3BP	lectin, galactoside-binding, soluble, 3 binding protein	-5.7	-4.3	ns
EPSTI1	epithelial stromal interaction 1 (breast)	-5.7	-4.8	ns
LAMA4	laminin, alpha 4	-3.7	-5.7	ns
IFITM1	interferon induced transmembrane protein 1	-2.8	-5.6	ns
LVRN	laeverin	-5.5	-4.5	ns
UBL3	ubiquitin-like 3	-5.4	-3.3	ns
PTPRF	protein tyrosine phosphatase, receptor type, F	-5.4	-3.7	ns
ARHGDIB	Rho GDP dissociation inhibitor (GDI) beta	-5.3	-4.8	ns
HCG4	HLA complex group 4 (non-protein coding)	-4.5	-5.2	ns
SH3PXD2B	SH3 and PX domains 2B	-5.2	-4.1	ns
SOX4	SRY box 4	-5.2	-3.3	ns
ART4	ADP-ribosyltransferase 4 (Dombrock blood group)	-5.2	-3.5	ns
UGDH	UDP-glucose 6-dehydrogenase	-3.6	-5.2	ns
PIPOX	pipecolic acid oxidase	-3.3	-5.1	ns
SOAT1	sterol O-acyltransferase 1	-3.9	-5.1	ns
GALNT6	polypeptide N-acetylgalactosaminyltransferase 6	-5.0	-3.7	ns
COL12A1	collagen, type XII, alpha 1	-4.2	-5.0	ns
NPC2	Niemann-Pick disease, type C2	-2.8	-5.0	ns
SCUBE1	signal peptide, CUB domain, EGF-like 1	-5.0	-3.2	ns
NRIP1	nuclear receptor interacting protein 1	-4.9	-2.8	ns
GPR78	G protein-coupled receptor 78	-4.9	-3.1	ns
IFIH1	interferon induced, with helicase C domain 1	-4.9	-3.9	ns
TIMP1	TIMP metallopeptidase inhibitor 1	-2.9	-4.9	ns
RAB8B	RAB8B, member RAS oncogene family	-4.8	-3.7	ns

Development • Supplementary information

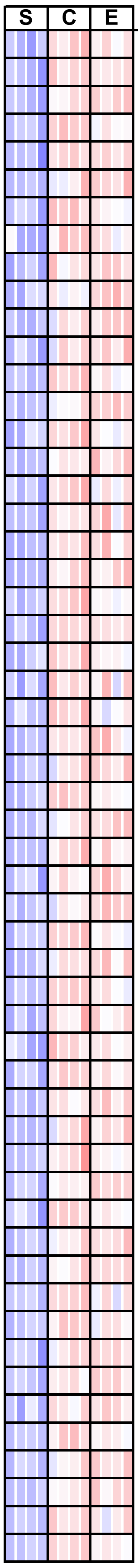

Symbol	Name	S vs C	S vs E	E vs C
		Linear Fold Change (*ns*=no significant difference)		
CD276	CD276 molecule	-4.7	-3.4	*ns*
GRK3	G protein-coupled receptor kinase 3	-4.7	-3.8	*ns*
PARP9	poly(ADP-ribose) polymerase family member 9	-3.5	-4.6	*ns*
LRRC32	leucine rich repeat containing 32	-4.6	-2.5	*ns*
MGAT4A	mannosyl (alpha-1,3-)-glycoprotein beta-1,4-N-acetylglucosaminyltransferase, isozyme A	-4.6	-4.5	*ns*
LTBP1	latent transforming growth factor beta binding protein 1	-2.9	-4.6	*ns*
P4HA1	prolyl 4-hydroxylase, alpha polypeptide I	-4.5	-2.5	*ns*
LOC105379311	uncharacterized LOC105379311	-4.5	-3.0	*ns*
GBP2	guanylate binding protein 2, interferon-inducible	-4.0	-4.5	*ns*
GAL	galanin	-2.3	-4.5	*ns*
LAYN	layilin	-3.0	-4.5	*ns*
CPED1	cadherin-like and PC-esterase domain containing 1	-3.2	-4.4	*ns*
LOC105377105	uncharacterized LOC105377105	-4.4	-2.5	*ns*
HLA-E	major histocompatibility complex, class I, E	-2.6	-4.4	*ns*
ITGA5	integrin alpha 5	-4.4	-2.4	*ns*
GCNT1	glucosaminyl (N-acetyl) transferase 1, core 2	-2.9	-4.4	*ns*
GDPD3	glycerophosphodiester phosphodiesterase domain containing 3	-4.4	-2.6	*ns*
CYP26A1	cytochrome P450, family 26, subfamily A, polypeptide 1	-3.3	-4.4	*ns*
FHOD3	formin homology 2 domain containing 3	-4.3	-4.4	*ns*
OAS1	2-5-oligoadenylate synthetase 1	-4.1	-4.4	*ns*
PXN	paxillin	-4.3	-2.2	*ns*
DNAPTP3	histone demethylase UTY-like	-4.3	-3.7	*ns*
MTSS2	MTSS I-BAR domain containing 2	-4.3	-2.4	*ns*
SLC25A15	solute carrier family 25 (mitochondrial carrier; ornithine transporter) member 15	-4.2	-3.4	*ns*
IGFBP3	insulin like growth factor binding protein 3	-4.2	-2.1	*ns*
ST6GALNAC2	ST6 (alpha-N-acetyl-neuraminyl-2,3-beta-galactosyl-1,3)-N-acetylgalactosaminide alpha-2,6-sialyltransferase 2	-3.8	-4.2	*ns*
ACSL5	acyl-CoA synthetase long-chain family member 5	-3.0	-4.2	*ns*
KRT19	keratin 19, type I	-4.2	-2.4	*ns*
HLA-C	major histocompatibility complex, class I, C	-2.6	-4.2	*ns*
ASAP3	ArfGAP with SH3 domain, ankyrin repeat and PH domain 3	-4.2	-3.2	*ns*
TSPO	translocator protein (18kDa)	-2.9	-4.2	*ns*
PEA15	phosphoprotein enriched in astrocytes 15	-2.2	-4.1	*ns*
LPCAT1	lysophosphatidylcholine acyltransferase 1	-4.1	-2.1	*ns*
FBXO32	F-box protein 32	-3.1	-4.1	*ns*
PCSK6	proprotein convertase subtilisin	-4.1	-2.4	*ns*
ADAMTS1	ADAM metallopeptidase with thrombospondin type 1 motif 1	-4.1	-3.7	*ns*
LINC01296	long intergenic non-protein coding RNA 1296	-4.1	-2.9	*ns*
HPGD	hydroxyprostaglandin dehydrogenase 15-(NAD)	-4.0	-3.0	*ns*
SEMA3C	semaphorin 3C	-3.0	-4.0	*ns*
PTGER3	prostaglandin E receptor 3 (subtype EP3)	-3.6	-4.0	*ns*
AFAP1	actin filament associated protein 1	-4.0	-2.4	*ns*
IL1R1	interleukin 1 receptor, type I	-2.5	-3.9	*ns*
PLOD2	procollagen-lysine, 2-oxoglutarate 5-dioxygenase 2	-3.9	-2.4	*ns*
STAT1	signal transducer and activator of transcription 1	-2.9	-3.8	*ns*
APOL6	apolipoprotein L, 6	-3.2	-3.8	*ns*
KAT14	lysine acetyltransferase 14	-3.8	-2.2	*ns*
LY6K	lymphocyte antigen 6 complex, locus K	-3.7	-2.5	*ns*
UGCG	UDP-glucose ceramide glucosyltransferase	-3.1	-3.7	*ns*
DHRS7	dehydrogenase	-2.8	-3.7	*ns*
CNN2	calponin 2	-3.3	-3.7	*ns*
HCAR1	hydroxycarboxylic acid receptor 1	-3.7	-2.4	*ns*
CSGALNACT2	chondroitin sulfate N-acetylgalactosaminyltransferase 2	-2.5	-3.7	*ns*
PARP4	poly(ADP-ribose) polymerase family member 4	-2.8	-3.6	*ns*
PLSCR1	phospholipid scramblase 1	-3.6	-2.7	*ns*
MYADM	myeloid-associated differentiation marker	-3.6	-2.4	*ns*

Development • Supplementary information

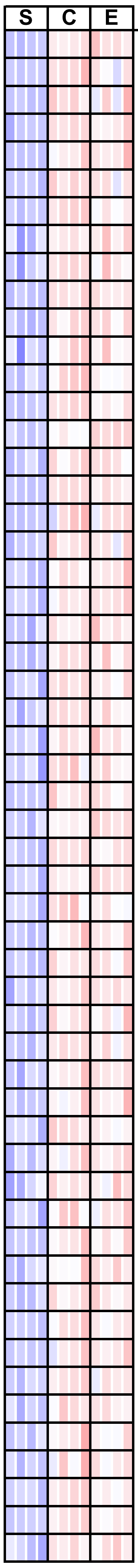

Symbol	Name	Linear Fold Change (*ns*=no significant difference) S vs C	S vs E	E vs C
PTPRS	protein tyrosine phosphatase, receptor type, S	-3.0	-3.5	*ns*
EXTL3	exostosin-like glycosyltransferase 3	-3.5	-2.2	*ns*
MACIR	macrophage immunometabolism regulator	-3.5	-2.8	*ns*
LIMA1	LIM domain and actin binding 1	-3.5	-3.1	*ns*
ETS2	v-ets avian erythroblastosis virus E26 oncogene homolog 2	-2.9	-3.5	*ns*
PDIA5	protein disulfide isomerase family A, member 5	-3.5	-2.6	*ns*
FHL2	four and a half LIM domains 2	-3.5	-2.4	*ns*
HLA-F-AS1	HLA-F antisense RNA 1	-2.8	-3.5	*ns*
MEX3D	mex-3 RNA binding family member D	-3.5	-2.9	*ns*
SLC2A10	solute carrier family 2 (facilitated glucose transporter), member 10	-3.5	-3.1	*ns*
GCH1	GTP cyclohydrolase 1	-3.4	-3.4	*ns*
COTL1	coactosin-like F-actin binding protein 1	-3.4	-2.9	*ns*
CRACDL	CRACD like	-3.4	-2.1	*ns*
SLC15A3	solute carrier family 15 (oligopeptide transporter), member 3	-3.4	-2.6	*ns*
PSMB8	proteasome subunit beta 8	-2.1	-3.4	*ns*
TNFAIP2	tumor necrosis factor, alpha-induced protein 2	-3.4	-3.1	*ns*
CMTM3	CKLF-like MARVEL transmembrane domain containing 3	-3.3	-2.8	*ns*
RRAD	Ras-related associated with diabetes	-3.3	-2.9	*ns*
PLK2	polo-like kinase 2	-3.3	-2.7	*ns*
MEGF9	multiple EGF-like-domains 9	-2.6	-3.3	*ns*
SSR3	signal sequence receptor, gamma (translocon-associated protein gamma)	-2.5	-3.2	*ns*
ECE1	endothelin converting enzyme 1	-2.9	-3.2	*ns*
TUBB2A	tubulin, beta 2A class IIa	-3.2	-3.2	*ns*
SH3D19	SH3 domain containing 19	-3.2	-2.8	*ns*
ASCL2	achaete-scute family bHLH transcription factor 2	-3.2	-2.7	*ns*
LACTB	lactamase, beta	-2.7	-3.2	*ns*
CLN5	ceroid-lipofuscinosis, neuronal 5	-3.2	-2.8	*ns*
PLOD1	procollagen-lysine, 2-oxoglutarate 5-dioxygenase 1	-3.2	-2.8	*ns*
WIPF1	WAS	-2.2	-3.2	*ns*
ERP27	endoplasmic reticulum protein 27	-3.2	-2.7	*ns*
IL13RA1	interleukin 13 receptor, alpha 1	-2.4	-3.2	*ns*
RPS27L	ribosomal protein S27-like	-3.2	-2.2	*ns*
SERPINE2	serpin peptidase inhibitor, clade E (nexin, plasminogen activator inhibitor type 1), member 2	-3.1	-3.0	*ns*
KCNK12	potassium channel, two pore domain subfamily K, member 12	-3.0	-3.1	*ns*
KLF6	Kruppel-like factor 6	-3.0	-3.1	*ns*
HEG1	heart development protein with EGF-like domains 1	-3.1	-2.8	*ns*
FAM110A	family with sequence similarity 110, member A	-3.0	-2.6	*ns*
PLOD3	procollagen-lysine, 2-oxoglutarate 5-dioxygenase 3	-3.0	-3.0	*ns*
MICAL2	microtubule associated monooxygenase, calponin and LIM domain containing 2	-2.6	-3.0	*ns*
SESTD1	SEC14 and spectrin domains 1	-3.0	-2.2	*ns*
PARP12	poly(ADP-ribose) polymerase family member 12	-2.8	-3.0	*ns*
CCN1	cellular communication network factor 1	-3.0	-3.0	*ns*
OCIAD2	OCIA domain containing 2	-3.0	-2.9	*ns*
RHOU	ras homolog family member U	-3.0	-2.8	*ns*
PIGT	phosphatidylinositol glycan anchor biosynthesis class T	-2.5	-3.0	*ns*
MMP14	matrix metallopeptidase 14 (membrane-inserted)	-3.0	-2.9	*ns*
CD9	CD9 molecule	-3.0	-2.5	*ns*
SYNE1	spectrin repeat containing, nuclear envelope 1	-2.6	-3.0	*ns*
EIF4EBP1	eukaryotic translation initiation factor 4E binding protein 1	-3.0	-2.4	*ns*
PGRMC1	progesterone receptor membrane component 1	-2.9	-2.7	*ns*
JAK1	Janus kinase 1	-2.9	-2.3	*ns*
GALM	galactose mutarotase (aldose 1-epimerase)	-2.9	-2.7	*ns*
STXBP5	syntaxin binding protein 5 (tomosyn)	-2.9	-2.1	*ns*
TRIM14	tripartite motif containing 14	-2.9	-2.4	*ns*
HACD4	3-hydroxyacyl-CoA dehydratase 4	-2.7	-2.9	*ns*

Development • Supplementary information

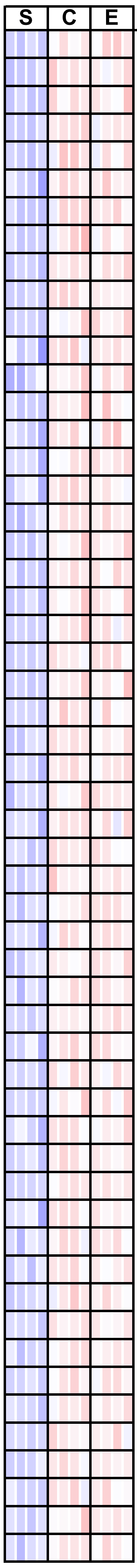

Symbol	Name	Linear Fold Change (*ns*=no significant difference) S vs C	S vs E	E vs C
LINC00324	long intergenic non-protein coding RNA 324	-2.3	-2.9	*ns*
CEACAM1	carcinoembryonic antigen-related cell adhesion molecule 1 (biliary glycoprotein)	-2.8	-2.4	*ns*
EHBP1	EH domain binding protein 1	-2.8	-2.8	*ns*
TUBB6	tubulin, beta 6 class V	-2.8	-2.2	*ns*
C5orf46	chromosome 5 open reading frame 46	-2.8	-2.3	*ns*
PRDX4	peroxiredoxin 4	-2.8	-2.8	*ns*
RABAC1	Rab acceptor 1 (prenylated)	-2.8	-2.7	*ns*
EFNB1	ephrin-B1	-2.8	-2.2	*ns*
TES	testin LIM domain protein	-2.7	-2.8	*ns*
CDC42SE2	CDC42 small effector 2	-2.8	-2.2	*ns*
ZC3HAV1	zinc finger CCCH-type, antiviral 1	-2.3	-2.8	*ns*
GPR180	G protein-coupled receptor 180	-2.7	-2.6	*ns*
AIF1L	allograft inflammatory factor 1-like	-2.7	-2.7	*ns*
VASP	vasodilator-stimulated phosphoprotein	-2.7	-2.4	*ns*
RPL39L	ribosomal protein L39-like	-2.5	-2.7	*ns*
DTX3L	deltex 3 like, E3 ubiquitin ligase	-2.3	-2.7	*ns*
DENND1B	DENN	-2.7	-2.0	*ns*
ADA	adenosine deaminase	-2.6	-2.6	*ns*
SP110	SP110 nuclear body protein	-2.6	-2.6	*ns*
PLAAT4	phospholipase A and acyltransferase 4	-2.5	-2.6	*ns*
ATP11A	ATPase, class VI, type 11A	-2.6	-2.6	*ns*
P3H1	prolyl 3-hydroxylase 1	-2.6	-2.3	*ns*
KCTD12	potassium channel tetramerization domain containing 12	-2.2	-2.6	*ns*
NCOA1	nuclear receptor coactivator 1	-2.6	-2.6	*ns*
SLC50A1	solute carrier family 50 (sugar efflux transporter), member 1	-2.6	-2.1	*ns*
LPCAT4	lysophosphatidylcholine acyltransferase 4	-2.5	-2.2	*ns*
TMEM14A	transmembrane protein 14A	-2.5	-2.5	*ns*
GTPBP1	GTP binding protein 1	-2.4	-2.5	*ns*
GPX8	glutathione peroxidase 8 (putative)	-2.5	-2.2	*ns*
FYB1	FYN binding protein 1	-2.5	-2.1	*ns*
HSPA2	heat shock 70kDa protein 2	-2.5	-2.4	*ns*
ASPSCR1	alveolar soft part sarcoma chromosome region, candidate 1	-2.1	-2.5	*ns*
RARS2	arginyl-tRNA synthetase 2, mitochondrial	-2.2	-2.5	*ns*
GYS1	glycogen synthase 1 (muscle)	-2.0	-2.4	*ns*
FADS3	fatty acid desaturase 3	-2.3	-2.4	*ns*
CAPN2	calpain 2, (m	-2.0	-2.4	*ns*
CPM	carboxypeptidase M	-2.4	-2.4	*ns*
DDX58	DEAD (Asp-Glu-Ala-Asp) box polypeptide 58	-2.4	-2.2	*ns*
IFNGR2	interferon gamma receptor 2 (interferon gamma transducer 1)	-2.3	-2.4	*ns*
PLS3	plastin 3	-2.3	-2.2	*ns*
IFNAR2	interferon (alpha, beta and omega) receptor 2	-2.3	-2.1	*ns*
FSTL1	follistatin like 1	-2.3	-2.3	*ns*
IFNAR1	interferon (alpha, beta and omega) receptor 1	-2.3	-2.0	*ns*
PSMB10	proteasome subunit beta 10	-2.2	-2.3	*ns*
APH1B	APH1B gamma secretase subunit	-2.3	-2.1	*ns*
CAPG	capping protein (actin filament), gelsolin-like	-2.3	-2.2	*ns*
ADAM9	ADAM metallopeptidase domain 9	-2.3	-2.0	*ns*
ADAM15	ADAM metallopeptidase domain 15	-2.2	-2.2	*ns*
ID1	inhibitor of DNA binding 1, dominant negative helix-loop-helix protein	-2.2	-2.2	*ns*
ABRACL	ABRA C-terminal like	-2.2	-2.2	*ns*
DPP7	dipeptidyl-peptidase 7	-2.2	-2.1	*ns*
LOC100132215	uncharacterized LOC100132215	-2.2	-2.1	*ns*
POGLUT2	protein O-glucosyltransferase 2	-2.2	-2.1	*ns*
LASP1	LIM and SH3 protein 1	-2.2	-2.2	*ns*
SIK1	salt-inducible kinase 1	-2.2	-2.0	*ns*

Development • Supplementary information

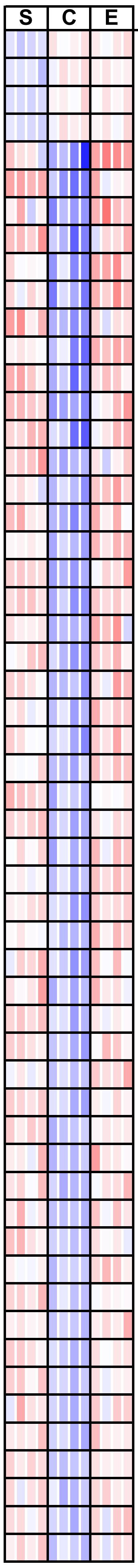

Symbol	Name	Linear Fold Change (*ns*=no significant difference) S vs C	S vs E	E vs C
SP100	SP100 nuclear antigen	-2.1	-2.2	*ns*
CHIC2	cysteine rich hydrophobic domain 2	-2.1	-2.1	*ns*
MGLL	monoglyceride lipase	-2.1	-2.1	*ns*
PRCP	prolylcarboxypeptidase	-2.0	-2.0	*ns*
ADGRL4	adhesion G protein-coupled receptor L4	6.6	*ns*	22.0
KDR	kinase insert domain receptor	13.1	*ns*	5.8
SNRPN	small nuclear ribonucleoprotein polypeptide N	4.6	*ns*	11.4
CD34	CD34 molecule	10.1	*ns*	5.9
CLEC3B	C-type lectin domain family 3, member B	4.1	*ns*	10.1
TM4SF1	transmembrane 4 L six family member 1	4.8	*ns*	10.0
PREX2	phosphatidylinositol-3,4,5-trisphosphate-dependent Rac exchange factor 2	9.6	*ns*	4.4
A2M	alpha-2-macroglobulin	4.0	*ns*	9.0
IL33	interleukin 33	8.6	*ns*	6.5
MYCT1	myc target 1	8.2	*ns*	7.0
RAMP2	receptor (G protein-coupled) activity modifying protein 2	7.8	*ns*	6.4
CACHD1	cache domain containing 1	6.3	*ns*	3.2
CALCRL	calcitonin receptor like receptor	2.8	*ns*	6.1
TRPC6	transient receptor potential cation channel, subfamily C, member 6	6.1	*ns*	5.9
IL1A	interleukin 1 alpha	3.0	*ns*	5.8
S1PR1	sphingosine-1-phosphate receptor 1	5.8	*ns*	5.7
ARHGEF6	Rac	5.8	*ns*	5.6
PPP1R14A	protein phosphatase 1, regulatory (inhibitor) subunit 14A	3.6	*ns*	5.4
ACTA2	actin, alpha 2, smooth muscle, aorta	4.4	*ns*	5.3
IDO1	indoleamine 2,3-dioxygenase 1	4.1	*ns*	5.2
DYNC2H1	dynein, cytoplasmic 2, heavy chain 1	2.7	*ns*	4.9
GMFG	glia maturation factor, gamma	3.3	*ns*	4.8
MERTK	MER proto-oncogene, tyrosine kinase	2.7	*ns*	4.8
PRKAR2B	protein kinase, cAMP-dependent, regulatory, type II, beta	4.8	*ns*	2.5
WLS	wntless Wnt ligand secretion mediator	4.7	*ns*	3.4
ZSWIM6	zinc finger, SWIM-type containing 6	3.3	*ns*	4.6
SDC2	syndecan 2	2.3	*ns*	4.3
IGF1	insulin-like growth factor 1 (somatomedin C)	3.5	*ns*	4.3
IRS1	insulin receptor substrate 1	2.4	*ns*	4.3
EBF1	early B-cell factor 1	4.2	*ns*	3.9
NID1	nidogen 1	4.2	*ns*	3.7
NPL	N-acetylneuraminate pyruvate lyase (dihydrodipicolinate synthase)	4.1	*ns*	2.4
VAMP5	vesicle associated membrane protein 5	4.1	*ns*	4.0
DOCK4	dedicator of cytokinesis 4	2.1	*ns*	4.1
SNAI2	snail family zinc finger 2	4.0	*ns*	2.7
ASS1	argininosuccinate synthase 1	3.9	*ns*	2.9
ABCC9	ATP binding cassette subfamily C member 9	3.1	*ns*	3.8
TCF4	transcription factor 4	3.8	*ns*	3.7
DEFB109A	defensin beta 109A (pseudogene)	3.5	*ns*	3.7
ICA1	islet cell autoantigen 1	3.7	*ns*	2.3
SPRY2	sprouty RTK signaling antagonist 2	2.1	*ns*	3.7
MTSS1	metastasis suppressor 1	3.7	*ns*	2.2
PTPRB	protein tyrosine phosphatase, receptor type, B	2.7	*ns*	3.6
FAT4	FAT atypical cadherin 4	3.5	*ns*	2.9
EPB41L2	erythrocyte membrane protein band 4.1-like 2	3.5	*ns*	2.3
PWAR5	Prader Willi	3.4	*ns*	3.5
MEIS1	Meis homeobox 1	3.4	*ns*	3.3
ANKRD44	ankyrin repeat domain 44	3.4	*ns*	2.7
CD93	CD93 molecule	2.9	*ns*	3.2
ETS1	v-ets avian erythroblastosis virus E26 oncogene homolog 1	2.9	*ns*	3.2
MEF2C	myocyte enhancer factor 2C	3.2	*ns*	2.5

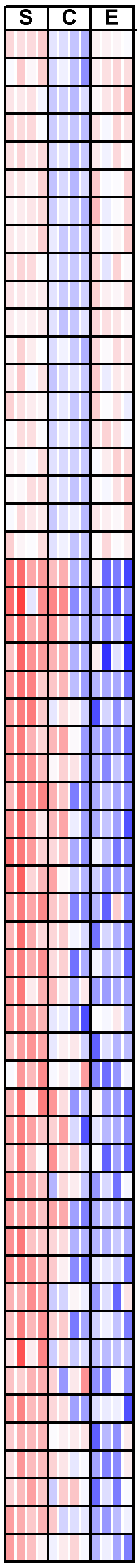

Symbol	Name	Linear Fold Change (*ns*=no significant difference) S vs C	S vs E	E vs C
EML1	echinoderm microtubule associated protein like 1	3.2	*ns*	2.1
LOC105373192	uncharacterized LOC105373192	3.0	*ns*	3.2
PIK3R3	phosphoinositide-3-kinase, regulatory subunit 3 (gamma)	2.4	*ns*	3.0
EAF2	ELL associated factor 2	2.9	*ns*	2.8
ZEB2	zinc finger E-box binding homeobox 2	2.8	*ns*	2.2
GLUL	glutamate-ammonia ligase	2.8	*ns*	2.7
ATP8A1	ATPase, aminophospholipid transporter (APLT), class I, type 8A, member 1	2.6	*ns*	2.6
EFEMP1	EGF containing fibulin-like extracellular matrix protein 1	2.6	*ns*	2.4
SSBP2	single-stranded DNA binding protein 2	2.5	*ns*	2.2
ARHGEF26	Rho guanine nucleotide exchange factor 26	2.4	*ns*	2.5
C1QTNF1	C1q and tumor necrosis factor related protein 1	2.2	*ns*	2.5
TEC	tec protein tyrosine kinase	2.3	*ns*	2.5
DEFB109B	defensin beta 109B	2.5	*ns*	2.2
RESF1	retroelement silencing factor 1	2.4	*ns*	2.1
PLCG2	phospholipase C, gamma 2 (phosphatidylinositol-specific)	2.2	*ns*	2.4
FLI1	Fli-1 proto-oncogene, ETS transcription factor	2.1	*ns*	2.4
PCDH17	protocadherin 17	2.3	*ns*	2.4
PTPRJ	protein tyrosine phosphatase, receptor type, J	2.2	*ns*	2.3
LOC105375977	uncharacterized LOC105375977	2.3	*ns*	2.1
SNORD114-2	small nucleolar RNA, C	*ns*	22.1	*ns*
SNORD114-6	small nucleolar RNA, C	*ns*	20.9	*ns*
SNORD114-7	small nucleolar RNA, C	*ns*	20.5	*ns*
SNORD114-19	small nucleolar RNA, C	*ns*	16.8	*ns*
SNORD114-12	small nucleolar RNA, C	*ns*	14.3	*ns*
ERVV-2	endogenous retrovirus group V, member 2	*ns*	14.1	*ns*
CD36	CD36 molecule (thrombospondin receptor)	*ns*	13.4	*ns*
ZNF117	zinc finger protein 117	*ns*	13.3	*ns*
SNORD114-15	small nucleolar RNA, C	*ns*	13.2	*ns*
PABPC4L	poly(A) binding protein, cytoplasmic 4-like	*ns*	13.0	*ns*
SNORD114-13	small nucleolar RNA, C	*ns*	12.2	*ns*
SNORD114-17	small nucleolar RNA, C	*ns*	12.2	*ns*
SNORD114-11	small nucleolar RNA, C	*ns*	11.6	*ns*
ERVV-1	endogenous retrovirus group V, member 1	*ns*	10.7	*ns*
SNORD113-3	small nucleolar RNA, C	*ns*	10.7	*ns*
MIR503	microRNA 503	*ns*	10.2	*ns*
PCDH18	protocadherin 18	10.2	*ns*	*ns*
PLAC1	placenta specific 1	*ns*	9.8	*ns*
LOC105378624	uncharacterized LOC105378624	*ns*	9.5	*ns*
SNORD114-30	small nucleolar RNA, C	*ns*	9.4	*ns*
SNORD113-2	small nucleolar RNA, C	*ns*	9.0	*ns*
MIR1305	microRNA 1305	*ns*	8.9	*ns*
LINC01483	long intergenic non-protein coding RNA 1483	*ns*	8.7	*ns*
SNORD114-3	small nucleolar RNA, C	*ns*	8.7	*ns*
SNORD114-27	small nucleolar RNA, C	*ns*	8.5	*ns*
SNORD113-9	small nucleolar RNA, C	*ns*	8.2	*ns*
LOC105376127	uncharacterized LOC105376127	*ns*	7.9	*ns*
SNORD114-21	small nucleolar RNA, C	*ns*	7.8	*ns*
PCDH11Y	protocadherin 11 Y-linked	*ns*	7.4	*ns*
SNORD59A	small nucleolar RNA, C	*ns*	7.3	*ns*
SNORD114-1	small nucleolar RNA, C	*ns*	7.2	*ns*
PSG6	pregnancy specific beta-1-glycoprotein 6	*ns*	7.1	*ns*
CHODL-AS1	CHODL antisense RNA 1	*ns*	7.1	*ns*
PSG11	pregnancy specific beta-1-glycoprotein 11	*ns*	6.9	*ns*
TCF21	transcription factor 21	*ns*	6.8	*ns*
SCARNA2	small Cajal body-specific RNA 2	*ns*	6.5	*ns*

Development • Supplementary information

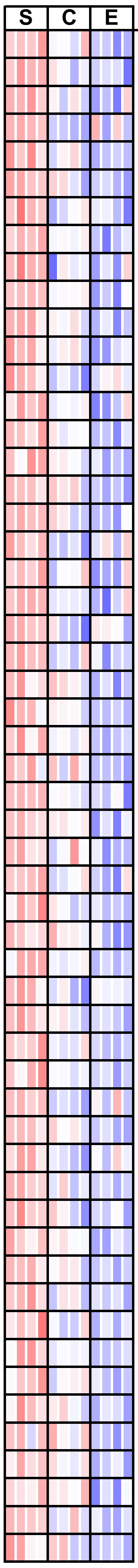

Symbol	Name	Linear Fold Change (*ns*=no significant difference) S vs C	S vs E	E vs C
ARHGAP26-IT1	ARHGAP26 intronic transcript 1	*ns*	6.4	*ns*
SNORD114-22	small nucleolar RNA, C	*ns*	6.4	*ns*
OLR1	oxidized low density lipoprotein (lectin-like) receptor 1	*ns*	6.3	*ns*
ADGRL2	adhesion G protein-coupled receptor L2	6.2	*ns*	*ns*
LOC105375401	uncharacterized LOC105375401	*ns*	6.2	*ns*
LOC101927745	uncharacterized LOC101927745	*ns*	6.2	*ns*
MIR4659B	microRNA 4659b	*ns*	6.2	*ns*
CLIP4	CAP-GLY domain containing linker protein family, member 4	*ns*	6.1	*ns*
PLCE1-AS1	PLCE1 antisense RNA 1	*ns*	6.0	*ns*
MORC4	MORC family CW-type zinc finger 4	*ns*	6.0	*ns*
L1TD1	LINE-1 type transposase domain containing 1	*ns*	5.9	*ns*
LOC101929607	uncharacterized LOC101929607	*ns*	5.9	*ns*
GNG11	guanine nucleotide binding protein (G protein), gamma 11	5.8	*ns*	*ns*
LOC105370804	uncharacterized LOC105370804	*ns*	5.8	*ns*
LOC220729	succinate dehydrogenase complex, subunit A, flavoprotein (Fp) pseudogene	*ns*	5.7	*ns*
CASC9	cancer susceptibility candidate 9 (non-protein coding)	*ns*	5.7	*ns*
HELLS	helicase, lymphoid-specific	*ns*	5.6	*ns*
SLIT2	slit guidance ligand 2	*ns*	5.6	*ns*
TLL1	tolloid like 1	5.5	*ns*	*ns*
RAB3B	RAB3B, member RAS oncogene family	*ns*	5.5	*ns*
FBN2	fibrillin 2	*ns*	5.5	*ns*
ENPEP	glutamyl aminopeptidase (aminopeptidase A)	5.5	*ns*	*ns*
LOC101927685	heat shock transcription factor, X-linked-like	*ns*	5.5	*ns*
LOC100508631	uncharacterized LOC100508631	*ns*	5.4	*ns*
MAEL	maelstrom spermatogenic transposon silencer	*ns*	5.3	*ns*
SIK3-IT1	SIK3 intronic transcript 1	*ns*	5.2	*ns*
SHANK2	SH3 and multiple ankyrin repeat domains 2	*ns*	5.2	*ns*
ACP3	acid phosphatase 3	*ns*	5.2	*ns*
NRK	Nik related kinase	*ns*	5.2	*ns*
LOC102725247	uncharacterized LOC102725247	*ns*	5.1	*ns*
CD200	CD200 molecule	*ns*	5.1	*ns*
MEG8	maternally expressed 8 (non-protein coding)	*ns*	5.1	*ns*
SNORD13P2	small nucleolar RNA, C	*ns*	5.0	*ns*
AKR1B15	aldo-keto reductase family 1, member B15	*ns*	4.9	*ns*
LRCH2	leucine-rich repeats and calponin homology (CH) domain containing 2	4.9	*ns*	*ns*
SLC16A10	solute carrier family 16 (aromatic amino acid transporter), member 10	*ns*	4.9	*ns*
DLG5	discs, large homolog 5 (Drosophila)	*ns*	4.9	*ns*
DHRS2	dehydrogenase	*ns*	4.9	*ns*
GUCY1A2	guanylate cyclase 1, soluble, alpha 2	4.8	*ns*	*ns*
NFE2L3	nuclear factor, erythroid 2-like 3	*ns*	4.8	*ns*
SNORD116-21	small nucleolar RNA, C	4.7	*ns*	*ns*
MIRLET7F1	microRNA let-7f-1	*ns*	4.7	*ns*
SNORD113-7	small nucleolar RNA, C	*ns*	4.7	*ns*
LOC105376236	uncharacterized LOC105376236	*ns*	4.6	*ns*
RASSF6	Ras association (RalGDS	*ns*	4.6	*ns*
S100A9	S100 calcium binding protein A9	*ns*	4.6	*ns*
BDP1	B double prime 1, subunit of RNA polymerase III transcription initiation factor IIIB	*ns*	4.6	*ns*
PSG4	pregnancy specific beta-1-glycoprotein 4	*ns*	4.6	*ns*
OR52E6	olfactory receptor, family 52, subfamily E, member 6	*ns*	4.5	*ns*
HAPLN1	hyaluronan and proteoglycan link protein 1	*ns*	4.4	*ns*
LOC105377649	uncharacterized LOC105377649	*ns*	4.4	*ns*
LOC105371225	uncharacterized LOC105371225	*ns*	4.4	*ns*
LIPG	lipase, endothelial	*ns*	4.4	*ns*
GH1	growth hormone 1	*ns*	4.4	*ns*
SNORD114-8	small nucleolar RNA, C	*ns*	4.4	*ns*

Development • Supplementary information

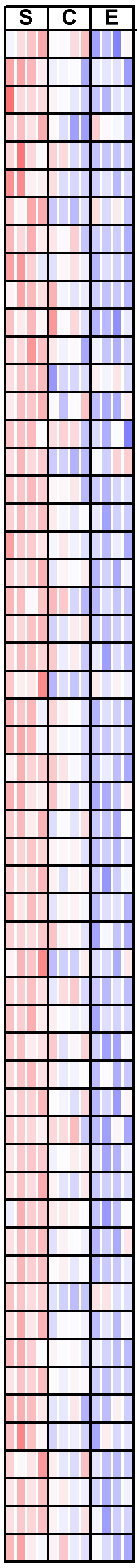

Symbol	Name	Linear Fold Change (*ns*=no significant difference) S vs C	S vs E	E vs C
OR7E91P	olfactory receptor, family 7, subfamily E, member 91 pseudogene	*ns*	4.3	*ns*
CCDC68	coiled-coil domain containing 68	*ns*	4.3	*ns*
XDH	xanthine dehydrogenase	*ns*	4.3	*ns*
COL6A3	collagen, type VI, alpha 3	4.3	*ns*	*ns*
LOC101927359	uncharacterized LOC101927359	*ns*	4.3	*ns*
LOC644919	uncharacterized LOC644919	*ns*	4.3	*ns*
NDNF	neuron-derived neurotrophic factor	4.2	*ns*	*ns*
TUSC3	tumor suppressor candidate 3	*ns*	4.2	*ns*
LOC105377196	uncharacterized LOC105377196	*ns*	4.2	*ns*
PDE3B	phosphodiesterase 3B, cGMP-inhibited	*ns*	4.2	*ns*
LOC101928794	uncharacterized LOC101928794	*ns*	4.2	*ns*
CXADRP2	coxsackie virus and adenovirus receptor pseudogene 2	*ns*	4.2	*ns*
ITGB3	integrin beta 3	4.2	*ns*	*ns*
MIR29C	microRNA 29c	*ns*	4.2	*ns*
CDS1	CDP-diacylglycerol synthase 1	*ns*	4.2	*ns*
GUCY1A1	guanylate cyclase 1 soluble subunit alpha 1	4.2	*ns*	*ns*
MAP3K13	mitogen-activated protein kinase kinase kinase 13	*ns*	4.1	*ns*
DAB2	Dab, mitogen-responsive phosphoprotein, homolog 2 (Drosophila)	*ns*	4.1	*ns*
NAALADL2-AS3	NAALADL2 antisense RNA 3	*ns*	4.1	*ns*
PSG2	pregnancy specific beta-1-glycoprotein 2	*ns*	4.0	*ns*
LOC102723920	uncharacterized LOC102723920	*ns*	4.0	*ns*
SLC30A2	solute carrier family 30 (zinc transporter), member 2	*ns*	4.0	*ns*
CKAP2L	cytoskeleton associated protein 2-like	*ns*	4.0	*ns*
FCGR2B	Fc fragment of IgG, low affinity IIb, receptor (CD32)	3.9	*ns*	*ns*
MIR3650	microRNA 3650	*ns*	3.9	*ns*
ZNF354B	zinc finger protein 354B	*ns*	3.9	*ns*
MIR17HG	miR-17-92 cluster host gene	*ns*	3.9	*ns*
LINC00622	long intergenic non-protein coding RNA 622	*ns*	3.9	*ns*
LOC100507487	uncharacterized LOC100507487	*ns*	3.9	*ns*
RHOBTB3	Rho-related BTB domain containing 3	*ns*	3.9	*ns*
LOC102723908	uncharacterized LOC102723908	*ns*	3.9	*ns*
PSCA	prostate stem cell antigen	*ns*	3.9	*ns*
C2orf81	chromosome 2 open reading frame 81	*ns*	3.8	*ns*
MGC12916	uncharacterized protein MGC12916	*ns*	3.7	*ns*
LOC105378300	uncharacterized LOC105378300	*ns*	3.8	*ns*
TCHHL1	trichohyalin like 1	*ns*	3.8	*ns*
LOC105369897	uncharacterized LOC105369897	*ns*	3.8	*ns*
IL1RAPL2	interleukin 1 receptor accessory protein-like 2	*ns*	3.7	*ns*
AGO2	argonaute RISC catalytic component 2	*ns*	3.7	*ns*
PBK	PDZ binding kinase	*ns*	3.7	*ns*
EMSY	EMSY BRCA2-interacting transcriptional repressor	*ns*	3.7	*ns*
SDHAP2	succinate dehydrogenase complex subunit A, flavoprotein pseudogene 2	*ns*	3.7	*ns*
PLGLB1	plasminogen-like B1	*ns*	3.7	*ns*
SEMA6D	semaphorin 6D	*ns*	3.7	*ns*
TMEM247	transmembrane protein 247	*ns*	3.7	*ns*
ZNF521	zinc finger protein 521	3.6	*ns*	*ns*
DUBR	DPPA2 upstream binding RNA	*ns*	3.6	*ns*
NAALADL2	N-acetylated alpha-linked acidic dipeptidase-like 2	*ns*	3.6	*ns*
TAB3	TGF-beta activated kinase 1	*ns*	3.6	*ns*
LRRN3	leucine rich repeat neuronal 3	*ns*	3.6	*ns*
SLC6A15	solute carrier family 6 (neutral amino acid transporter), member 15	*ns*	3.6	*ns*
GTF2IP20	general transcription factor IIi pseudogene 20	*ns*	3.5	*ns*
RBMS3	RNA binding motif, single stranded interacting protein 3	*ns*	3.5	*ns*
PDZD2	PDZ domain containing 2	*ns*	3.5	*ns*
LOC100134868	uncharacterized LOC100134868	*ns*	3.5	*ns*

Development • Supplementary information

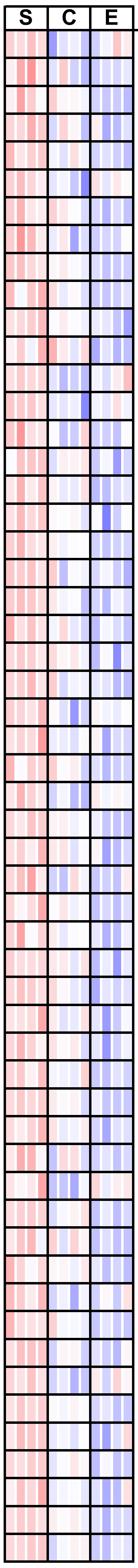

Symbol	Name	Linear Fold Change (*ns*=no significant difference) S vs C	S vs E	E vs C
ADIRF	adipogenesis regulatory factor	3.5	*ns*	*ns*
MIR487B	microRNA 487b	*ns*	3.5	*ns*
LOC102723439	uncharacterized LOC102723439	*ns*	3.5	*ns*
SNORA38B	small nucleolar RNA, H	*ns*	3.5	*ns*
KCNK5	potassium channel, two pore domain subfamily K, member 5	*ns*	3.5	*ns*
OGFRL1	opioid growth factor receptor-like 1	3.4	*ns*	*ns*
TFRC	transferrin receptor	*ns*	3.4	*ns*
SCN9A	sodium channel, voltage gated, type IX alpha subunit	*ns*	3.4	*ns*
LONP2	lon peptidase 2, peroxisomal	*ns*	3.4	*ns*
ATG9B	autophagy related 9B	*ns*	3.4	*ns*
SERPINF1	serpin peptidase inhibitor F1	*ns*	3.4	*ns*
MIRLET7G	microRNA let-7g	*ns*	3.4	*ns*
RAPGEF5	Rap guanine nucleotide exchange factor 5	3.4	*ns*	*ns*
EXOC6	exocyst complex component 6	3.4	*ns*	*ns*
CARD17	caspase recruitment domain family, member 17	*ns*	3.4	*ns*
FBXO9	F-box protein 9	*ns*	3.4	*ns*
MTNR1B	melatonin receptor 1B	*ns*	3.4	*ns*
LOC389765	kinesin family member 27 pseudogene	*ns*	3.4	*ns*
ZNF83	zinc finger protein 83	*ns*	3.3	*ns*
MIR581	microRNA 581	*ns*	3.3	*ns*
ARL15	ADP-ribosylation factor like GTPase 15	*ns*	3.3	*ns*
LINC01091	long intergenic non-protein coding RNA 1091	*ns*	3.3	*ns*
LOC102724917	uncharacterized LOC102724917	*ns*	3.3	*ns*
SDHAP1	succinate dehydrogenase complex subunit A, flavoprotein pseudogene 1	*ns*	3.3	*ns*
NFIB	nuclear factor I	3.3	*ns*	*ns*
RIMKLB	ribosomal modification protein rimK-like family member B	*ns*	3.3	*ns*
LOC101929787	uncharacterized LOC101929787	*ns*	3.3	*ns*
DUSP6	dual specificity phosphatase 6	3.3	*ns*	*ns*
RNF43	ring finger protein 43	*ns*	3.3	*ns*
LOC100133331	uncharacterized LOC100133331	*ns*	3.3	*ns*
XKRX	X-linked Kx blood group related, X-linked	*ns*	3.3	*ns*
LOC101926892	uncharacterized LOC101926892	*ns*	3.3	*ns*
LOC101928324	uncharacterized LOC101928324	*ns*	3.3	*ns*
CYTH3	cytohesin 3	*ns*	3.3	*ns*
ITGBL1	integrin beta like 1	*ns*	3.2	*ns*
NPIPB4	nuclear pore complex interacting protein family, member B4	*ns*	3.2	*ns*
LINC01355	long intergenic non-protein coding RNA 1355	*ns*	3.2	*ns*
CLMN	calmin (calponin-like, transmembrane)	*ns*	3.2	*ns*
ZFP2	ZFP2 zinc finger protein	*ns*	3.2	*ns*
DVL3	dishevelled segment polarity protein 3	*ns*	3.2	*ns*
NIPAL1	NIPA-like domain containing 1	*ns*	3.2	*ns*
WARS1	tryptophanyl-tRNA synthetase 1	3.2	*ns*	*ns*
TTBK2	tau tubulin kinase 2	*ns*	3.2	*ns*
SYCP2L	synaptonemal complex protein 2-like	*ns*	3.2	*ns*
SNAR-G1	small ILF3	*ns*	3.2	*ns*
HBA2	hemoglobin, alpha 2	3.2	*ns*	*ns*
LINC00939	long intergenic non-protein coding RNA 939	*ns*	3.2	*ns*
MMD	monocyte to macrophage differentiation-associated	*ns*	3.2	*ns*
CYYR1	cysteine	3.2	*ns*	*ns*
CLIP1-AS1	CLIP1 antisense RNA 1	*ns*	3.2	*ns*
SCIN	scinderin	*ns*	3.2	*ns*
ST3GAL4	ST3 beta-galactoside alpha-2,3-sialyltransferase 4	*ns*	3.1	*ns*
ELMO1-AS1	ELMO1 antisense RNA 1	*ns*	3.1	*ns*
RGPD6	RANBP2-like and GRIP domain containing 6	*ns*	3.1	*ns*
IKZF2	IKAROS family zinc finger 2	*ns*	3.1	*ns*

Development • Supplementary information

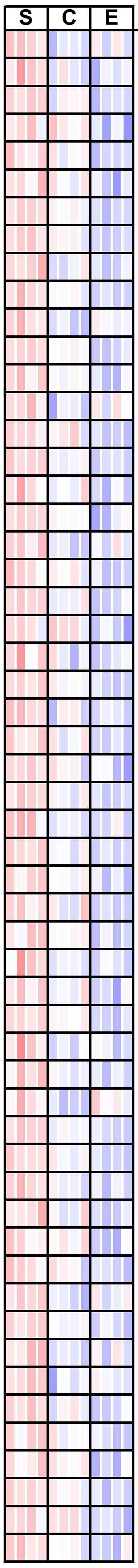

Symbol	Name	Linear Fold Change (*ns*=no significant difference) S vs C	S vs E	E vs C
SLC45A4	solute carrier family 45, member 4	3.1	*ns*	*ns*
FAM124B	family with sequence similarity 124 member B	*ns*	3.1	*ns*
BEX2	brain expressed X-linked 2	*ns*	3.1	*ns*
OCLN	occludin	*ns*	3.1	*ns*
SPIRE2	spire-type actin nucleation factor 2	*ns*	3.1	*ns*
LOC101928344	protein GVQW1-like	*ns*	3.1	*ns*
RGPD8	RANBP2-like and GRIP domain containing 8	*ns*	3.1	*ns*
SYNJ1	synaptojanin 1	*ns*	3.1	*ns*
DHCR24	24-dehydrocholesterol reductase	*ns*	3.1	*ns*
LOC100505938	uncharacterized LOC100505938	*ns*	3.1	*ns*
PLSCR4	phospholipid scramblase 4	3.1	*ns*	*ns*
C21orf91	chromosome 21 open reading frame 91	*ns*	3.1	*ns*
LRRTM2	leucine rich repeat transmembrane neuronal 2	*ns*	3.1	*ns*
GPNMB	glycoprotein (transmembrane) nmb	3.1	*ns*	*ns*
CCNE2	cyclin E2	*ns*	3.0	*ns*
SH3GLB2	SH3-domain GRB2-like endophilin B2	*ns*	3.0	*ns*
DHRSX	dehydrogenase	*ns*	3.0	*ns*
LOC101929494	uncharacterized LOC101929494	*ns*	3.0	*ns*
GJC1	gap junction protein gamma 1	3.0	*ns*	*ns*
ORC6	origin recognition complex subunit 6	*ns*	3.0	*ns*
STX3	syntaxin 3	*ns*	3.0	*ns*
CCNB2	cyclin B2	*ns*	2.9	*ns*
SLIT2-IT1	SLIT2 intronic transcript 1	*ns*	3.0	*ns*
CEP152	centrosomal protein 152kDa	*ns*	3.0	*ns*
SH3GL1P2	SH3-domain GRB2-like 1 pseudogene 2	*ns*	3.0	*ns*
LOC105371471	uncharacterized LOC105371471	*ns*	3.0	*ns*
SPDYE6	speedy	*ns*	3.0	*ns*
LOC105374911	uncharacterized LOC105374911	*ns*	3.0	*ns*
MIR223	microRNA 223	*ns*	3.0	*ns*
CSGALNACT1	chondroitin sulfate N-acetylgalactosaminyltransferase 1	*ns*	3.0	*ns*
PAQR7	progestin and adipoQ receptor family member VII	*ns*	3.0	*ns*
CGB7	chorionic gonadotropin, beta polypeptide 7	*ns*	2.9	*ns*
ADGRG1	adhesion G protein-coupled receptor G1	*ns*	2.9	*ns*
MIR218-1	microRNA 218-1	*ns*	2.9	*ns*
ZNF250	zinc finger protein 250	*ns*	2.9	*ns*
AMMECR1	Alport syndrome, mental retardation, midface hypoplasia and elliptocytosis chromosomal region gene 1	*ns*	2.9	*ns*
LOC101930100	uncharacterized LOC101930100	*ns*	2.9	*ns*
LOC100133091	uncharacterized LOC100133091	*ns*	2.9	*ns*
LRIG3	leucine-rich repeats and immunoglobulin-like domains 3	2.9	*ns*	*ns*
LOC101929709	uncharacterized LOC101929709	*ns*	2.9	*ns*
EPHB4	EPH receptor B4	*ns*	2.9	*ns*
ANKRD20A2P	ankyrin repeat domain 20 family member A2, pseudogene	*ns*	2.9	*ns*
TRIM25	tripartite motif containing 25	*ns*	2.9	*ns*
PDXDC2P	pyridoxal-dependent decarboxylase domain containing 2, pseudogene	*ns*	2.9	*ns*
GGCT	gamma-glutamylcyclotransferase	*ns*	2.9	*ns*
LOC105369204	uncharacterized LOC105369204	2.9	*ns*	*ns*
ACADVL	acyl-CoA dehydrogenase, very long chain	*ns*	2.9	*ns*
BTG2	BTG family, member 2	2.9	*ns*	*ns*
BCL2	B-cell CLL	2.9	*ns*	*ns*
AUNIP	aurora kinase A and ninein interacting protein	*ns*	2.9	*ns*
SH2D4A	SH2 domain containing 4A	*ns*	2.9	*ns*
LIN28B	lin-28 homolog B (C. elegans)	*ns*	2.9	*ns*
MCM6	minichromosome maintenance complex component 6	*ns*	2.9	*ns*
AGL	amylo-alpha-1, 6-glucosidase, 4-alpha-glucanotransferase	*ns*	2.9	*ns*
ARRDC3-AS1	ARRDC3 antisense RNA 1	*ns*	2.9	*ns*

Development • Supplementary information

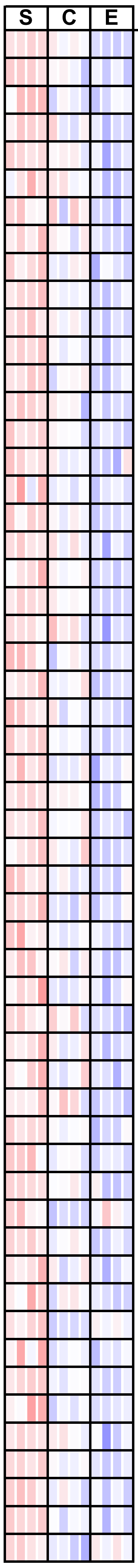

Symbol	Name	Linear Fold Change (*ns*=no significant difference) S vs C	S vs E	E vs C
TOMM34	translocase of outer mitochondrial membrane 34	*ns*	2.9	*ns*
CNN3	calponin 3, acidic	*ns*	2.9	*ns*
VSTM5	V-set and transmembrane domain containing 5	*ns*	2.8	*ns*
LOC100506606	uncharacterized LOC100506606	*ns*	2.8	*ns*
RERG-AS1	RERG antisense RNA 1	*ns*	2.8	*ns*
LOC105376073	uncharacterized LOC105376073	*ns*	2.8	*ns*
MIR521-1	microRNA 521-1	*ns*	2.8	*ns*
LOC101930131	uncharacterized LOC101930131	*ns*	2.8	*ns*
PGF	placental growth factor	*ns*	2.8	*ns*
NUFIP2	nuclear fragile X mental retardation protein interacting protein 2	*ns*	2.8	*ns*
SECISBP2L	SECIS binding protein 2-like	*ns*	2.8	*ns*
MAP3K4	mitogen-activated protein kinase kinase kinase 4	*ns*	2.8	*ns*
AKTIP	AKT interacting protein	*ns*	2.8	*ns*
ZC2HC1A	zinc finger, C2HC-type containing 1A	*ns*	2.8	*ns*
RPIA	ribose 5-phosphate isomerase A	*ns*	2.8	*ns*
PSTPIP2	proline-serine-threonine phosphatase interacting protein 2	*ns*	2.8	*ns*
HSD17B1P1	hydroxysteroid (17-beta) dehydrogenase 1 pseudogene 1	*ns*	2.8	*ns*
CRYBG2	crystallin beta-gamma domain containing 2	*ns*	2.8	*ns*
ADAM20	ADAM metallopeptidase domain 20	*ns*	2.8	*ns*
LOC105369468	uncharacterized LOC105369468	*ns*	2.8	*ns*
RGPD1	RANBP2-like and GRIP domain containing 1	*ns*	2.8	*ns*
NDC80	NDC80 kinetochore complex component	*ns*	2.8	*ns*
LOC101929570	uncharacterized LOC101929570	*ns*	2.8	*ns*
TTC7B	tetratricopeptide repeat domain 7B	*ns*	2.8	*ns*
LOC101928472	uncharacterized LOC101928472	*ns*	2.8	*ns*
ALDH7A1	aldehyde dehydrogenase 7 family, member A1	*ns*	2.8	*ns*
LOC105374895	uncharacterized LOC105374895	*ns*	2.8	*ns*
NEDD4	neural precursor cell expressed, developmentally down-regulated 4, E3 ubiquitin protein ligase	*ns*	2.8	*ns*
USP43	ubiquitin specific peptidase 43	*ns*	2.8	*ns*
EGFR	epidermal growth factor receptor	*ns*	2.8	*ns*
STARD4	StAR-related lipid transfer domain containing 4	*ns*	2.8	*ns*
PIK3C2B	phosphatidylinositol-4-phosphate 3-kinase, catalytic subunit type 2 beta	*ns*	2.7	*ns*
RPS6KA6	ribosomal protein S6 kinase, 90kDa, polypeptide 6	*ns*	2.7	*ns*
HERC2P4	hect domain and RLD 2 pseudogene 4	*ns*	2.7	*ns*
MRTFB	myocardin related transcription factor B	*ns*	2.7	*ns*
SHCBP1	SHC SH2-domain binding protein 1	*ns*	2.7	*ns*
NPIPB11	nuclear pore complex interacting protein family, member B11	*ns*	2.7	*ns*
BCORL1	BCL6 corepressor-like 1	*ns*	2.7	*ns*
SPATA9	spermatogenesis associated 9	*ns*	2.7	*ns*
RFX2	regulatory factor X, 2 (influences HLA class II expression)	*ns*	2.7	*ns*
MARVELD3	MARVEL domain containing 3	*ns*	2.7	*ns*
LOC101929461	uncharacterized LOC101929461	*ns*	2.7	*ns*
PCAT19	prostate cancer associated transcript 19 (non-protein coding)	2.7	*ns*	*ns*
ALDH4A1	aldehyde dehydrogenase 4 family, member A1	*ns*	2.7	*ns*
NPIPA1	nuclear pore complex interacting protein family member A1	*ns*	2.7	*ns*
LYPD5	LY6	*ns*	2.7	*ns*
TGM2	transglutaminase 2	2.7	*ns*	*ns*
OR6W1P	olfactory receptor, family 6, subfamily W, member 1 pseudogene	*ns*	2.7	*ns*
DCAF1	DDB1 and CUL4 associated factor 1	*ns*	2.7	*ns*
GAP43	growth associated protein 43	*ns*	2.7	*ns*
NAPB	N-ethylmaleimide-sensitive factor attachment protein, beta	*ns*	2.7	*ns*
ARGLU1	arginine and glutamate rich 1	*ns*	2.7	*ns*
CCL28	chemokine (C-C motif) ligand 28	*ns*	2.7	*ns*
LMO7	LIM domain 7	*ns*	2.7	*ns*
CAT	catalase	2.7	*ns*	*ns*

S	C	E	Symbol	Name	Linear Fold Change (*ns*=no significant difference) S vs C	S vs E	E vs C
			SOS2	SOS Ras	*ns*	2.7	*ns*
			ZNF443	zinc finger protein 443	*ns*	2.7	*ns*
			ZNRF3	zinc and ring finger 3	*ns*	2.7	*ns*
			ZNF525	zinc finger protein 525	*ns*	2.7	*ns*
			ECI2	enoyl-CoA delta isomerase 2	2.7	*ns*	*ns*
			HYMAI	hydatidiform mole associated and imprinted (non-protein coding)	*ns*	2.6	*ns*
			NEMP1	nuclear envelope integral membrane protein 1	*ns*	2.7	*ns*
			RGPD3	RANBP2-like and GRIP domain containing 3	*ns*	2.6	*ns*
			NPIPB3	nuclear pore complex interacting protein family, member B3	*ns*	2.6	*ns*
			NPIPB9	nuclear pore complex interacting protein family, member B9	*ns*	2.6	*ns*
			PCP4	Purkinje cell protein 4	*ns*	2.6	*ns*
			FILIP1	filamin A interacting protein 1	*ns*	2.6	*ns*
			ADAMTSL3	ADAMTS like 3	*ns*	2.6	*ns*
			PEAK1	pseudopodium-enriched atypical kinase 1	2.6	*ns*	*ns*
			H2BC21	H2B clustered histone 21	*ns*	2.6	*ns*
			ATXN7	ataxin 7	*ns*	2.6	*ns*
			PITX2	paired-like homeodomain 2	*ns*	2.6	*ns*
			IL36RN	interleukin 36 receptor antagonist	*ns*	2.6	*ns*
			CPPED1	calcineurin-like phosphoesterase domain containing 1	*ns*	2.6	*ns*
			GRIK1	glutamate receptor, ionotropic, kainate 1	*ns*	2.6	*ns*
			LARP4B	La ribonucleoprotein domain family, member 4B	*ns*	2.6	*ns*
			LOC105379161	uncharacterized LOC105379161	*ns*	2.6	*ns*
			DLG1	discs, large homolog 1 (Drosophila)	*ns*	2.6	*ns*
			NPIPB5	nuclear pore complex interacting protein family, member B5	*ns*	2.6	*ns*
			RMI2	RecQ mediated genome instability 2	*ns*	2.6	*ns*
			STEAP3	STEAP family member 3, metalloreductase	*ns*	2.6	*ns*
			CCDC125	coiled-coil domain containing 125	*ns*	2.6	*ns*
			PCP4L1	Purkinje cell protein 4 like 1	*ns*	2.6	*ns*
			TRIM33	tripartite motif containing 33	*ns*	2.6	*ns*
			MTMR1	myotubularin related protein 1	*ns*	2.6	*ns*
			RBM22	RNA binding motif protein 22	*ns*	2.6	*ns*
			PPP1R9A	protein phosphatase 1, regulatory subunit 9A	*ns*	2.6	*ns*
			SIK3	SIK family kinase 3	*ns*	2.6	*ns*
			RASGRP3	RAS guanyl releasing protein 3 (calcium and DAG-regulated)	2.6	*ns*	*ns*
			LINC00643	long intergenic non-protein coding RNA 643	*ns*	2.6	*ns*
			TFDP2	transcription factor Dp-2 (E2F dimerization partner 2)	*ns*	2.6	*ns*
			RBL1	retinoblastoma-like 1	*ns*	2.6	*ns*
			KCNN4	potassium channel, calcium activated intermediate	*ns*	2.6	*ns*
			LOC105374155	uncharacterized LOC105374155	*ns*	2.6	*ns*
			ABCA5	ATP binding cassette subfamily A member 5	*ns*	2.6	*ns*
			RBBP7	retinoblastoma binding protein 7	*ns*	2.6	*ns*
			EFNB2	ephrin-B2	2.6	*ns*	*ns*
			LINC01061	long intergenic non-protein coding RNA 1061	*ns*	2.6	*ns*
			SMAD7	SMAD family member 7	*ns*	2.6	*ns*
			LOC100288069	uncharacterized LOC100288069	*ns*	2.5	*ns*
			GPD1L	glycerol-3-phosphate dehydrogenase 1-like	*ns*	2.5	*ns*
			FAR2	fatty acyl-CoA reductase 2	*ns*	2.5	*ns*
			KCTD3	potassium channel tetramerization domain containing 3	*ns*	2.5	*ns*
			ACBD6	acyl-CoA binding domain containing 6	*ns*	2.5	*ns*
			SLAIN1	SLAIN motif family member 1	2.5	*ns*	*ns*
			TRIM55	tripartite motif containing 55	*ns*	2.5	*ns*
			BAGE2	B melanoma antigen family, member 2	*ns*	2.5	*ns*
			LOC100996442	uncharacterized LOC100996442	*ns*	2.5	*ns*
			MCUB	mitochondrial calcium uniporter dominant negative subunit beta	*ns*	2.5	*ns*
			UTRN	utrophin	2.5	*ns*	*ns*

Development • Supplementary information

Development: doi:10.1242/dev.199626: Supplementary information

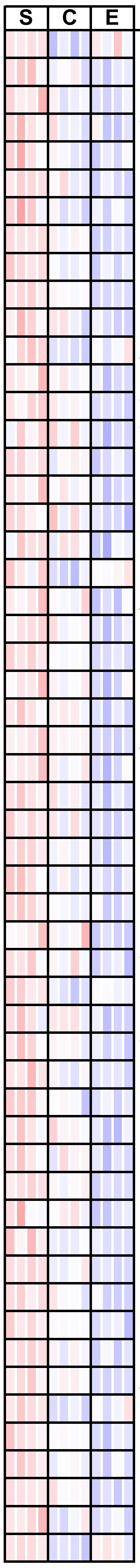

Symbol	Name	Linear Fold Change (*ns*=no significant difference) S vs C	S vs E	E vs C
RFTN2	raftlin family member 2	2.5	*ns*	*ns*
MTM1	myotubularin 1	*ns*	2.5	*ns*
ENOSF1	enolase superfamily member 1	*ns*	2.5	*ns*
TBC1D32	TBC1 domain family, member 32	*ns*	2.5	*ns*
KCTD4	potassium channel tetramerization domain containing 4	*ns*	2.5	*ns*
DIO3	deiodinase, iodothyronine, type III	*ns*	2.5	*ns*
GAS2L3	growth arrest-specific 2 like 3	*ns*	2.5	*ns*
MCM3	minichromosome maintenance complex component 3	*ns*	2.5	*ns*
DNMT3A	DNA (cytosine-5-)-methyltransferase 3 alpha	*ns*	2.5	*ns*
RGPD4	RANBP2-like and GRIP domain containing 4	*ns*	2.5	*ns*
FREM1	FRAS1 related extracellular matrix 1	*ns*	2.5	*ns*
ZNF22-AS1	ZNF22 antisense RNA 1	2.5	*ns*	*ns*
SPDYE2B	speedy	*ns*	2.5	*ns*
TKT	transketolase	*ns*	2.5	*ns*
YTHDC1	YTH domain containing 1	*ns*	2.5	*ns*
SLC2A11	solute carrier family 2 (facilitated glucose transporter), member 11	*ns*	2.5	*ns*
SPDYE2	speedy	*ns*	2.5	*ns*
GINS1	GINS complex subunit 1 (Psf1 homolog)	*ns*	2.5	*ns*
LOC100506514	uncharacterized LOC100506514	*ns*	2.5	*ns*
CAVIN2	caveolae associated protein 2	2.5	*ns*	*ns*
AMOTL1	angiomotin like 1	*ns*	2.5	*ns*
MDM4	MDM4, p53 regulator	*ns*	2.4	*ns*
SMS	spermine synthase	*ns*	2.4	*ns*
SMG1P2	SMG1 pseudogene 2	*ns*	2.4	*ns*
LARP1B	La ribonucleoprotein domain family, member 1B	*ns*	2.4	*ns*
AKR1B1	aldo-keto reductase family 1, member B1 (aldose reductase)	*ns*	2.4	*ns*
SMG1P3	SMG1 pseudogene 3	*ns*	2.4	*ns*
PAXBP1	PAX3 and PAX7 binding protein 1	*ns*	2.4	*ns*
LOC100128242	uncharacterized LOC100128242	*ns*	2.4	*ns*
PTPRK	protein tyrosine phosphatase, receptor type, K	*ns*	2.4	*ns*
LOC401585	uncharacterized LOC401585	*ns*	2.4	*ns*
HMGN2	high mobility group nucleosomal binding domain 2	*ns*	2.4	*ns*
GLI3	GLI family zinc finger 3	*ns*	2.4	*ns*
TAF4B	TAF4b RNA polymerase II, TATA box binding protein (TBP)-associated factor, 105kDa	*ns*	2.4	*ns*
NR2F1-AS1	NR2F1 antisense RNA 1	2.4	*ns*	*ns*
DIAPH2-AS1	DIAPH2 antisense RNA 1	*ns*	2.4	*ns*
TCAM1P	testicular cell adhesion molecule 1, pseudogene	*ns*	2.4	*ns*
KCTD1	potassium channel tetramerization domain containing 1	*ns*	2.4	*ns*
EXTL2	exostosin-like glycosyltransferase 2	*ns*	2.4	*ns*
LOC105379272	putative ankyrin repeat domain-containing protein 20A12 pseudogene	*ns*	2.4	*ns*
KLHL5	kelch-like family member 5	*ns*	2.4	*ns*
HSPBAP1	HSPB (heat shock 27kDa) associated protein 1	*ns*	2.4	*ns*
SNORD51	small nucleolar RNA, C	*ns*	2.4	*ns*
LOC113230	uncharacterized protein LOC113230	*ns*	2.4	*ns*
CBX5	chromobox homolog 5	*ns*	2.4	*ns*
LINC01004	long intergenic non-protein coding RNA 1004	*ns*	2.4	*ns*
HEXIM1	hexamethylene bis-acetamide inducible 1	*ns*	2.4	*ns*
C4orf19	chromosome 4 open reading frame 19	*ns*	2.4	*ns*
ZNF507	zinc finger protein 507	*ns*	2.4	*ns*
ENTPD1	ectonucleoside triphosphate diphosphohydrolase 1	2.4	*ns*	*ns*
CENATAC	centrosomal AT-AC splicing factor	*ns*	2.4	*ns*
MYO9A	myosin IXA	*ns*	2.4	*ns*
SPAG5	sperm associated antigen 5	*ns*	2.4	*ns*
HOPX	HOP homeobox	*ns*	2.4	*ns*
STOM	stomatin	2.4	*ns*	*ns*

Development • Supplementary information

Development: doi:10.1242/dev.199626: Supplementary information

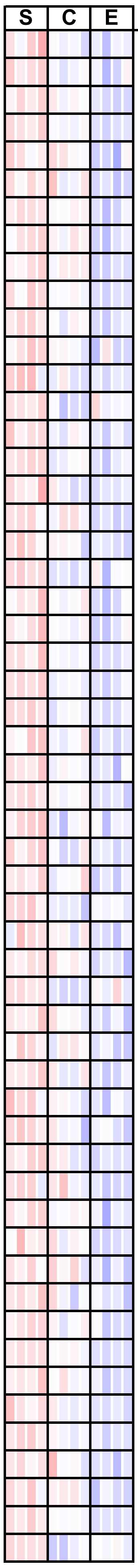

Symbol	Name	Linear Fold Change (*ns*=no significant difference) S vs C	S vs E	E vs C
TBX4	T-box 4	*ns*	2.4	*ns*
SPECC1L	sperm antigen with calponin homology and coiled-coil domains 1-like	*ns*	2.4	*ns*
MGST3	microsomal glutathione S-transferase 3	*ns*	2.4	*ns*
LOC553103	uncharacterized LOC553103	*ns*	2.4	*ns*
LOC400655	uncharacterized LOC400655	*ns*	2.4	*ns*
FER1L4	fer-1-like family member 4, pseudogene (functional)	*ns*	2.4	*ns*
MED13	mediator complex subunit 13	*ns*	2.4	*ns*
LOC105379520	uncharacterized LOC105379520	*ns*	2.4	*ns*
NRF1	nuclear respiratory factor 1	*ns*	2.4	*ns*
GCNT2	glucosaminyl (N-acetyl) transferase 2, I-branching enzyme (I blood group)	*ns*	2.4	*ns*
MTMR7	myotubularin related protein 7	*ns*	2.4	*ns*
TLCD5	TLC domain containing 5	*ns*	2.4	*ns*
LOC101927391	uncharacterized LOC101927391	*ns*	2.4	*ns*
SIRPA	signal-regulatory protein alpha	2.4	*ns*	*ns*
TP53TG3D	TP53 target 3D	*ns*	2.4	*ns*
EXOC2	exocyst complex component 2	*ns*	2.4	*ns*
JAM3	junctional adhesion molecule 3	*ns*	2.4	*ns*
N4BP2	NEDD4 binding protein 2	*ns*	2.4	*ns*
RANBP17	RAN binding protein 17	*ns*	2.4	*ns*
CPEB4	cytoplasmic polyadenylation element binding protein 4	2.4	*ns*	*ns*
SMG1P5	SMG1 pseudogene 5	*ns*	2.4	*ns*
MVB12B	multivesicular body subunit 12B	*ns*	2.4	*ns*
CEP68	centrosomal protein 68kDa	*ns*	2.4	*ns*
BAZ2B	bromodomain adjacent to zinc finger domain 2B	*ns*	2.4	*ns*
AHCYL2	adenosylhomocysteinase-like 2	*ns*	2.4	*ns*
MFSD2A	major facilitator superfamily domain containing 2A	*ns*	2.4	*ns*
FDFT1	farnesyl-diphosphate farnesyltransferase 1	*ns*	2.3	*ns*
PLEKHA7	pleckstrin homology domain containing, family A member 7	*ns*	2.3	*ns*
GABARAPL1	GABA(A) receptor-associated protein like 1	*ns*	2.3	*ns*
HID1	HID1 domain containing	*ns*	2.3	*ns*
DDB1	damage-specific DNA binding protein 1	*ns*	2.3	*ns*
FAM135A	family with sequence similarity 135, member A	2.3	*ns*	*ns*
TSL	testis-expressed, seven-twelve, leukemia	*ns*	2.3	*ns*
PAK6	p21 protein (Cdc42	*ns*	2.3	*ns*
ILDR1	immunoglobulin-like domain containing receptor 1	2.3	*ns*	*ns*
TMEM63A	transmembrane protein 63A	*ns*	2.3	*ns*
TULP4	tubby like protein 4	*ns*	2.3	*ns*
YAP1	Yes-associated protein 1	*ns*	2.3	*ns*
BBOX1	butyrobetaine (gamma), 2-oxoglutarate dioxygenase (gamma-butyrobetaine hydroxylase) 1	*ns*	2.3	*ns*
ZNF721	zinc finger protein 721	*ns*	2.3	*ns*
SH3D21	SH3 domain containing 21	*ns*	2.3	*ns*
CTHRC1	collagen triple helix repeat containing 1	*ns*	2.3	*ns*
LPCAT3	lysophosphatidylcholine acyltransferase 3	*ns*	2.3	*ns*
MIR548AL	microRNA 548al	*ns*	2.3	*ns*
CENPU	centromere protein U	*ns*	2.3	*ns*
LOC101059936	uncharacterized LOC101059936	*ns*	2.3	*ns*
LOC105374845	uncharacterized LOC105374845	*ns*	2.3	*ns*
ESRP2	epithelial splicing regulatory protein 2	*ns*	2.3	*ns*
SPDYE16	speedy	*ns*	2.3	*ns*
LENG8	leukocyte receptor cluster (LRC) member 8	*ns*	2.3	*ns*
LOC100289230	uncharacterized LOC100289230	*ns*	2.3	*ns*
LINC01036	long intergenic non-protein coding RNA 1036	*ns*	2.3	*ns*
TIGD2	tigger transposable element derived 2	*ns*	2.3	*ns*
BEX4	brain expressed X-linked 4	*ns*	2.3	*ns*
LSS	lanosterol synthase (2,3-oxidosqualene-lanosterol cyclase)	2.3	*ns*	*ns*

Development • Supplementary information

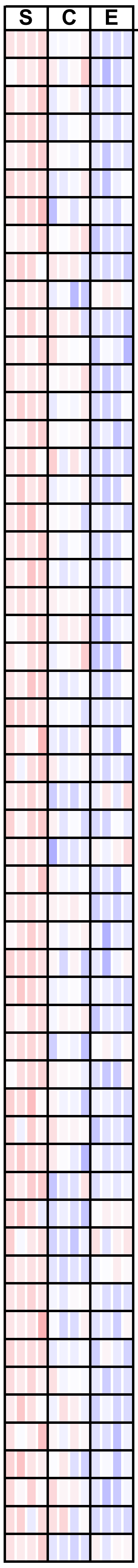

Symbol	Name	S vs C	S vs E	E vs C
		Linear Fold Change (*ns*=no significant difference)		
HP1BP3	heterochromatin protein 1, binding protein 3	*ns*	2.3	*ns*
KMT2C	lysine (K)-specific methyltransferase 2C	*ns*	2.3	*ns*
PITPNA	phosphatidylinositol transfer protein, alpha	*ns*	2.3	*ns*
NIN	ninein (GSK3B interacting protein)	*ns*	2.3	*ns*
MCM9	minichromosome maintenance 9 homologous recombination repair factor	*ns*	2.3	*ns*
DUS2	dihydrouridine synthase 2	*ns*	2.3	*ns*
STARD13	StAR-related lipid transfer domain containing 13	*ns*	2.3	*ns*
FLVCR2	feline leukemia virus subgroup C cellular receptor family, member 2	*ns*	2.3	*ns*
BRWD1	bromodomain and WD repeat domain containing 1	*ns*	2.3	*ns*
PMP22	peripheral myelin protein 22	2.3	*ns*	*ns*
RICTOR	RPTOR independent companion of MTOR, complex 2	*ns*	2.3	*ns*
WASH5P	WAS protein family homolog 5 pseudogene	*ns*	2.3	*ns*
MCM4	minichromosome maintenance complex component 4	*ns*	2.3	*ns*
SIAH2	siah E3 ubiquitin protein ligase 2	*ns*	2.3	*ns*
LOC100506314	uncharacterized LOC100506314	*ns*	2.3	*ns*
OMG	oligodendrocyte myelin glycoprotein	*ns*	2.3	*ns*
NFAT5	nuclear factor of activated T-cells 5, tonicity-responsive	*ns*	2.3	*ns*
ACER3	alkaline ceramidase 3	*ns*	2.3	*ns*
BRAF	B-Raf proto-oncogene, serine	*ns*	2.3	*ns*
GTF2IP4	general transcription factor IIi, pseudogene 4	*ns*	2.3	*ns*
TLK1	tousled-like kinase 1	*ns*	2.3	*ns*
CSRP2	cysteine and glycine-rich protein 2	*ns*	2.3	*ns*
PDE4DIP	phosphodiesterase 4D interacting protein	*ns*	2.2	*ns*
GTF2IP1	general transcription factor IIi pseudogene 1	*ns*	2.2	*ns*
HSPD1	heat shock 60kDa protein 1 (chaperonin)	*ns*	2.2	*ns*
RSKR	ribosomal protein S6 kinase related	*ns*	2.2	*ns*
GDPD5	glycerophosphodiester phosphodiesterase domain containing 5	*ns*	2.2	*ns*
PIR	pirin	2.2	*ns*	*ns*
BLMH	bleomycin hydrolase	*ns*	2.2	*ns*
PTPN9	protein tyrosine phosphatase, non-receptor type 9	2.2	*ns*	*ns*
WSB1	WD repeat and SOCS box containing 1	*ns*	2.2	*ns*
STK3	serine	*ns*	2.2	*ns*
SIPA1L1	signal-induced proliferation-associated 1 like 1	*ns*	2.2	*ns*
LOC102723575	uncharacterized LOC102723575	*ns*	2.2	*ns*
TMEM168	transmembrane protein 168	*ns*	2.2	*ns*
LINC00885	long intergenic non-protein coding RNA 885	*ns*	2.2	*ns*
PELI1	pellino E3 ubiquitin protein ligase 1	2.2	*ns*	*ns*
SLC35B4	solute carrier family 35 (UDP-xylose	*ns*	2.2	*ns*
CPT2	carnitine palmitoyltransferase 2	*ns*	2.2	*ns*
MYORG	myogenesis regulating glycosidase (putative)	*ns*	2.2	*ns*
DIPK2A	divergent protein kinase domain 2A	*ns*	2.2	*ns*
TMEM139	transmembrane protein 139	*ns*	2.2	*ns*
ARL4A	ADP-ribosylation factor like GTPase 4A	2.2	*ns*	*ns*
ABHD4	abhydrolase domain containing 4	2.2	*ns*	*ns*
GSR	glutathione reductase	2.2	*ns*	*ns*
CCDC171	coiled-coil domain containing 171	*ns*	2.2	*ns*
SMARCA2	SWI	*ns*	2.2	*ns*
LOC157860	uncharacterized LOC157860	*ns*	2.2	*ns*
TOP1	topoisomerase (DNA) I	*ns*	2.2	*ns*
LOC105374417	uncharacterized LOC105374417	*ns*	2.2	*ns*
MCM7	minichromosome maintenance complex component 7	*ns*	2.2	*ns*
LOC105377601	uncharacterized LOC105377601	*ns*	2.2	*ns*
ZNF468	zinc finger protein 468	*ns*	2.2	*ns*
ASB4	ankyrin repeat and SOCS box containing 4	*ns*	2.2	*ns*
MAGI1	membrane associated guanylate kinase, WW and PDZ domain containing 1	2.2	*ns*	*ns*

Development: doi:10.1242/dev.199626: Supplementary information

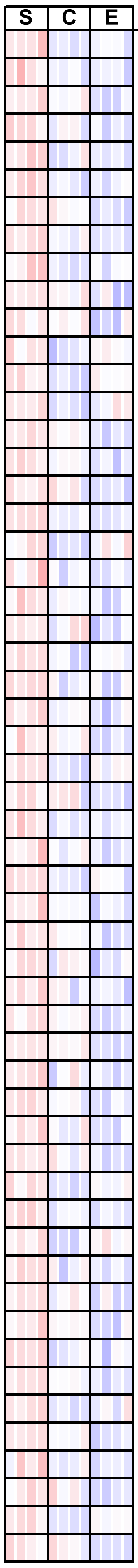

Symbol	Name	Linear Fold Change (*ns*=no significant difference) S vs C	S vs E	E vs C
EGFLAM	EGF-like, fibronectin type III and laminin G domains	2.2	*ns*	*ns*
COL28A1	collagen, type XXVIII, alpha 1	*ns*	2.2	*ns*
HNRNPD	heterogeneous nuclear ribonucleoprotein D	*ns*	2.2	*ns*
TAPT1	transmembrane anterior posterior transformation 1	*ns*	2.2	*ns*
OGDH	oxoglutarate (alpha-ketoglutarate) dehydrogenase (lipoamide)	*ns*	2.2	*ns*
TCAF1	TRPM8 channel-associated factor 1	2.2	*ns*	*ns*
LRATD1	LRAT domain containing 1	*ns*	2.2	*ns*
NIBAN2	niban apoptosis regulator 2	*ns*	2.2	*ns*
ACO1	aconitase 1, soluble	2.2	*ns*	*ns*
TEAD3	TEA domain family member 3	*ns*	2.2	*ns*
ZNF587B	zinc finger protein 587B	*ns*	2.2	*ns*
FUCA1	fucosidase, alpha-L- 1, tissue	2.2	*ns*	*ns*
SNRK	SNF related kinase	2.2	*ns*	*ns*
ERG	v-ets avian erythroblastosis virus E26 oncogene homolog	2.2	*ns*	*ns*
RDX	radixin	*ns*	2.2	*ns*
LOC105375137	uncharacterized LOC105375137	*ns*	2.2	*ns*
HMGB2	high mobility group box 2	*ns*	2.2	*ns*
SNX27	sorting nexin family member 27	*ns*	2.2	*ns*
EMP2	epithelial membrane protein 2	2.2	*ns*	*ns*
TARID	TCF21 antisense RNA inducing promoter demethylation	*ns*	2.2	*ns*
RMC1	regulator of MON1-CCZ1	*ns*	2.2	*ns*
MXD1	MAX dimerization protein 1	*ns*	2.2	*ns*
CCND2	cyclin D2	2.2	*ns*	*ns*
LINC00894	long intergenic non-protein coding RNA 894	*ns*	2.2	*ns*
PSMD11	proteasome 26S subunit, non-ATPase 11	*ns*	2.2	*ns*
MIR551B	microRNA 551b	*ns*	2.2	*ns*
SLC40A1	solute carrier family 40 (iron-regulated transporter), member 1	2.2	*ns*	*ns*
ETFA	electron-transfer-flavoprotein, alpha polypeptide	*ns*	2.2	*ns*
SCML1	sex comb on midleg-like 1 (Drosophila)	2.1	*ns*	*ns*
SRSF4	serine	*ns*	2.1	*ns*
PRKG1	protein kinase, cGMP-dependent, type I	2.1	*ns*	*ns*
NT5DC3	5-nucleotidase domain containing 3	*ns*	2.1	*ns*
ANKRD10	ankyrin repeat domain 10	*ns*	2.1	*ns*
SLC16A4	solute carrier family 16, member 4	*ns*	2.1	*ns*
LINC01347	long intergenic non-protein coding RNA 1347	*ns*	2.1	*ns*
ALAS1	5-aminolevulinate synthase 1	*ns*	2.1	*ns*
AAK1	AP2 associated kinase 1	*ns*	2.1	*ns*
NOX5	NADPH oxidase, EF-hand calcium binding domain 5	*ns*	2.1	*ns*
CNOT6L	CCR4-NOT transcription complex subunit 6-like	*ns*	2.1	*ns*
SETD5	SET domain containing 5	*ns*	2.1	*ns*
LOC100132287	uncharacterized LOC100132287	*ns*	2.1	*ns*
PDE4B	phosphodiesterase 4B, cAMP-specific	2.1	*ns*	*ns*
HES1	hes family bHLH transcription factor 1	*ns*	2.1	*ns*
NUDT3	nudix hydrolase 3	2.1	*ns*	*ns*
AMBRA1	autophagy	*ns*	2.1	*ns*
TAS2R60	taste receptor, type 2, member 60	*ns*	2.1	*ns*
TGFBR3	transforming growth factor beta receptor III	*ns*	2.1	*ns*
C1QTNF1-AS1	C1QTNF1 antisense RNA 1	*ns*	2.1	*ns*
GRAMD2B	GRAM domain containing 2B	*ns*	2.1	*ns*
FXN	frataxin	2.1	*ns*	*ns*
DEDD	death effector domain containing	*ns*	2.1	*ns*
AGAP1	ArfGAP with GTPase domain, ankyrin repeat and PH domain 1	*ns*	2.1	*ns*
ZNF552	zinc finger protein 552	*ns*	2.1	*ns*
MAP3K20	mitogen-activated protein kinase kinase kinase 20	2.1	*ns*	*ns*
FAM151B	family with sequence similarity 151, member B	*ns*	2.1	*ns*

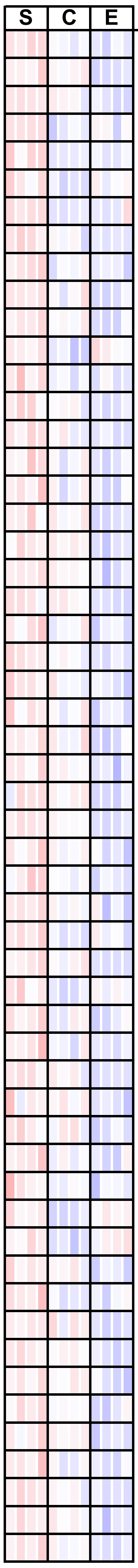

Symbol	Name	Linear Fold Change (*ns*=no significant difference) S vs C	S vs E	E vs C
KLHL42	kelch-like family member 42	*ns*	2.1	*ns*
NASP	nuclear autoantigenic sperm protein (histone-binding)	*ns*	2.1	*ns*
CBWD5	COBW domain containing 5	*ns*	2.1	*ns*
CPT1A	carnitine palmitoyltransferase 1A (liver)	2.1	*ns*	*ns*
PTPN1	protein tyrosine phosphatase, non-receptor type 1	2.1	*ns*	*ns*
VSTM4	V-set and transmembrane domain containing 4	2.1	*ns*	*ns*
EPB41L3	erythrocyte membrane protein band 4.1-like 3	2.1	*ns*	*ns*
MAP4K3	mitogen-activated protein kinase kinase kinase kinase 3	*ns*	2.1	*ns*
SQSTM1	sequestosome 1	*ns*	2.1	*ns*
MYO5B	myosin VB	*ns*	2.1	*ns*
TRA2B	transformer 2 beta homolog (Drosophila)	*ns*	2.1	*ns*
PSD3	pleckstrin and Sec7 domain containing 3	2.1	*ns*	*ns*
LOC100506504	uncharacterized LOC100506504	*ns*	2.1	*ns*
LRRC42	leucine rich repeat containing 42	*ns*	2.1	*ns*
NSG1	neuron specific gene family member 1	*ns*	2.1	*ns*
PGAP2	post-GPI attachment to proteins 2	*ns*	2.1	*ns*
NPIPA5	nuclear pore complex interacting protein family, member A5	*ns*	2.1	*ns*
MARVELD2	MARVEL domain containing 2	*ns*	2.1	*ns*
NUP58	nucleoporin 58kDa	*ns*	2.1	*ns*
HNRNPH1	heterogeneous nuclear ribonucleoprotein H1 (H)	*ns*	2.1	*ns*
MCM8	minichromosome maintenance 8 homologous recombination repair factor	*ns*	2.1	*ns*
PCDH1	protocadherin 1	*ns*	2.1	*ns*
SLC19A1	solute carrier family 19 (folate transporter), member 1	*ns*	2.1	*ns*
C1orf21	chromosome 1 open reading frame 21	*ns*	2.1	*ns*
EPS8L1	EPS8-like 1	*ns*	2.1	*ns*
CHD2	chromodomain helicase DNA binding protein 2	*ns*	2.1	*ns*
HMGN1	high mobility group nucleosome binding domain 1	*ns*	2.1	*ns*
LCOR	ligand dependent nuclear receptor corepressor	*ns*	2.1	*ns*
NF1	neurofibromin 1	*ns*	2.1	*ns*
STRA6	stimulated by retinoic acid 6	*ns*	2.1	*ns*
HMGCS2	3-hydroxy-3-methylglutaryl-CoA synthase 2 (mitochondrial)	*ns*	2.1	*ns*
FGFR2	fibroblast growth factor receptor 2	*ns*	2.1	*ns*
KIRREL1	kirre like nephrin family adhesion molecule 1	2.1	*ns*	*ns*
MED12	mediator complex subunit 12	*ns*	2.1	*ns*
ANKRD44-IT1	ANKRD44 intronic transcript 1	2.1	*ns*	*ns*
LOC105376363	uncharacterized LOC105376363	*ns*	2.1	*ns*
NXF1	nuclear RNA export factor 1	*ns*	2.0	*ns*
TNPO1	transportin 1	*ns*	2.0	*ns*
LRP5	LDL receptor related protein 5	*ns*	2.0	*ns*
TCTEX1D4	Tctex1 domain containing 4	*ns*	2.0	*ns*
FAN1	FANCD2	*ns*	2.0	*ns*
LOC100288911	uncharacterized LOC100288911	*ns*	2.0	*ns*
ABI2	abl-interactor 2	2.0	*ns*	*ns*
MANSC1	MANSC domain containing 1	2.0	*ns*	*ns*
HPDL	4-hydroxyphenylpyruvate dioxygenase-like	*ns*	2.0	*ns*
APBB2	amyloid beta (A4) precursor protein-binding, family B, member 2	2.0	*ns*	*ns*
PLEKHA8	pleckstrin homology domain containing, family A (phosphoinositide binding specific) member 8	*ns*	2.0	*ns*
SCARNA7	small Cajal body-specific RNA 7	*ns*	2.0	*ns*
EP300-AS1	EP300 antisense RNA 1	*ns*	2.0	*ns*
RASAL2	RAS protein activator like 2	*ns*	2.0	*ns*
HMGA1	high mobility group AT-hook 1	*ns*	2.0	*ns*
NPIPA7	nuclear pore complex interacting protein family, member A7	*ns*	2.0	*ns*
ARID2	AT rich interactive domain 2 (ARID, RFX-like)	*ns*	2.0	*ns*
RBM39	RNA binding motif protein 39	*ns*	2.0	*ns*
SFPQ	splicing factor proline	*ns*	2.0	*ns*

Development • Supplementary information

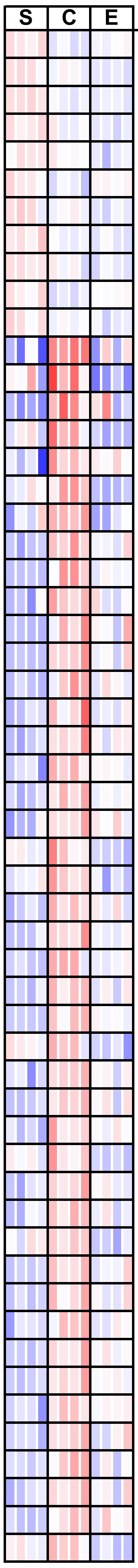

Symbol	Name	Linear Fold Change (*ns*=no significant difference) S vs C	S vs E	E vs C
LYPD6	LY6	2.0	*ns*	*ns*
EXOC6B	exocyst complex component 6B	*ns*	2.0	*ns*
DLG3	discs, large homolog 3 (Drosophila)	*ns*	2.0	*ns*
NEB	nebulin	*ns*	2.0	*ns*
AGFG1	ArfGAP with FG repeats 1	*ns*	2.0	*ns*
MOCS2	molybdenum cofactor synthesis 2	2.0	*ns*	*ns*
CASP3	caspase 3	*ns*	2.0	*ns*
LOC105370489	uncharacterized LOC105370489	*ns*	2.0	*ns*
KPNA2	karyopherin alpha 2 (RAG cohort 1, importin alpha 1)	*ns*	2.0	*ns*
HFE	hemochromatosis	2.0	*ns*	*ns*
CSTF2	cleavage stimulation factor, 3 pre-RNA, subunit 2	*ns*	2.0	*ns*
LEP	leptin	-19.5	*ns*	*ns*
OR5P2	olfactory receptor, family 5, subfamily P, member 2	*ns*	*ns*	-16.6
LOC105375837	uncharacterized LOC105375837	-12.4	*ns*	*ns*
LOC283299	uncharacterized LOC283299	*ns*	*ns*	-7.0
UCA1	urothelial cancer associated 1 (non-protein coding)	-6.4	*ns*	*ns*
LAMB4	laminin, beta 4	*ns*	*ns*	-6.4
AADACL2-AS1	AADACL2 antisense RNA 1	*ns*	*ns*	-5.8
ZFP42	ZFP42 zinc finger protein	-5.5	*ns*	*ns*
SLC17A8	solute carrier family 17 (vesicular glutamate transporter), member 8	-5.5	*ns*	*ns*
CA2	carbonic anhydrase II	-5.4	*ns*	*ns*
PPP4R4	protein phosphatase 4, regulatory subunit 4	-5.0	*ns*	*ns*
DOCK5	dedicator of cytokinesis 5	-5.0	*ns*	*ns*
ZNF300P1	zinc finger protein 300 pseudogene 1 (functional)	-4.9	*ns*	*ns*
LOC105370445	uncharacterized LOC105370445	-4.7	*ns*	*ns*
PLPP2	phospholipid phosphatase 2	-4.7	*ns*	*ns*
ARSK	arylsulfatase family, member K	-4.5	*ns*	*ns*
CNTNAP3	contactin associated protein-like 3	-4.4	*ns*	*ns*
FOS	FBJ murine osteosarcoma viral oncogene homolog	-4.3	*ns*	*ns*
LOC101927857	uncharacterized LOC101927857	*ns*	*ns*	-4.3
LOC102724484	uncharacterized LOC102724484	*ns*	*ns*	-4.2
LY6E	lymphocyte antigen 6 complex, locus E	-4.1	*ns*	*ns*
LOC100128979	uncharacterized LOC100128979	-4.0	*ns*	*ns*
HRAT17	heart tissue-associated transcript 17	-4.0	*ns*	*ns*
SFRP1	secreted frizzled-related protein 1	-4.0	*ns*	*ns*
BHLHE40	basic helix-loop-helix family, member e40	-4.0	*ns*	*ns*
TTK	TTK protein kinase	*ns*	*ns*	-3.9
NUAK2	NUAK family, SNF1-like kinase, 2	-3.9	*ns*	*ns*
BAIAP2L1	BAI1-associated protein 2-like 1	-3.8	*ns*	*ns*
LOC105372290	uncharacterized LOC105372290	-3.8	*ns*	*ns*
OR5E1P	olfactory receptor, family 5, subfamily E, member 1 pseudogene	*ns*	*ns*	-3.7
PRSS8	protease, serine, 8	-3.7	*ns*	*ns*
HCAR3	hydroxycarboxylic acid receptor 3	-3.7	*ns*	*ns*
SNORA3B	small nucleolar RNA, H	*ns*	*ns*	-3.7
LINC00294	long intergenic non-protein coding RNA 294	-3.7	*ns*	*ns*
EGLN3	egl-9 family hypoxia-inducible factor 3	-3.7	*ns*	*ns*
PGPEP1	pyroglutamyl-peptidase I	-3.6	*ns*	*ns*
SASH1	SAM and SH3 domain containing 1	-3.6	*ns*	*ns*
TET3	tet methylcytosine dioxygenase 3	-3.6	*ns*	*ns*
SLC35A3	solute carrier family 35 (UDP-N-acetylglucosamine transporter), member A3	-3.5	*ns*	*ns*
LINC00842	long intergenic non-protein coding RNA 842	-3.5	*ns*	*ns*
LOC102723446	uncharacterized LOC102723446	-3.5	*ns*	*ns*
F11R	F11 receptor	-3.5	*ns*	*ns*
NOS2	nitric oxide synthase 2, inducible	-3.5	*ns*	*ns*
COL21A1	collagen, type XXI, alpha 1	*ns*	*ns*	-3.5

Development • Supplementary information

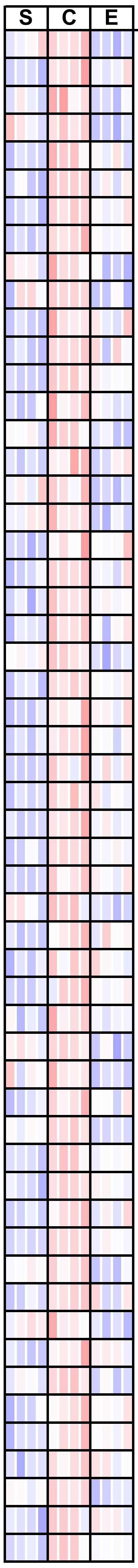

Symbol	Name	Linear Fold Change (*ns*=no significant difference) S vs C	S vs E	E vs C
ACKR2	atypical chemokine receptor 2	*ns*	*ns*	-3.5
SLC7A1	solute carrier family 7 (cationic amino acid transporter, y+ system), member 1	-3.4	*ns*	*ns*
LOC105379109	uncharacterized LOC105379109	*ns*	*ns*	-3.4
MIG7	mig-7	*ns*	*ns*	-3.4
TGFB1	transforming growth factor beta 1	-3.4	*ns*	*ns*
ANKRD50	ankyrin repeat domain 50	-3.3	*ns*	*ns*
DDX60L	DEAD (Asp-Glu-Ala-Asp) box polypeptide 60-like	-3.3	*ns*	*ns*
FLT1	fms-related tyrosine kinase 1	-3.3	*ns*	*ns*
ZNF726	zinc finger protein 726	*ns*	*ns*	-3.3
ADGRL3	adhesion G protein-coupled receptor L3	*ns*	*ns*	-3.3
LOC101927087	uncharacterized LOC101927087	-3.3	*ns*	*ns*
PLEKHA2	pleckstrin homology domain containing, family A (phosphoinositide binding specific) member 2	-3.2	*ns*	*ns*
MAN2A1	mannosidase, alpha, class 2A, member 1	-3.2	*ns*	*ns*
BIRC7	baculoviral IAP repeat containing 7	-3.2	*ns*	*ns*
DSC2	desmocollin 2	*ns*	*ns*	-3.2
SLC1A6	solute carrier family 1 (high affinity aspartate	-3.1	*ns*	*ns*
CYP8B1	cytochrome P450, family 8, subfamily B, polypeptide 1	*ns*	*ns*	-3.1
IGLJ2	immunoglobulin lambda joining 2	*ns*	*ns*	-3.1
RUSC2	RUN and SH3 domain containing 2	-3.1	*ns*	*ns*
GJA5	gap junction protein alpha 5	-3.1	*ns*	*ns*
LOC101928955	uncharacterized LOC101928955	-3.0	*ns*	*ns*
FBXO46	F-box protein 46	-3.0	*ns*	*ns*
LOC105376713	uncharacterized LOC105376713	*ns*	*ns*	-3.0
PANX1	pannexin 1	-3.0	*ns*	*ns*
TEAD1	TEA domain family member 1 (SV40 transcriptional enhancer factor)	-3.0	*ns*	*ns*
ELF4	E74-like factor 4 (ets domain transcription factor)	-2.9	*ns*	*ns*
POTEG	POTE ankyrin domain family, member G	-2.9	*ns*	*ns*
PFKP	phosphofructokinase, platelet	-2.9	*ns*	*ns*
SH3BP5	SH3-domain binding protein 5 (BTK-associated)	-2.9	*ns*	*ns*
SLC6A8	solute carrier family 6 (neurotransmitter transporter), member 8	-2.9	*ns*	*ns*
TCP11L1	t-complex 11, testis-specific-like 1	-2.9	*ns*	*ns*
C18orf54	chromosome 18 open reading frame 54	*ns*	*ns*	-2.9
TCEA3	transcription elongation factor A (SII), 3	-2.9	*ns*	*ns*
SFN	stratifin	-2.9	*ns*	*ns*
SUGCT	succinyl-CoA:glutarate-CoA transferase	-2.9	*ns*	*ns*
LOC101929140	uncharacterized LOC101929140	-2.9	*ns*	*ns*
SLC44A5	solute carrier family 44, member 5	*ns*	*ns*	-2.9
RAB25	RAB25, member RAS oncogene family	*ns*	*ns*	-2.9
TANC2	tetratricopeptide repeat, ankyrin repeat and coiled-coil containing 2	-2.9	*ns*	*ns*
SLC7A4	solute carrier family 7, member 4	*ns*	*ns*	-2.9
BMT2	base methyltransferase of 25S rRNA 2 homolog	-2.8	*ns*	*ns*
IPMK	inositol polyphosphate multikinase	-2.8	*ns*	*ns*
KIF21A	kinesin family member 21A	-2.8	*ns*	*ns*
TSC22D1	TSC22 domain family, member 1	-2.8	*ns*	*ns*
DSP	desmoplakin	*ns*	*ns*	-2.8
CORO2A	coronin, actin binding protein, 2A	-2.8	*ns*	*ns*
BRIP1	BRCA1 interacting protein C-terminal helicase 1	*ns*	*ns*	-2.8
SH3PXD2A	SH3 and PX domains 2A	-2.8	*ns*	*ns*
HYAL4	hyaluronoglucosaminidase 4	*ns*	*ns*	-2.8
LOC105378488	uncharacterized LOC105378488	-2.8	*ns*	*ns*
TAF5L	TAF5-like RNA polymerase II, p300	-2.7	*ns*	*ns*
LOC105376268	uncharacterized LOC105376268	-2.7	*ns*	*ns*
DSCR4	Down syndrome critical region 4	*ns*	*ns*	-2.7
ERO1A	endoplasmic reticulum oxidoreductase alpha	-2.7	*ns*	*ns*
SKIL	SKI-like proto-oncogene	-2.7	*ns*	*ns*

Development • Supplementary information

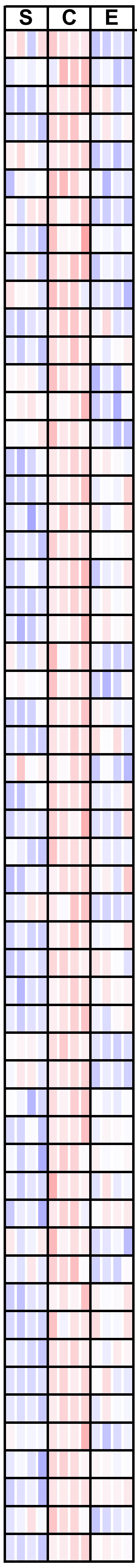

Symbol	Name	Linear Fold Change (*ns*=no significant difference) S vs C	S vs E	E vs C
LOC100505918	uncharacterized LOC100505918	*ns*	*ns*	-2.7
LOC105370960	uncharacterized LOC105370960	*ns*	*ns*	-2.7
COL4A1	collagen, type IV, alpha 1	-2.7	*ns*	*ns*
CDK7	cyclin-dependent kinase 7	-2.7	*ns*	*ns*
LOC101927585	uncharacterized LOC101927585	*ns*	*ns*	-2.7
SUCNR1	succinate receptor 1	*ns*	*ns*	-2.7
LYPD3	LY6	*ns*	*ns*	-2.7
LOC105376938	uncharacterized LOC105376938	-2.7	*ns*	*ns*
LOC285500	uncharacterized LOC285500	*ns*	*ns*	-2.7
BRCA2	breast cancer 2, early onset	*ns*	*ns*	-2.7
SOWAHD	sosondowah ankyrin repeat domain family member D	-2.6	*ns*	*ns*
DFFB	DNA fragmentation factor, 40kDa, beta polypeptide (caspase-activated DNase)	-2.6	*ns*	*ns*
MME	membrane metallo-endopeptidase	*ns*	*ns*	-2.6
LOC102723985	uncharacterized LOC102723985	*ns*	*ns*	-2.6
ZNF90	zinc finger protein 90	*ns*	*ns*	-2.6
MFAP2	microfibrillar associated protein 2	-2.6	*ns*	*ns*
MBNL2	muscleblind-like splicing regulator 2	-2.6	*ns*	*ns*
LOC101927027	uncharacterized LOC101927027	-2.6	*ns*	*ns*
PRKD3	protein kinase D3	-2.6	*ns*	*ns*
SLC1A3	solute carrier family 1 (glial high affinity glutamate transporter), member 3	-2.6	*ns*	*ns*
VAV2	vav 2 guanine nucleotide exchange factor	-2.6	*ns*	*ns*
MOB3A	MOB kinase activator 3A	-2.5	*ns*	*ns*
OVOL1	ovo-like zinc finger 1	*ns*	*ns*	-2.5
ARHGAP24	Rho GTPase activating protein 24	*ns*	*ns*	-2.5
COL4A2	collagen, type IV, alpha 2	-2.5	*ns*	*ns*
GPX3	glutathione peroxidase 3	-2.5	*ns*	*ns*
ZNF662	zinc finger protein 662	*ns*	*ns*	-2.5
PRKAB2	protein kinase, AMP-activated, beta 2 non-catalytic subunit	-2.5	*ns*	*ns*
PROSER2-AS1	PROSER2 antisense RNA 1	-2.5	*ns*	*ns*
ZNF100	zinc finger protein 100	-2.5	*ns*	*ns*
MFAP5	microfibrillar associated protein 5	-2.5	*ns*	*ns*
PLIN2	perilipin 2	*ns*	*ns*	-2.5
SLC10A7	solute carrier family 10, member 7	-2.5	*ns*	*ns*
SOWAHC	sosondowah ankyrin repeat domain family member C	-2.5	*ns*	*ns*
HYI	hydroxypyruvate isomerase (putative)	-2.5	*ns*	*ns*
PLAAT3	phospholipase A and acyltransferase 3	-2.4	*ns*	*ns*
HMGN3-AS1	HMGN3 antisense RNA 1	*ns*	*ns*	-2.4
PLCL2	phospholipase C-like 2	*ns*	*ns*	-2.4
SH3BP5-AS1	SH3BP5 antisense RNA 1	-2.4	*ns*	*ns*
JPT2	Jupiter microtubule associated homolog 2	-2.4	*ns*	*ns*
AP3S1	adaptor-related protein complex 3, sigma 1 subunit	-2.4	*ns*	*ns*
CHRDL2	chordin-like 2	-2.4	*ns*	*ns*
SCAMP1	secretory carrier membrane protein 1	-2.4	*ns*	*ns*
TK1	thymidine kinase 1, soluble	*ns*	*ns*	-2.4
CCSER1	coiled-coil serine rich protein 1	*ns*	*ns*	-2.4
NDST1	N-deacetylase	-2.4	*ns*	*ns*
PTH1R	parathyroid hormone 1 receptor	-2.4	*ns*	*ns*
ACTA1	actin, alpha 1, skeletal muscle	-2.4	*ns*	*ns*
PPP1R1C	protein phosphatase 1, regulatory (inhibitor) subunit 1C	-2.4	*ns*	*ns*
ZBTB2	zinc finger and BTB domain containing 2	-2.4	*ns*	*ns*
TLE1	transducin-like enhancer of split 1 (E(sp1) homolog, Drosophila)	*ns*	*ns*	-2.4
CCNG2	cyclin G2	-2.4	*ns*	*ns*
PARP14	poly(ADP-ribose) polymerase family member 14	-2.4	*ns*	*ns*
SLC27A6	solute carrier family 27 (fatty acid transporter), member 6	*ns*	*ns*	-2.4
B3GNT7	UDP-GlcNAc:betaGal beta-1,3-N-acetylglucosaminyltransferase 7	-2.3	*ns*	*ns*

Development • Supplementary information

Development: doi:10.1242/dev.199626: Supplementary information

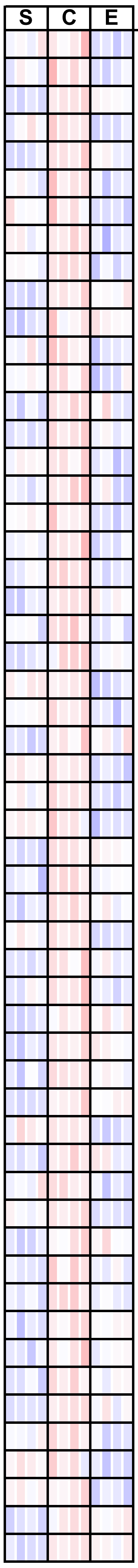

Symbol	Name	S vs C	S vs E	E vs C
		Linear Fold Change (*ns*=no significant difference)		
PHKA2	phosphorylase kinase, alpha 2 (liver)	*ns*	*ns*	-2.3
ZNF93	zinc finger protein 93	*ns*	*ns*	-2.3
CDKN1C	cyclin-dependent kinase inhibitor 1C (p57, Kip2)	-2.3	*ns*	*ns*
IGF2BP2	insulin-like growth factor 2 mRNA binding protein 2	*ns*	*ns*	-2.3
GPR146	G protein-coupled receptor 146	-2.3	*ns*	*ns*
PLEKHA5	pleckstrin homology domain containing, family A member 5	*ns*	*ns*	-2.3
LMNB1	lamin B1	*ns*	*ns*	-2.3
BIRC5	baculoviral IAP repeat containing 5	*ns*	*ns*	-2.3
BZW2	basic leucine zipper and W2 domains 2	*ns*	*ns*	-2.3
MARVELD1	MARVEL domain containing 1	-2.3	*ns*	*ns*
LRP10	LDL receptor related protein 10	-2.3	*ns*	*ns*
LOC102723885	uncharacterized LOC102723885	*ns*	*ns*	-2.3
NR6A1	nuclear receptor subfamily 6, group A, member 1	*ns*	*ns*	-2.3
SLC16A3	solute carrier family 16 (monocarboxylate transporter), member 3	-2.3	*ns*	*ns*
FOXJ3	forkhead box J3	-2.3	*ns*	*ns*
RASL11B	RAS-like, family 11, member B	*ns*	*ns*	-2.3
FANCE	Fanconi anemia complementation group E	*ns*	*ns*	-2.3
LOC105373786	uncharacterized LOC105373786	*ns*	*ns*	-2.3
ESRP1	epithelial splicing regulatory protein 1	*ns*	*ns*	-2.3
RAB30	RAB30, member RAS oncogene family	*ns*	*ns*	-2.2
SLC39A11	solute carrier family 39, member 11	-2.2	*ns*	*ns*
ARL5B	ADP-ribosylation factor like GTPase 5B	*ns*	*ns*	-2.2
TAGLN2	transgelin 2	-2.2	*ns*	*ns*
CYB5R1	cytochrome b5 reductase 1	*ns*	*ns*	-2.2
DAG1	dystroglycan 1 (dystrophin-associated glycoprotein 1)	*ns*	*ns*	-2.2
MORF4L2	mortality factor 4 like 2	-2.2	*ns*	*ns*
MIR205HG	MIR205 host gene	*ns*	*ns*	-2.2
CENPJ	centromere protein J	*ns*	*ns*	-2.2
SORT1	sortilin 1	*ns*	*ns*	-2.2
CPVL	carboxypeptidase, vitellogenic-like	-2.2	*ns*	*ns*
NDFIP2	Nedd4 family interacting protein 2	-2.2	*ns*	*ns*
MYCN	v-myc avian myelocytomatosis viral oncogene neuroblastoma derived homolog	-2.2	*ns*	*ns*
ITPRID2	ITPR interacting domain containing 2	*ns*	*ns*	-2.2
FAM78A	family with sequence similarity 78, member A	-2.2	*ns*	*ns*
KRT18P54	keratin 18 pseudogene 54	*ns*	*ns*	-2.2
NEK6	NIMA-related kinase 6	-2.2	*ns*	*ns*
LHX2	LIM homeobox 2	-2.2	*ns*	*ns*
CECR9	cat eye syndrome chromosome region, candidate 9 (non-protein coding)	-2.2	*ns*	*ns*
MAPK3	mitogen-activated protein kinase 3	-2.1	*ns*	*ns*
RPSAP58	ribosomal protein SA pseudogene 58	*ns*	*ns*	-2.1
NFIL3	nuclear factor, interleukin 3 regulated	-2.1	*ns*	*ns*
KCNQ1OT1	KCNQ1 opposite strand	*ns*	*ns*	-2.1
FAM120AOS	family with sequence similarity 120A opposite strand	*ns*	*ns*	-2.1
LOC100131262	uncharacterized LOC100131262	-2.1	*ns*	*ns*
LOC101929460	uncharacterized LOC101929460	-2.1	*ns*	*ns*
NKRF	NFKB repressing factor	-2.1	*ns*	*ns*
GPR150	G protein-coupled receptor 150	-2.1	*ns*	*ns*
DLK2	delta-like 2 homolog (Drosophila)	-2.1	*ns*	*ns*
HACD2	3-hydroxyacyl-CoA dehydratase 2	-2.1	*ns*	*ns*
ARHGAP10	Rho GTPase activating protein 10	-2.1	*ns*	*ns*
FAM83D	family with sequence similarity 83, member D	*ns*	*ns*	-2.1
KNTC1	kinetochore associated 1	*ns*	*ns*	-2.1
LOC105377606	uncharacterized LOC105377606	*ns*	*ns*	-2.1
CLDND1	claudin domain containing 1	-2.1	*ns*	*ns*
EDEM2	ER degradation enhancer, mannosidase alpha-like 2	-2.1	*ns*	*ns*

Development • Supplementary information

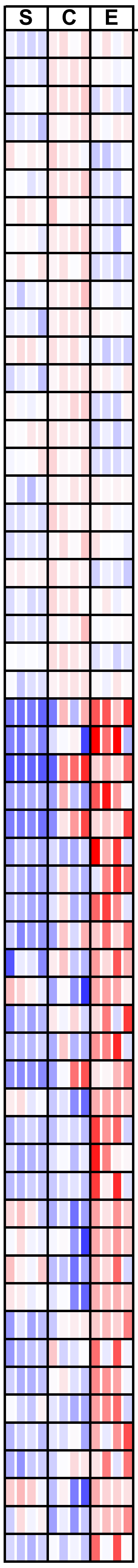

Symbol	Name	Linear Fold Change (*ns*=no significant difference) S vs C	S vs E	E vs C
LOC440461	Rho GTPase activating protein 27 pseudogene	-2.1	*ns*	*ns*
PPP3CA	protein phosphatase 3, catalytic subunit, alpha isozyme	-2.1	*ns*	*ns*
SLC29A3	solute carrier family 29 (equilibrative nucleoside transporter), member 3	*ns*	*ns*	-2.1
EIF2AK2	eukaryotic translation initiation factor 2-alpha kinase 2	-2.1	*ns*	*ns*
ANXA3	annexin A3	*ns*	*ns*	-2.1
CSPP1	centrosome and spindle pole associated protein 1	*ns*	*ns*	-2.1
TRIM24	tripartite motif containing 24	*ns*	*ns*	-2.1
USP6NL	USP6 N-terminal like	*ns*	*ns*	-2.1
ACVR2B	activin A receptor type IIB	*ns*	*ns*	-2.1
ERBB3	erb-b2 receptor tyrosine kinase 3	-2.0	*ns*	*ns*
PTPN4	protein tyrosine phosphatase, non-receptor type 4 (megakaryocyte)	-2.0	*ns*	*ns*
POLI	polymerase (DNA directed) iota	*ns*	*ns*	-2.0
RSPRY1	ring finger and SPRY domain containing 1	-2.0	*ns*	*ns*
PRRG1	proline rich Gla (G-carboxyglutamic acid) 1	*ns*	*ns*	-2.0
SLC49A4	solute carrier family 49 member 4	*ns*	*ns*	-2.0
NEDD9	neural precursor cell expressed, developmentally down-regulated 9	*ns*	*ns*	-2.0
SNORA84	small nucleolar RNA, H	-2.0	*ns*	*ns*
LOXL1	lysyl oxidase-like 1	-2.0	*ns*	*ns*
GAA	glucosidase, alpha; acid	-2.0	*ns*	*ns*
ORC1	origin recognition complex subunit 1	*ns*	*ns*	-2.0
CDK17	cyclin-dependent kinase 17	-2.0	*ns*	*ns*
HK2	hexokinase 2	-2.0	*ns*	*ns*
PXDC1	PX domain containing 1	*ns*	*ns*	-2.0
KRTCAP3	keratinocyte associated protein 3	-2.0	*ns*	*ns*
CXCL9	chemokine (C-X-C motif) ligand 9	*ns*	-50.7	*ns*
PAEP	progestagen-associated endometrial protein	*ns*	-42.6	*ns*
OR5H14	olfactory receptor, family 5, subfamily H, member 14	*ns*	-25.9	*ns*
LOC100506530	uncharacterized LOC100506530	*ns*	-16.6	*ns*
TAC3	tachykinin 3	*ns*	-16.1	*ns*
LOC105374458	uncharacterized LOC105374458	*ns*	-15.4	*ns*
MYO16-AS1	MYO16 antisense RNA 1	*ns*	-12.6	*ns*
KLRF1	killer cell lectin-like receptor subfamily F, member 1	*ns*	-12.1	*ns*
SLAMF6	SLAM family member 6	*ns*	-11.3	*ns*
LOC105373334	uncharacterized LOC105373334	*ns*	-10.8	*ns*
C1orf54	chromosome 1 open reading frame 54	*ns*	*ns*	9.9
MMP3	matrix metallopeptidase 3	*ns*	-9.6	*ns*
SPINK2	serine peptidase inhibitor, Kazal type 2 (acrosin-trypsin inhibitor)	*ns*	-9.3	*ns*
LOC105379051	uncharacterized LOC105379051	*ns*	-9.2	*ns*
SRGN	serglycin	*ns*	*ns*	9.2
KLRF2	killer cell lectin-like receptor subfamily F, member 2	*ns*	-8.7	*ns*
KLRC2	killer cell lectin-like receptor subfamily C, member 2	*ns*	-8.6	*ns*
C4BPA	complement component 4 binding protein, alpha	*ns*	-8.6	*ns*
CAMK2D	calcium	*ns*	*ns*	8.0
CYBB	cytochrome b-245, beta polypeptide	*ns*	*ns*	7.3
CES1P1	carboxylesterase 1 pseudogene 1	*ns*	*ns*	7.0
VCAM1	vascular cell adhesion molecule 1	*ns*	*ns*	6.9
ANTXR1	anthrax toxin receptor 1	*ns*	-6.8	*ns*
AADAC	arylacetamide deacetylase	*ns*	-6.7	*ns*
GNLY	granulysin	*ns*	-6.7	*ns*
IGFBP5	insulin like growth factor binding protein 5	*ns*	*ns*	6.6
CCL8	chemokine (C-C motif) ligand 8	*ns*	-6.4	*ns*
TM4SF5	transmembrane 4 L six family member 5	*ns*	-6.3	*ns*
MS4A4A	membrane-spanning 4-domains, subfamily A, member 4A	*ns*	*ns*	6.3
GBP4	guanylate binding protein 4	*ns*	*ns*	6.3
MUC1	mucin 1, cell surface associated	*ns*	-6.2	*ns*

Development • Supplementary information

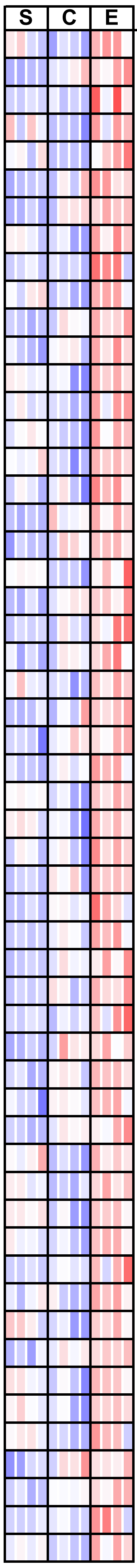

Symbol	Name	Linear Fold Change (*ns*=no significant difference) S vs C	S vs E	E vs C
MMRN1	multimerin 1	*ns*	*ns*	*6.2*
OAS2	2-5-oligoadenylate synthetase 2	*ns*	*-6.2*	*ns*
NAPSB	napsin B aspartic peptidase, pseudogene	*ns*	*-5.6*	*ns*
ENPP4	ectonucleotide pyrophosphatase	*ns*	*ns*	*6.0*
TOX	thymocyte selection-associated high mobility group box	*ns*	*ns*	*5.9*
LINC01338	long intergenic non-protein coding RNA 1338	*ns*	*-5.9*	*ns*
NTN4	netrin 4	*ns*	*-5.8*	*ns*
C3AR1	complement component 3a receptor 1	*ns*	*ns*	*5.7*
CLTRN	collectrin, amino acid transport regulator	*ns*	*-5.3*	*ns*
EDNRA	endothelin receptor type A	*ns*	*ns*	*5.6*
ST3GAL5	ST3 beta-galactoside alpha-2,3-sialyltransferase 5	*ns*	*-5.4*	*ns*
S100A8	S100 calcium binding protein A8	*ns*	*-5.3*	*ns*
CD163	CD163 molecule	*ns*	*ns*	*5.3*
BICC1	BicC family RNA binding protein 1	*ns*	*ns*	*5.3*
LOC102724587	uncharacterized LOC102724587	*ns*	*ns*	*5.3*
VIM-AS1	VIM antisense RNA 1	*ns*	*ns*	*5.2*
GPR65	G protein-coupled receptor 65	*ns*	*-4.5*	*ns*
CLU	clusterin	*ns*	*-5.2*	*ns*
TDRD6	tudor domain containing 6	*ns*	*-5.1*	*ns*
ADGRF5	adhesion G protein-coupled receptor F5	*ns*	*ns*	*5.0*
EPHB2	EPH receptor B2	*ns*	*-5.0*	*ns*
LOC105374732	uncharacterized LOC105374732	*ns*	*-4.9*	*ns*
CRIP1	cysteine-rich protein 1 (intestinal)	*ns*	*-4.9*	*ns*
LOC100996713	uncharacterized LOC100996713	*ns*	*ns*	*4.8*
SEMA5A	semaphorin 5A	*ns*	*-4.7*	*ns*
IFIT3	interferon-induced protein with tetratricopeptide repeats 3	*ns*	*-4.7*	*ns*
ITGAD	integrin alpha D	*ns*	*-4.7*	*ns*
VSIG4	V-set and immunoglobulin domain containing 4	*ns*	*ns*	*4.7*
PROS1	protein S (alpha)	*ns*	*ns*	*4.6*
F3	coagulation factor III (thromboplastin, tissue factor)	*ns*	*ns*	*4.6*
PLXDC2	plexin domain containing 2	*ns*	*-4.5*	*ns*
LEFTY2	left-right determination factor 2	*ns*	*-4.5*	*ns*
ECM2	extracellular matrix protein 2, female organ and adipocyte specific	*ns*	*-4.5*	*ns*
MYL10	myosin light chain 10	*ns*	*-4.5*	*ns*
LOC101929531	uncharacterized LOC101929531	*ns*	*-4.5*	*ns*
GSTA1	glutathione S-transferase alpha 1	*ns*	*-4.4*	*ns*
DAW1	dynein assembly factor with WDR repeat domains 1	*ns*	*-4.4*	*ns*
TTC39C	tetratricopeptide repeat domain 39C	*ns*	*-4.4*	*ns*
DUSP23	dual specificity phosphatase 23	*ns*	*-4.4*	*ns*
SMCO3	single-pass membrane protein with coiled-coil domains 3	*ns*	*-4.4*	*ns*
CD44	CD44 molecule (Indian blood group)	*ns*	*ns*	*4.3*
SLC12A2	solute carrier family 12 (sodium	*ns*	*ns*	*4.3*
MAGEH1	MAGE family member H1	*ns*	*ns*	*4.3*
RNF125	ring finger protein 125, E3 ubiquitin protein ligase	*ns*	*ns*	*4.2*
PZP	pregnancy-zone protein	*ns*	*-4.2*	*ns*
EHD2	EH domain containing 2	*ns*	*ns*	*4.2*
CD109	CD109 molecule	*ns*	*ns*	*4.2*
CCN2	cellular communication network factor 2	*ns*	*-4.1*	*ns*
ITGA4	integrin alpha 4	*ns*	*ns*	*4.1*
SLC9A9	solute carrier family 9, subfamily A (NHE9, cation proton antiporter 9), member 9	*ns*	*ns*	*4.1*
KLRD1	killer cell lectin-like receptor subfamily D, member 1	*ns*	*ns*	*4.0*
SERPINE1	serpin peptidase inhibitor, clade E (nexin, plasminogen activator inhibitor type 1), member 1	*ns*	*-4.0*	*ns*
CRLF1	cytokine receptor-like factor 1	*ns*	*-4.0*	*ns*
PGM5-AS1	PGM5 antisense RNA 1	*ns*	*-4.0*	*ns*
PRKD1	protein kinase D1	*ns*	*ns*	*4.0*

Development • Supplementary information

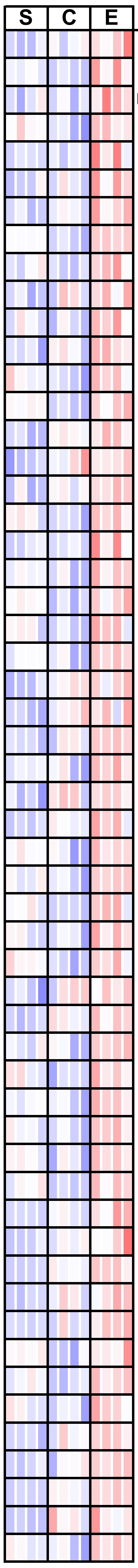

Symbol	Name	Linear Fold Change (*ns*=no significant difference) S vs C	S vs E	E vs C
IGLC2	immunoglobulin lambda constant 2 (Kern-Oz- marker)	*ns*	-3.9	*ns*
TOB1	transducer of ERBB2, 1	*ns*	*ns*	3.9
LL22NC03-104C7.1	uncharacterized LOC105373004	*ns*	-3.7	*ns*
ACSS3	acyl-CoA synthetase short-chain family member 3	*ns*	*ns*	3.9
ADGRF1	adhesion G protein-coupled receptor F1	*ns*	-3.8	*ns*
KCNQ5-IT1	KCNQ5 intronic transcript 1	*ns*	-3.8	*ns*
CD69	CD69 molecule	*ns*	-3.8	*ns*
GIMAP7	GTPase, IMAP family member 7	*ns*	*ns*	3.8
MYH11	myosin, heavy chain 11, smooth muscle	*ns*	*ns*	3.7
LOC102724156	uncharacterized LOC102724156	*ns*	-3.7	*ns*
SNORA47	small nucleolar RNA, H	*ns*	*ns*	3.7
PMCH	pro-melanin-concentrating hormone	*ns*	-3.7	*ns*
MAP9	microtubule-associated protein 9	*ns*	*ns*	3.7
GIMAP4	GTPase, IMAP family member 4	*ns*	*ns*	3.7
LOC105377590	uncharacterized LOC105377590	*ns*	-3.7	*ns*
OAS3	2-5-oligoadenylate synthetase 3	*ns*	-3.7	*ns*
PSENEN	presenilin enhancer gamma secretase subunit	*ns*	-3.6	*ns*
PCGF5	polycomb group ring finger 5	*ns*	*ns*	3.6
CD82	CD82 molecule	*ns*	-3.6	*ns*
PLEK	pleckstrin	*ns*	*ns*	3.6
PRLR	prolactin receptor	*ns*	*ns*	3.6
PLA2G4A	phospholipase A2, group IVA (cytosolic, calcium-dependent)	*ns*	*ns*	3.6
GASK1B	golgi associated kinase 1B	*ns*	*ns*	3.6
XAF1	XIAP associated factor 1	*ns*	-3.6	*ns*
GPC4	glypican 4	*ns*	-3.5	*ns*
MORN2	MORN repeat containing 2	*ns*	-3.5	*ns*
MPEG1	macrophage expressed 1	*ns*	*ns*	3.5
ZNF675	zinc finger protein 675	*ns*	-3.5	*ns*
PODNL1	podocan-like 1	*ns*	-3.5	*ns*
CDH11	cadherin 11, type 2, OB-cadherin (osteoblast)	*ns*	*ns*	3.5
GDAP1	ganglioside induced differentiation associated protein 1	*ns*	*ns*	3.5
HSD17B6	hydroxysteroid (17-beta) dehydrogenase 6	*ns*	*ns*	3.5
DYNC2LI1	dynein, cytoplasmic 2, light intermediate chain 1	*ns*	*ns*	3.5
ITM2A	integral membrane protein 2A	*ns*	*ns*	3.5
SLC30A4	solute carrier family 30 (zinc transporter), member 4	*ns*	-3.5	*ns*
KCNA4	potassium channel, voltage gated shaker related subfamily A, member 4	*ns*	-3.5	*ns*
ALOX5AP	arachidonate 5-lipoxygenase-activating protein	*ns*	*ns*	3.4
EDIL3	EGF-like repeats and discoidin I-like domains 3	*ns*	*ns*	3.4
BDH2	3-hydroxybutyrate dehydrogenase, type 2	*ns*	*ns*	3.4
CD86	CD86 molecule	*ns*	*ns*	3.4
RETREG1	reticulophagy regulator 1	*ns*	*ns*	3.4
PDE8B	phosphodiesterase 8B	*ns*	*ns*	3.4
LOC101928919	uncharacterized LOC101928919	*ns*	-3.4	*ns*
LRRC15	leucine rich repeat containing 15	*ns*	-3.4	*ns*
KHDRBS3	KH domain containing, RNA binding, signal transduction associated 3	*ns*	-3.4	*ns*
TSPAN8	tetraspanin 8	*ns*	-3.4	*ns*
ROS1	ROS proto-oncogene 1 , receptor tyrosine kinase	*ns*	-3.3	*ns*
TEK	TEK tyrosine kinase, endothelial	*ns*	*ns*	3.3
FKBP5	FK506 binding protein 5	*ns*	*ns*	3.3
PRTFDC1	phosphoribosyl transferase domain containing 1	*ns*	*ns*	3.3
HACD1	3-hydroxyacyl-CoA dehydratase 1	*ns*	-3.3	*ns*
GADD45B	growth arrest and DNA-damage-inducible, beta	*ns*	-3.3	*ns*
CHST7	carbohydrate (N-acetylglucosamine 6-O) sulfotransferase 7	*ns*	-3.3	*ns*
MAMDC2-AS1	MAMDC2 antisense RNA 1	*ns*	-3.3	*ns*
MAF	v-maf avian musculoaponeurotic fibrosarcoma oncogene homolog	*ns*	*ns*	3.3

Development • Supplementary information

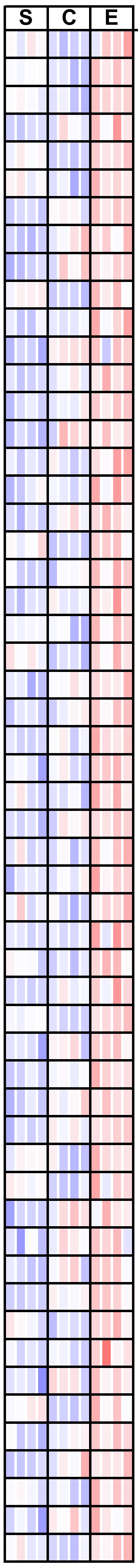

Symbol	Name	S vs C	S vs E	E vs C
		Linear Fold Change (*ns*=no significant difference)		
RHOJ	ras homolog family member J	*ns*	*ns*	3.3
VIM	vimentin	*ns*	*ns*	3.3
ZFAND4	zinc finger, AN1-type domain 4	*ns*	*ns*	3.3
WDR86-AS1	WDR86 antisense RNA 1	*ns*	-3.3	*ns*
ITPR1	inositol 1,4,5-trisphosphate receptor, type 1	*ns*	*ns*	3.3
ARRDC4	arrestin domain containing 4	*ns*	*ns*	3.3
ATP13A3	ATPase type 13A3	*ns*	-3.3	*ns*
MCAM	melanoma cell adhesion molecule	*ns*	-3.3	*ns*
SLC43A3	solute carrier family 43, member 3	*ns*	-3.3	*ns*
MIPOL1	mirror-image polydactyly 1	*ns*	*ns*	3.3
CCL5	chemokine (C-C motif) ligand 5	*ns*	-3.3	*ns*
APOC1	apolipoprotein C-I	*ns*	-3.2	*ns*
CYP39A1	cytochrome P450, family 39, subfamily A, polypeptide 1	*ns*	-3.2	*ns*
TTPAL	tocopherol (alpha) transfer protein-like	*ns*	-3.2	*ns*
STAB2	stabilin 2	*ns*	-3.2	*ns*
HSD17B14	hydroxysteroid (17-beta) dehydrogenase 14	*ns*	-3.2	*ns*
ZFP36	ZFP36 ring finger protein	*ns*	-3.2	*ns*
MGAT2	mannosyl (alpha-1,6-)-glycoprotein beta-1,2-N-acetylglucosaminyltransferase	*ns*	-3.2	*ns*
CDH13	cadherin 13	*ns*	*ns*	3.2
MIR3918	microRNA 3918	*ns*	-3.2	*ns*
PPDPF	pancreatic progenitor cell differentiation and proliferation factor	*ns*	-3.2	*ns*
LRP12	LDL receptor related protein 12	*ns*	*ns*	3.2
CADPS2	Ca++-dependent secretion activator 2	*ns*	*ns*	3.2
LOC105377134	uncharacterized LOC105377134	*ns*	-3.2	*ns*
HERC6	HECT and RLD domain containing E3 ubiquitin protein ligase family member 6	*ns*	-3.2	*ns*
SLAMF8	SLAM family member 8	*ns*	-3.1	*ns*
RAB32	RAB32, member RAS oncogene family	*ns*	-3.1	*ns*
TREM2	triggering receptor expressed on myeloid cells 2	*ns*	*ns*	3.1
SMIM14	small integral membrane protein 14	*ns*	-3.1	*ns*
LINC01139	long intergenic non-protein coding RNA 1139	*ns*	*ns*	3.1
ABCC3	ATP binding cassette subfamily C member 3	*ns*	-3.1	*ns*
BTN3A2	butyrophilin, subfamily 3, member A2	*ns*	*ns*	3.1
ITGAM	integrin, alpha M (complement component 3 receptor 3 subunit)	*ns*	-3.1	*ns*
TNFSF8	tumor necrosis factor (ligand) superfamily, member 8	*ns*	*ns*	3.1
LINC00706	long intergenic non-protein coding RNA 706	*ns*	-3.1	*ns*
SRPX2	sushi-repeat containing protein, X-linked 2	*ns*	*ns*	3.1
PIGK	phosphatidylinositol glycan anchor biosynthesis class K	*ns*	-3.1	*ns*
IL15RA	interleukin 15 receptor, alpha	*ns*	-3.1	*ns*
PVR	poliovirus receptor	*ns*	-3.1	*ns*
SFT2D2	SFT2 domain containing 2	*ns*	-3.1	*ns*
FCGR1A	Fc fragment of IgG, high affinity Ia, receptor (CD64)	*ns*	*ns*	3.1
NID2	nidogen 2 (osteonidogen)	*ns*	*ns*	3.1
TMEM31	transmembrane protein 31	*ns*	-3.1	*ns*
LINC00484	long intergenic non-protein coding RNA 484	*ns*	-3.1	*ns*
PRXL2C	peroxiredoxin like 2C	*ns*	-3.1	*ns*
FLNA	filamin A, alpha	*ns*	-3.1	*ns*
C8orf48	chromosome 8 open reading frame 48	*ns*	*ns*	3.1
SERTM2	serine rich and transmembrane domain containing 2	*ns*	-3.1	*ns*
ANKRD36BP1	ankyrin repeat domain 36B pseudogene 1	*ns*	-3.1	*ns*
PLCB4	phospholipase C, beta 4	*ns*	*ns*	3.1
RPGRIP1L	RPGRIP1-like	*ns*	-3.1	*ns*
CBL	Cbl proto-oncogene, E3 ubiquitin protein ligase	*ns*	-3.0	*ns*
ATP8B4	ATPase, class I, type 8B, member 4	*ns*	*ns*	3.0
SAA1	serum amyloid A1	*ns*	-3.0	*ns*
PLCE1	phospholipase C, epsilon 1	*ns*	*ns*	3.0

Development • Supplementary information

Development: doi:10.1242/dev.199626: Supplementary information

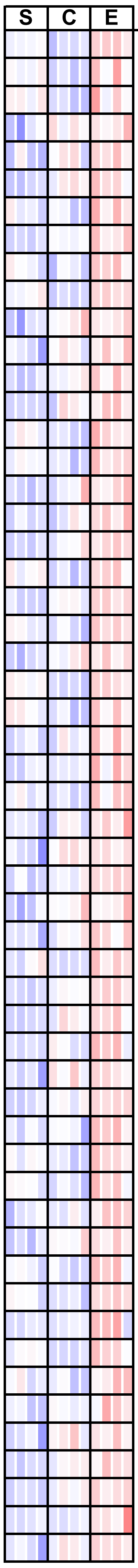

Symbol	Name	Linear Fold Change (*ns*=no significant difference) S vs C	S vs E	E vs C
DEPP1	DEPP1 autophagy regulator	*ns*	*ns*	3.0
PKP4	plakophilin 4	*ns*	*ns*	3.0
ADGRD1	adhesion G protein-coupled receptor D1	*ns*	*ns*	3.0
GBP1	guanylate binding protein 1, interferon-inducible	*ns*	-3.0	*ns*
SNX16	sorting nexin 16	*ns*	-3.0	*ns*
OSR2	odd-skipped related transciption factor 2	*ns*	-3.0	*ns*
CHSY3	chondroitin sulfate synthase 3	*ns*	*ns*	3.0
IKZF3	IKAROS family zinc finger 3	*ns*	-3.0	*ns*
MED14OS	MED14 opposite strand	*ns*	*ns*	3.0
FGD5	FYVE, RhoGEF and PH domain containing 5	*ns*	*ns*	3.0
ZDHHC9	zinc finger, DHHC-type containing 9	*ns*	-3.0	*ns*
LINC01357	long intergenic non-protein coding RNA 1357	*ns*	-3.0	*ns*
DUSP1	dual specificity phosphatase 1	*ns*	-2.9	*ns*
LOC101927780	uncharacterized LOC101927780	*ns*	-2.9	*ns*
CACNA2D1	calcium channel, voltage-dependent, alpha 2	*ns*	*ns*	2.9
PAPSS2	3-phosphoadenosine 5-phosphosulfate synthase 2	*ns*	*ns*	2.9
BST2	bone marrow stromal cell antigen 2	*ns*	-2.9	*ns*
IRF8	interferon regulatory factor 8	*ns*	-2.9	*ns*
HLA-L	major histocompatibility complex, class I, L (pseudogene)	*ns*	-2.9	*ns*
CMTM7	CKLF-like MARVEL transmembrane domain containing 7	*ns*	*ns*	2.9
GNG4	guanine nucleotide binding protein (G protein), gamma 4	*ns*	-2.9	*ns*
AIF1	allograft inflammatory factor 1	*ns*	*ns*	2.9
DOP1B	DOP1 leucine zipper like protein B	*ns*	-2.9	*ns*
PYCARD	PYD and CARD domain containing	*ns*	*ns*	2.9
SPRED1	sprouty-related, EVH1 domain containing 1	*ns*	*ns*	2.9
KIR2DL2	killer cell immunoglobulin-like receptor, two domains, long cytoplasmic tail, 2	*ns*	-2.9	*ns*
PCDHB2	protocadherin beta 2	*ns*	-2.9	*ns*
NCKAP1L	NCK-associated protein 1-like	*ns*	*ns*	2.9
CABYR	calcium binding tyrosine-(Y)-phosphorylation regulated	*ns*	-2.9	*ns*
HLTF	helicase-like transcription factor	*ns*	-2.9	*ns*
LPCAT2	lysophosphatidylcholine acyltransferase 2	*ns*	-2.8	*ns*
TRIM21	tripartite motif containing 21	*ns*	-2.8	*ns*
RNF24	ring finger protein 24	*ns*	-2.8	*ns*
SLFN13	schlafen family member 13	*ns*	*ns*	2.8
SH3BGRL3	SH3 domain binding glutamate-rich protein like 3	*ns*	-2.8	*ns*
SELENOM	selenoprotein M	*ns*	-2.8	*ns*
CYBA	cytochrome b-245, alpha polypeptide	*ns*	-2.8	*ns*
ATP11C	ATPase, class VI, type 11C	*ns*	-2.8	*ns*
IFITM4P	interferon induced transmembrane protein 4 pseudogene	*ns*	-2.8	*ns*
CMC1	C-x(9)-C motif containing 1	*ns*	-2.8	*ns*
TUBA1A	tubulin, alpha 1a	*ns*	*ns*	2.8
LOC100506688	uncharacterized LOC100506688	*ns*	*ns*	2.8
LILRB4	leukocyte immunoglobulin-like receptor, subfamily B (with TM and ITIM domains), member 4	*ns*	-2.7	*ns*
FOSL2	FOS-like antigen 2	*ns*	-2.7	*ns*
TNFSF13	tumor necrosis factor (ligand) superfamily, member 13	*ns*	*ns*	2.7
ZNF32-AS1	ZNF32 antisense RNA 1	*ns*	*ns*	2.7
LOC101928047	uncharacterized LOC101928047	*ns*	-2.7	*ns*
ADAMTS9-AS2	ADAMTS9 antisense RNA 2	*ns*	*ns*	2.7
TENT5C	terminal nucleotidyltransferase 5C	*ns*	*ns*	2.7
STMN2	stathmin 2	*ns*	-2.7	*ns*
IFIT5	interferon-induced protein with tetratricopeptide repeats 5	*ns*	-2.7	*ns*
HABP4	hyaluronan binding protein 4	*ns*	-2.7	*ns*
PIK3R1	phosphoinositide-3-kinase, regulatory subunit 1 (alpha)	*ns*	-2.7	*ns*
GGT2	gamma-glutamyltransferase 2	*ns*	-2.7	*ns*
CCDC80	coiled-coil domain containing 80	*ns*	-2.7	*ns*

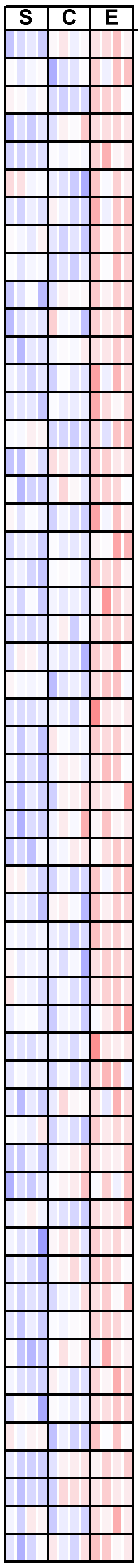

Symbol	Name	Linear Fold Change (*ns*=no significant difference) S vs C	S vs E	E vs C
C12orf60	chromosome 12 open reading frame 60	*ns*	-2.7	*ns*
CLUAP1	clusterin associated protein 1	*ns*	*ns*	2.7
FNDC1	fibronectin type III domain containing 1	*ns*	*ns*	2.7
VAT1	vesicle amine transport 1	*ns*	-2.7	*ns*
TACR3	tachykinin receptor 3	*ns*	-2.7	*ns*
PIAS2	protein inhibitor of activated STAT 2	*ns*	*ns*	2.7
GRIA3	glutamate receptor, ionotropic, AMPA 3	*ns*	-2.7	*ns*
BICD1	bicaudal D homolog 1 (Drosophila)	*ns*	*ns*	2.7
CLIP3	CAP-GLY domain containing linker protein 3	*ns*	*ns*	2.6
SNX10	sorting nexin 10	*ns*	-2.6	*ns*
BST1	bone marrow stromal cell antigen 1	*ns*	-2.6	*ns*
C19orf53	chromosome 19 open reading frame 53	*ns*	-2.6	*ns*
MITF	microphthalmia-associated transcription factor	*ns*	-2.6	*ns*
CHST15	carbohydrate (N-acetylgalactosamine 4-sulfate 6-O) sulfotransferase 15	*ns*	-2.6	*ns*
KIF13B	kinesin family member 13B	*ns*	*ns*	2.6
LOX	lysyl oxidase	*ns*	-2.6	*ns*
MYDGF	myeloid-derived growth factor	*ns*	-2.6	*ns*
LACTB2	lactamase, beta 2	*ns*	*ns*	2.6
RSAD2	radical S-adenosyl methionine domain containing 2	*ns*	-2.6	*ns*
ERBIN	erbb2 interacting protein	*ns*	-2.6	*ns*
FBXO6	F-box protein 6	*ns*	-2.6	*ns*
TMEM176A	transmembrane protein 176A	*ns*	-2.6	*ns*
NAIP	NLR family, apoptosis inhibitory protein	*ns*	*ns*	2.6
CEP112	centrosomal protein 112kDa	*ns*	*ns*	2.6
C4A	complement component 4A (Rodgers blood group)	*ns*	-2.6	*ns*
RCN3	reticulocalbin 3, EF-hand calcium binding domain	*ns*	-2.6	*ns*
REX1BD	required for excision 1-B domain containing	*ns*	-2.6	*ns*
RECK	reversion-inducing-cysteine-rich protein with kazal motifs	*ns*	-2.6	*ns*
PTK2B	protein tyrosine kinase 2 beta	*ns*	-2.6	*ns*
AAMDC	adipogenesis associated, Mth938 domain containing	*ns*	-2.6	*ns*
ACOT9	acyl-CoA thioesterase 9	*ns*	*ns*	2.6
TMX3	thioredoxin-related transmembrane protein 3	*ns*	-2.6	*ns*
CD4	CD4 molecule	*ns*	*ns*	2.6
ADAP2	ArfGAP with dual PH domains 2	*ns*	*ns*	2.6
TMSB4XP4	thymosin beta 4, X-linked pseudogene 4	*ns*	*ns*	2.6
ATG4A	autophagy related 4A, cysteine peptidase	*ns*	*ns*	2.6
C4B	complement component 4B (Chido blood group)	*ns*	-2.6	*ns*
LOC100506388	uncharacterized LOC100506388	*ns*	*ns*	2.6
DIRAS2	DIRAS family, GTP-binding RAS-like 2	*ns*	-2.6	*ns*
SFMBT2	Scm-like with four mbt domains 2	*ns*	*ns*	2.6
ATRN	attractin	*ns*	-2.6	*ns*
ARHGAP31	Rho GTPase activating protein 31	*ns*	-2.5	*ns*
IQCK	IQ motif containing K	*ns*	*ns*	2.5
DRAM1	DNA-damage regulated autophagy modulator 1	*ns*	-2.5	*ns*
FKBP7	FK506 binding protein 7	*ns*	-2.5	*ns*
CHI3L1	chitinase 3-like 1 (cartilage glycoprotein-39)	*ns*	-2.5	*ns*
SPSB1	splA	*ns*	-2.5	*ns*
CDC42EP5	CDC42 effector protein (Rho GTPase binding) 5	*ns*	-2.5	*ns*
APOBEC3G	apolipoprotein B mRNA editing enzyme, catalytic polypeptide-like 3G	*ns*	-2.5	*ns*
TUBE1	tubulin, epsilon 1	*ns*	-2.5	*ns*
TRAF5	TNF receptor-associated factor 5	*ns*	*ns*	2.5
TCEAL1	transcription elongation factor A (SII)-like 1	*ns*	-2.5	*ns*
ENDOG	endonuclease G	*ns*	-2.5	*ns*
CRYAB	crystallin alpha B	*ns*	*ns*	2.5
PLBD2	phospholipase B domain containing 2	*ns*	-2.5	*ns*

Development • Supplementary information

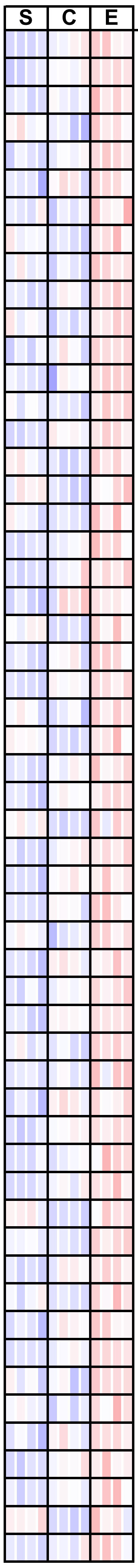

Symbol	Name	Linear Fold Change (*ns*=no significant difference) S vs C	S vs E	E vs C
PLXNB2	plexin B2	*ns*	-2.5	*ns*
CAPNS1	calpain, small subunit 1	*ns*	-2.5	*ns*
CD300A	CD300a molecule	*ns*	-2.5	*ns*
HECTD2	HECT domain containing E3 ubiquitin protein ligase 2	*ns*	*ns*	2.5
CCDC107	coiled-coil domain containing 107	*ns*	-2.5	*ns*
HIF1A-AS2	HIF1A antisense RNA 2	*ns*	-2.5	*ns*
NPR2	natriuretic peptide receptor 2	*ns*	*ns*	2.5
RNF152	ring finger protein 152	*ns*	*ns*	2.5
CD84	CD84 molecule	*ns*	*ns*	2.5
TST	thiosulfate sulfurtransferase (rhodanese)	*ns*	-2.5	*ns*
EID1	EP300 interacting inhibitor of differentiation 1	*ns*	*ns*	2.5
TMEM176B	transmembrane protein 176B	*ns*	-2.5	*ns*
RGS10	regulator of G-protein signaling 10	*ns*	-2.5	*ns*
SOCS5	suppressor of cytokine signaling 5	*ns*	*ns*	2.5
SLC35B2	solute carrier family 35 (adenosine 3-phospho 5-phosphosulfate transporter), member B2	*ns*	-2.5	*ns*
LOC102724776	uncharacterized LOC102724776	*ns*	*ns*	2.5
CALHM5	calcium homeostasis modulator family member 5	*ns*	*ns*	2.5
TFEC	transcription factor EC	*ns*	*ns*	2.5
ABCC1	ATP binding cassette subfamily C member 1	*ns*	-2.5	*ns*
PARVA	parvin, alpha	*ns*	-2.5	*ns*
VWCE	von Willebrand factor C and EGF domains	*ns*	-2.3	*ns*
TNS1	tensin 1	*ns*	*ns*	2.4
WDR44	WD repeat domain 44	*ns*	-2.4	*ns*
GALC	galactosylceramidase	*ns*	-2.4	*ns*
ARL6	ADP-ribosylation factor like GTPase 6	*ns*	*ns*	2.4
CAVIN3	caveolae associated protein 3	*ns*	*ns*	2.4
PAPSS1	3-phosphoadenosine 5-phosphosulfate synthase 1	*ns*	-2.4	*ns*
JOSD2	Josephin domain containing 2	*ns*	-2.4	*ns*
ARHGAP20	Rho GTPase activating protein 20	*ns*	*ns*	2.4
TMEM219	transmembrane protein 219	*ns*	-2.4	*ns*
FAM8A1	family with sequence similarity 8, member A1	*ns*	-2.4	*ns*
PGM2L1	phosphoglucomutase 2-like 1	*ns*	-2.4	*ns*
CARD6	caspase recruitment domain family, member 6	*ns*	*ns*	2.4
ZNF699	zinc finger protein 699	*ns*	-2.4	*ns*
PCYOX1	prenylcysteine oxidase 1	*ns*	-2.4	*ns*
DNAJC10	DnaJ (Hsp40) homolog, subfamily C, member 10	*ns*	-2.4	*ns*
C2	complement component 2	*ns*	*ns*	2.4
ALPK2	alpha kinase 2	*ns*	-2.4	*ns*
CPEB2	cytoplasmic polyadenylation element binding protein 2	*ns*	-2.4	*ns*
IL17RA	interleukin 17 receptor A	*ns*	-2.4	*ns*
RGS4	regulator of G-protein signaling 4	*ns*	-2.4	*ns*
CTIF	CBP80	*ns*	-2.4	*ns*
NMNAT1	nicotinamide nucleotide adenylyltransferase 1	*ns*	*ns*	2.4
PREPL	prolyl endopeptidase-like	*ns*	*ns*	2.4
TPGS2	tubulin polyglutamylase complex subunit 2	*ns*	-2.4	*ns*
TNS2	tensin 2	*ns*	*ns*	2.4
ZBTB8A	zinc finger and BTB domain containing 8A	*ns*	-2.4	*ns*
TXNDC15	thioredoxin domain containing 15	*ns*	-2.4	*ns*
CTSO	cathepsin O	*ns*	*ns*	2.4
RGS5	regulator of G-protein signaling 5	*ns*	*ns*	2.4
UBE2D1	ubiquitin conjugating enzyme E2D 1	*ns*	-2.4	*ns*
PLCD3	phospholipase C, delta 3	*ns*	-2.4	*ns*
SELENOW	selenoprotein W	*ns*	-2.4	*ns*
UST	uronyl-2-sulfotransferase	*ns*	*ns*	2.4
ACTR1B	ARP1 actin-related protein 1 homolog B, centractin beta (yeast)	*ns*	-2.4	*ns*

Development • Supplementary information

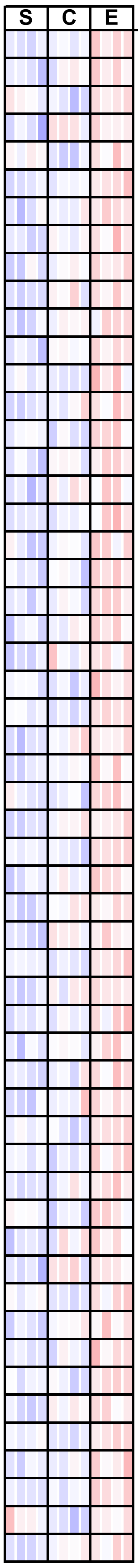

Symbol	Name	Linear Fold Change (*ns*=no significant difference) S vs C	S vs E	E vs C
LRRN4CL	LRRN4 C-terminal like	*ns*	-2.4	*ns*
OTUD1	OTU deubiquitinase 1	*ns*	-2.3	*ns*
LIG4	ligase IV, DNA, ATP-dependent	*ns*	*ns*	2.3
GLS	glutaminase	*ns*	-2.3	*ns*
THRA	thyroid hormone receptor, alpha	*ns*	*ns*	2.3
HLA-F	major histocompatibility complex, class I, F	*ns*	-2.3	*ns*
LOC100287042	uncharacterized LOC100287042	*ns*	-2.3	*ns*
IFI35	interferon-induced protein 35	*ns*	-2.3	*ns*
SLC39A6	solute carrier family 39 (zinc transporter), member 6	*ns*	-2.3	*ns*
SLC66A3	solute carrier family 66 member 3	*ns*	-2.3	*ns*
VKORC1	vitamin K epoxide reductase complex subunit 1	*ns*	-2.3	*ns*
LNPK	lunapark, ER junction formation factor	*ns*	-2.3	*ns*
LRRC6	leucine rich repeat containing 6	*ns*	*ns*	2.3
MX2	MX dynamin-like GTPase 2	*ns*	-2.3	*ns*
GYPC	glycophorin C (Gerbich blood group)	*ns*	*ns*	2.3
SS18L2	synovial sarcoma translocation gene on chromosome 18-like 2	*ns*	-2.3	*ns*
ODF3B	outer dense fiber of sperm tails 3B	*ns*	-2.3	*ns*
CA11	carbonic anhydrase XI	*ns*	-2.3	*ns*
LOC100996842	uncharacterized LOC100996842	*ns*	-2.3	*ns*
WASHC3	WASH complex subunit 3	*ns*	-2.3	*ns*
LOC102724094	uncharacterized LOC102724094	*ns*	-2.3	*ns*
OAF	out at first homolog	*ns*	-2.3	*ns*
FTH1	ferritin, heavy polypeptide 1	*ns*	-2.3	*ns*
TTC39B	tetratricopeptide repeat domain 39B	*ns*	*ns*	2.3
SLC9B2	solute carrier family 9, subfamily B (NHA2, cation proton antiporter 2), member 2	*ns*	*ns*	2.3
P4HA2	prolyl 4-hydroxylase, alpha polypeptide II	*ns*	-2.3	*ns*
INHBB	inhibin beta B	*ns*	-2.3	*ns*
ACAT1	acetyl-CoA acetyltransferase 1	*ns*	*ns*	2.3
ATP6V0E1	ATPase, H+ transporting, lysosomal 9kDa, V0 subunit e1	*ns*	-2.3	*ns*
TMEM204	transmembrane protein 204	*ns*	*ns*	2.3
RAMP1	receptor (G protein-coupled) activity modifying protein 1	*ns*	-2.3	*ns*
RGS16	regulator of G-protein signaling 16	*ns*	-2.3	*ns*
ZNF562	zinc finger protein 562	*ns*	-2.3	*ns*
DDAH1	dimethylarginine dimethylaminohydrolase 1	*ns*	*ns*	2.3
MIR3622A	microRNA 3622a	*ns*	-2.3	*ns*
LY86	lymphocyte antigen 86	*ns*	-2.3	*ns*
POLR2I	polymerase (RNA) II (DNA directed) polypeptide I, 14.5kDa	*ns*	-2.3	*ns*
S100A4	S100 calcium binding protein A4	*ns*	-2.3	*ns*
ERGIC1	endoplasmic reticulum-golgi intermediate compartment 1	*ns*	-2.3	*ns*
RUNX3	runt-related transcription factor 3	*ns*	*ns*	2.3
ZNF330	zinc finger protein 330	*ns*	*ns*	2.3
PSMB9	proteasome subunit beta 9	*ns*	-2.3	*ns*
KIR2DS4	killer cell immunoglobulin-like receptor, two domains, short cytoplasmic tail, 4	*ns*	*ns*	2.3
SOCS3	suppressor of cytokine signaling 3	*ns*	-2.2	*ns*
C1GALT1C1	C1GALT1 specific chaperone 1	*ns*	-2.2	*ns*
DECR1	2,4-dienoyl-CoA reductase 1, mitochondrial	*ns*	*ns*	2.2
TPRG1L	tumor protein p63 regulated 1-like	*ns*	-2.2	*ns*
MAP1A	microtubule associated protein 1A	*ns*	-2.2	*ns*
TCTA	T-cell leukemia translocation altered	*ns*	*ns*	2.2
DNASE2	deoxyribonuclease II, lysosomal	*ns*	-2.2	*ns*
ARL2BP	ADP-ribosylation factor like GTPase 2 binding protein	*ns*	*ns*	2.2
CELF2-AS1	CELF2 antisense RNA 1	*ns*	-2.2	*ns*
CYTIP	cytohesin 1 interacting protein	*ns*	-2.2	*ns*
HHEX	hematopoietically expressed homeobox	*ns*	*ns*	2.2
THBS3	thrombospondin 3	*ns*	-2.2	*ns*

Development • Supplementary information

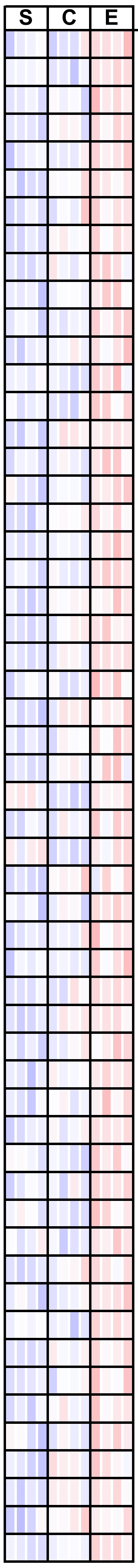

Symbol	Name	Linear Fold Change (*ns*=no significant difference) S vs C	S vs E	E vs C
TEX2	testis expressed 2	*ns*	-2.2	*ns*
RNF130	ring finger protein 130	*ns*	*ns*	2.2
FAM20A	family with sequence similarity 20, member A	*ns*	-2.2	*ns*
POT1-AS1	POT1 antisense RNA 1	*ns*	-2.2	*ns*
STAT3	signal transducer and activator of transcription 3 (acute-phase response factor)	*ns*	-2.2	*ns*
SIAE	sialic acid acetylesterase	*ns*	-2.2	*ns*
AHNAK2	AHNAK nucleoprotein 2	*ns*	-2.2	*ns*
KLHL2	kelch-like family member 2	*ns*	-2.2	*ns*
ZYX	zyxin	*ns*	-2.2	*ns*
ZNHIT1	zinc finger, HIT-type containing 1	*ns*	-2.2	*ns*
LTA4H	leukotriene A4 hydrolase	*ns*	-2.2	*ns*
SLC37A3	solute carrier family 37, member 3	*ns*	-2.2	*ns*
TNXA	tenascin XA (pseudogene)	*ns*	*ns*	2.2
GGT3P	gamma-glutamyltransferase 3 pseudogene	*ns*	*ns*	2.2
ETHE1	ethylmalonic encephalopathy 1	*ns*	-2.2	*ns*
TWIST2	twist family bHLH transcription factor 2	*ns*	-2.2	*ns*
TUSC2	tumor suppressor candidate 2	*ns*	-2.2	*ns*
LITAF	lipopolysaccharide-induced TNF factor	*ns*	-2.2	*ns*
FKBP1B	FK506 binding protein 1B	*ns*	-2.2	*ns*
AKAP12	A kinase (PRKA) anchor protein 12	*ns*	-2.2	*ns*
LY6D	lymphocyte antigen 6 complex, locus D	*ns*	-2.2	*ns*
LHFPL6	LHFPL tetraspan subfamily member 6	*ns*	-2.2	*ns*
ASL	argininosuccinate lyase	*ns*	-2.2	*ns*
KREMEN1	kringle containing transmembrane protein 1	*ns*	-2.2	*ns*
PROCR	protein C receptor, endothelial	*ns*	-2.2	*ns*
MARCHF2	membrane associated ring-CH-type finger 2	*ns*	-2.2	*ns*
TWF2	twinfilin actin binding protein 2	*ns*	-2.2	*ns*
ADD3	adducin 3 (gamma)	*ns*	*ns*	2.2
ZBTB16	zinc finger and BTB domain containing 16	*ns*	-2.2	*ns*
RAB31	RAB31, member RAS oncogene family	*ns*	*ns*	2.2
IL17RE	interleukin 17 receptor E	*ns*	-2.1	*ns*
FUNDC1	FUN14 domain containing 1	*ns*	-2.2	*ns*
H6PD	hexose-6-phosphate dehydrogenase (glucose 1-dehydrogenase)	*ns*	-2.2	*ns*
SPOCK2	sparc	*ns*	-2.2	*ns*
CLSTN2	calsyntenin 2	*ns*	-2.2	*ns*
LGALS3	lectin, galactoside-binding, soluble, 3	*ns*	-2.2	*ns*
MED11	mediator complex subunit 11	*ns*	-2.2	*ns*
ABR	active BCR-related	*ns*	-2.2	*ns*
GNA15	guanine nucleotide binding protein (G protein), alpha 15 (Gq class)	*ns*	-2.1	*ns*
ITGA3	integrin alpha 3	*ns*	-2.1	*ns*
CASP1	caspase 1	*ns*	*ns*	2.1
MR1	major histocompatibility complex, class I-related	*ns*	-2.1	*ns*
STX2	syntaxin 2	*ns*	*ns*	2.1
GPR4	G protein-coupled receptor 4	*ns*	*ns*	2.1
ABCA1	ATP binding cassette subfamily A member 1	*ns*	-2.1	*ns*
CYP2U1	cytochrome P450, family 2, subfamily U, polypeptide 1	*ns*	-2.1	*ns*
CYTH4	cytohesin 4	*ns*	*ns*	2.1
FKBP10	FK506 binding protein 10	*ns*	-2.1	*ns*
LRRC37A3	leucine rich repeat containing 37, member A3	*ns*	-2.1	*ns*
SLCO3A1	solute carrier organic anion transporter family, member 3A1	*ns*	-2.1	*ns*
TRG-AS1	T cell receptor gamma locus antisense RNA 1	*ns*	-2.1	*ns*
ODF2L	outer dense fiber of sperm tails 2-like	*ns*	-2.1	*ns*
LMAN2	lectin, mannose-binding 2	*ns*	-2.1	*ns*
IRF9	interferon regulatory factor 9	*ns*	-2.0	*ns*
CAPN5	calpain 5	*ns*	-2.1	*ns*

Development • Supplementary information

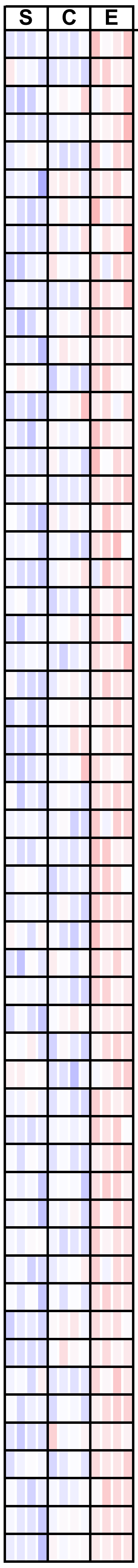

Symbol	Name	Linear Fold Change (*ns*=no significant difference) S vs C	S vs E	E vs C
NFASC	neurofascin	*ns*	-2.1	*ns*
METTL24	methyltransferase like 24	*ns*	*ns*	2.1
SNHG16	small nucleolar RNA host gene 16	*ns*	-2.1	*ns*
PPP1R7	protein phosphatase 1, regulatory subunit 7	*ns*	-2.1	*ns*
MFSD6	major facilitator superfamily domain containing 6	*ns*	*ns*	2.1
METTL23	methyltransferase like 23	*ns*	-2.1	*ns*
HOXA5	homeobox A5	*ns*	-2.1	*ns*
SLC31A1	solute carrier family 31 (copper transporter), member 1	*ns*	-2.1	*ns*
TNFRSF21	tumor necrosis factor receptor superfamily, member 21	*ns*	-2.1	*ns*
SPIRE1	spire-type actin nucleation factor 1	*ns*	-2.1	*ns*
HLA-DQB2	major histocompatibility complex, class II, DQ beta 2	*ns*	-2.1	*ns*
BLOC1S6	biogenesis of lysosomal organelles complex-1, subunit 6, pallidin	*ns*	-2.1	*ns*
CNTN4	contactin 4	*ns*	*ns*	2.1
CARS1	cysteinyl-tRNA synthetase 1	*ns*	-2.1	*ns*
GPBAR1	G protein-coupled bile acid receptor 1	*ns*	-2.1	*ns*
LOC100506928	uncharacterized LOC100506928	*ns*	-2.1	*ns*
SLA	Src-like-adaptor	*ns*	*ns*	2.1
FAM98C	family with sequence similarity 98, member C	*ns*	-2.1	*ns*
BBS5	Bardet-Biedl syndrome 5	*ns*	*ns*	2.1
FZD8	frizzled class receptor 8	*ns*	-2.1	*ns*
SMAP2	small ArfGAP2	*ns*	*ns*	2.1
CYB5R3	cytochrome b5 reductase 3	*ns*	-2.1	*ns*
DST	dystonin	*ns*	*ns*	2.1
OXLD1	oxidoreductase-like domain containing 1	*ns*	-2.1	*ns*
LEPROTL1	leptin receptor overlapping transcript-like 1	*ns*	-2.1	*ns*
TAP2	transporter 2, ATP-binding cassette, sub-family B (MDR	*ns*	-2.1	*ns*
IGF2R	insulin-like growth factor 2 receptor	*ns*	-2.1	*ns*
POLR2L	polymerase (RNA) II (DNA directed) polypeptide L, 7.6kDa	*ns*	-2.1	*ns*
ZNF189	zinc finger protein 189	*ns*	*ns*	2.1
MASP1	mannan-binding lectin serine peptidase 1 (C4	*ns*	-2.1	*ns*
CDK14	cyclin-dependent kinase 14	*ns*	*ns*	2.1
RASGRP2	RAS guanyl releasing protein 2 (calcium and DAG-regulated)	*ns*	*ns*	2.1
KIR3DL1	killer cell immunoglobulin-like receptor, three domains, long cytoplasmic tail, 1	*ns*	*ns*	2.1
FITM2	fat storage-inducing transmembrane protein 2	*ns*	-2.1	*ns*
RNLS	renalase, FAD-dependent amine oxidase	*ns*	*ns*	2.1
B2M	beta-2-microglobulin	*ns*	-2.1	*ns*
ACYP2	acylphosphatase 2, muscle type	*ns*	*ns*	2.1
RRN3P2	RRN3 homolog, RNA polymerase I transcription factor pseudogene 2	*ns*	*ns*	2.1
SLCO2B1	solute carrier organic anion transporter family, member 2B1	*ns*	*ns*	2.1
CHST3	carbohydrate (chondroitin 6) sulfotransferase 3	*ns*	-2.1	*ns*
LYSMD2	LysM, putative peptidoglycan-binding, domain containing 2	*ns*	*ns*	2.0
NBN	nibrin	*ns*	-2.0	*ns*
ME2	malic enzyme 2, NAD(+)-dependent, mitochondrial	*ns*	-2.0	*ns*
ACE	angiotensin I converting enzyme	*ns*	*ns*	2.0
ABHD2	abhydrolase domain containing 2	*ns*	-2.0	*ns*
ENDOD1	endonuclease domain containing 1	*ns*	-2.0	*ns*
MPC1	mitochondrial pyruvate carrier 1	*ns*	-2.0	*ns*
FKBP11	FK506 binding protein 11	*ns*	-2.0	*ns*
SASH3	SAM and SH3 domain containing 3	*ns*	*ns*	2.0
RNASEH2C	ribonuclease H2, subunit C	*ns*	*ns*	2.0
PDCL3	phosducin like 3	*ns*	-2.0	*ns*
RDH10	retinol dehydrogenase 10 (all-trans)	*ns*	*ns*	2.0
RAB29	RAB29, member RAS oncogene family	*ns*	-2.0	*ns*
ATL3	atlastin GTPase 3	*ns*	-2.0	*ns*
EIF2AK3	eukaryotic translation initiation factor 2-alpha kinase 3	*ns*	-2.0	*ns*

Development • Supplementary information

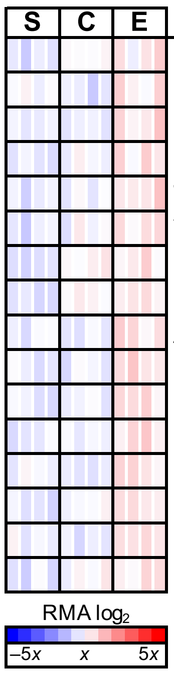

Symbol	Name	Linear Fold Change (*ns*=no significant difference) S vs C	S vs E	E vs C
LOC105376958	uncharacterized LOC105376958	*ns*	-2.0	*ns*
RASSF4	Ras association (RalGDS	*ns*	*ns*	2.0
ST6GAL1	ST6 beta-galactosamide alpha-2,6-sialyltranferase 1	*ns*	*ns*	2.0
SAP30	Sin3A associated protein 30kDa	*ns*	-2.0	*ns*
THYN1	thymocyte nuclear protein 1	*ns*	-2.0	*ns*
YBEY	ybeY metallopeptidase (putative)	*ns*	-2.0	*ns*
CD151	CD151 molecule (Raph blood group)	*ns*	-2.0	*ns*
DSTN	destrin (actin depolymerizing factor)	*ns*	-2.0	*ns*
ADGRG3	adhesion G protein-coupled receptor G3	*ns*	*ns*	2.0
PNMA1	paraneoplastic Ma antigen 1	*ns*	-2.0	*ns*
CFAP36	cilia and flagella associated protein 36	*ns*	-2.0	*ns*
SAPCD1-AS1	SAPCD1 antisense RNA 1	*ns*	-2.0	*ns*
PRICKLE2-AS1	PRICKLE2 antisense RNA 1	*ns*	*ns*	2.0
SDC3	syndecan 3	*ns*	-2.0	*ns*
GOLM1	golgi membrane protein 1	*ns*	*ns*	2.0
PDE4A	phosphodiesterase 4A, cAMP-specific	*ns*	-2.0	*ns*

Fig. S2. Syncytiotrophoblast (**S**), Cytotrophoblast (**C**), and Endovascular trophoblast (**E**) differentially expressed genes

Development • Supplementary information

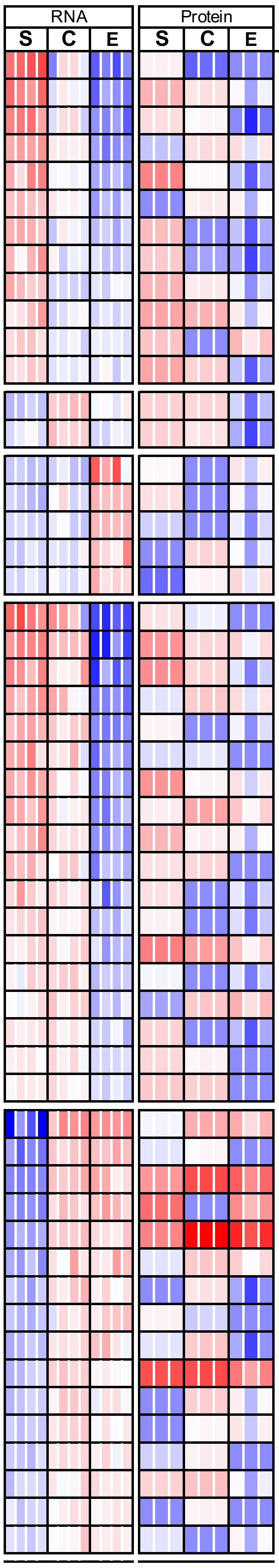

Symbol	Name	RNA SvC	RNA SvE	RNA EvC	Protein SvC	Protein SvE	Protein EvC
GH2	growth hormone 2	12	65	-6	7	42	-6
CSHL1	chorionic somatomammotropin hormone-like 1	7	5	-5	3	7	-3
KMO	kynurenine 3-monooxygenase	5	5	-5	2	10	-6
GPC3	glypican 3	3	4	-6	-5	-4	-1
HSD11B2	hydroxysteroid (11-beta) dehydrogenase 2	4	3	-3	19	58	-3
PSG3	pregnancy specific beta-1-glycoprotein 3	2	3	-3	-54	-32	-2
C4orf36	chromosome 4 open reading frame 36	4	3	-2	159	13	12
CKMT1A	creatine kinase, mitochondrial 1A	3	2	-1	10	12	-1
CA8	carbonic anhydrase VIII	4	2	1	4	9	-3
FURIN	furin (paired basic amino acid cleaving enzyme)	3	2	-1	1	8	-6
AK3	adenylate kinase 3	3	1	1	135	-1	148
ELK1	ELK1, member of ETS oncogene family	2	1	-1	3	23	-9
IL1RAP	interleukin 1 receptor accessory protein	-5	1	-2	-1	7	-7
NOCT	nocturnin	-3	0	-3	1	10	-7
GPR82	G protein-coupled receptor 82	-1	3	8	35	-1	44
RBP1	retinol binding protein 1, cellular	-2	3	3	64	2	28
C1QB	complement component 1, q subcomponent, B chain	-1	2	4	18	-1	21
CXCL11	chemokine (C-X-C motif) ligand 11	-1	2	3	-99	-25	-4
FBLN2	fibulin 2	-1	2	2	-7	-11	1
BMP5	bone morphogenetic protein 5	4	6	-21	2	62	-28
CYP19A1	cytochrome P450, family 19, subfamily A, polypeptide 1	2	6	-29	5	6	-1
HSD3B1	isomerase 1	2	5	-13	6	30	-5
DLK1	delta-like 1 homolog (Drosophila)	2	4	-10	-6	-3	-2
CDO1	cysteine dioxygenase type 1	2	4	-10	40	2	22
OLAH	oleoyl-ACP hydrolase	2	4	-6	-1	21	-26
STS	steroid sulfatase (microsomal), isozyme S	2	4	-6	11	10	1
ADAMTS6	ADAM metallopeptidase with thrombospondin type S6	2	4	-5	-7	-2	-3
ATP6V1C2	ATPase, H+ transporting, lysosomal 42kDa, V1C2	2	3	-6	4	6	-2
PSG1	pregnancy specific beta-1-glycoprotein 1	2	3	-4	-2	64	-100
SPDYA	speedy	1	3	-5	64	3	19
SLC38A1	solute carrier family 38, member 1	1	2	-3	42	2	17
SLC2A1	solute carrier family 2A1 (facilitated glucose transporter)	-1	2	-4	2	7	-4
CLN3	ceroid-lipofuscinosis, neuronal 3	-1	1	-4	28	2	18
SLC29A1	solute carrier family 29A1 (equilibrative nucleoside transporter)	-2	1	-4	-13	-17	1
UBE2C	ubiquitin-conjugating enzyme E2C	1	1	-2	93	8	12
ADK	adenosine kinase	-1	1	-2	2	82	-45
ZNF808	zinc finger protein 808	1	1	-2	-1	118	-119
PRG2	proteoglycan 2, bone marrow (natural killer cell activator)	-66	6	1	-10	-7	-1
ISG15	ISG15 ubiquitin-like modifier	-12	4	1	-2	23	-40
HSPG2	heparan sulfate proteoglycan 2	-12	3	-1	-9	-4	-2
RFPL4B	ret finger protein-like 4B	-8	3	1	1241	3	475
FN1	fibronectin 1	-7	2	-1	-35	-11	-3
IFIT1	interferon-induced protein with tetratricopeptide repeats 1	-5	3	1	-6	-4	-2
CD276	CD276 molecule	-5	2	-1	-70	-9	-8
LAYN	layilin	-3	2	2	2	40	-21
ST6GALNAC2	ST6 N-Acetylgalactosaminide Alpha-2,6-Sialyltransferase 2	-4	2	1	-6	2	-15
KRT19	keratin 19, type I	-4	1	-2	-1	4	-5
LY6K	lymphocyte antigen 6 complex, locus K	-4	1	-2	-116	-36	-3
FHL2	four and a half LIM domains 2	-3	1	-1	-40	-44	1
FAM110A	family with sequence similarity 110, member A	-3	1	-1	-3	18	-52
GTPBP1	GTP binding protein 1	-2	1	1	-2	3	-6
ADAM15	ADAM metallopeptidase domain 15	-2	1	-1	-6	8	-47
LASP1	LIM and SH3 protein 1	-2	1	1	23	-1	31

(Linear Fold Change columns: RNA SvC, SvE, EvC; Protein SvC, SvE, EvC.)

Development: doi:10.1242/dev.199626: Supplementary information

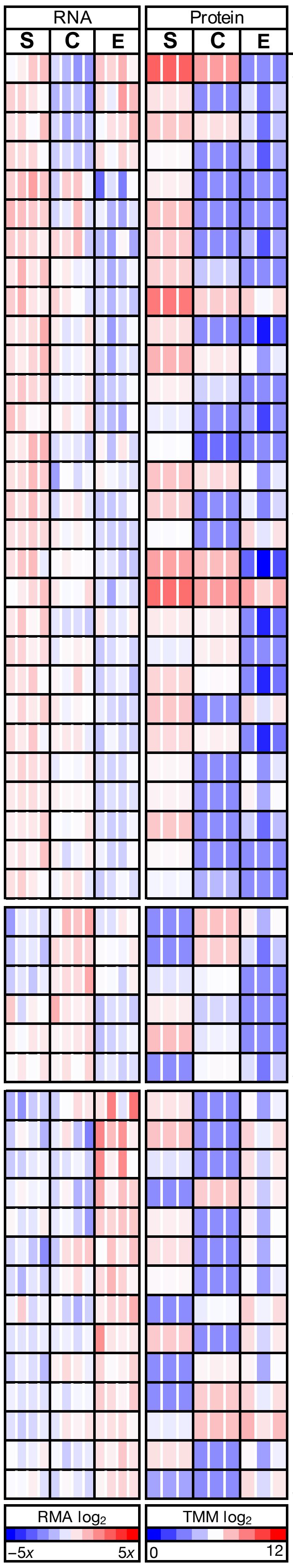

Symbol	Name	Linear Fold Change RNA SvC	RNA SvE	RNA EvC	Protein SvC	Protein SvE	Protein EvC
ACTA2	actin, alpha 2, smooth muscle, aorta	4	0	5	4	1763	-397
IDO1	indoleamine 2,3-dioxygenase 1	4	0	5	59	3	17
TCF4	transcription factor 4	4	0	4	2	7	-4
EAF2	ELL associated factor 2	3	0	3	35	3	12
PSG11	pregnancy specific beta-1-glycoprotein 11	2	3	-3	5	45	-9
GH1	growth hormone 1	2	2	-2	138	138	-1
PBK	PDZ binding kinase	1	2	-3	113	10	11
RBMS3	RNA binding motif, single stranded interacting protein 3	2	2	-2	5	94	-20
TFRC	transferrin receptor	2	2	-2	8	12	-1
NPIPB4	nuclear pore complex interacting protein family B4	2	2	-2	66	11	6
DVL3	dishevelled segment polarity protein 3	2	2	-2	4	8	-2
STX3	syntaxin 3	2	2	-2	2	45	-22
PDXDC2P	pyridoxal-dependent decarboxylase domain containing 2, pseudogene	1	2	-2	25	3	9
BTG2	BTG family, member 2	3	1	1	5	30	-6
BCL2	B-cell CLL	3	1	1	2	5	-3
AUNIP	aurora kinase A and ninein interacting protein	2	2	-2	10	5	2
TOMM34	translocase of outer mitochondrial membrane 34	1	2	-2	30	-2	63
RGPD3	RANBP2-like and GRIP domain containing 3	1	1	-2	2	67	-38
H2BC21	H2B clustered histone 21	1	1	-2	3	5	-2
SLAIN1	SLAIN motif family member 1	3	1	2	-1	7	-8
SLC2A11	solute carrier family 2A11 (facilitated glucose transporter)	1	1	-2	-2	26	-54
TAF4B	TATA-Box Binding Protein Associated Factor 4b	1	1	-2	2	13	-6
MGST3	microsomal glutathione S-transferase 3	1	1	-2	12	2	6
TIGD2	tigger transposable element derived 2	1	1	-2	2	11	-7
SIAH2	siah E3 ubiquitin protein ligase 2	2	1	-1	37	2	24
TLK1	tousled-like kinase 1	1	1	-2	40	1	31
HNRNPD	heterogeneous nuclear ribonucleoprotein D	1	1	-2	118	8	15
NPIPA5	nuclear pore complex interacting protein family A5	2	1	-1	35	35	-1
TCTEX1D4	Tctex1 domain containing 4	1	1	-2	2	28	-15
PGPEP1	pyroglutamyl-peptidase I	-4	1	-2	-150	-34	-4
PANX1	pannexin 1	-3	1	-2	-108	-17	-7
ELF4	E74-like factor 4 (ets domain transcription factor)	-3	1	-2	-1	23	-34
RAB25	RAB25, member RAS oncogene family	-1	1	-3	2	47	-21
CYB5R1	cytochrome b5 reductase 1	-1	1	-2	5	152	-29
ACVR2B	activin A receptor type IIB	-1	1	-2	-36	-17	-2
TM4SF5	transmembrane 4 L six family member 5	-3	3	2	57	2	27
GPR65	G protein-coupled receptor 65	1	2	5	133	2	68
CD82	CD82 molecule	-1	2	4	23	-2	50
PLEK	pleckstrin	2	1	4	-129	-45	-3
GDAP1	ganglioside induced differentiation associated protein 1	2	1	4	45	1	33
SLC30A4	solute carrier family 30 (zinc transporter), member 4	-3	2	1	64	2	31
MGAT2	mannosyl (a-1,6-)-glycoprotein b-1,2-N-acetylglucosaminyltransferase	-2	2	2	35	1	26
BTN3A2	butyrophilin, subfamily 3, member A2	2	1	3	-31	-74	2
C4B	complement component 4B (Chido blood group)	-1	1	2	112	2	52
CPEB2	cytoplasmic polyadenylation element binding protein 2	-2	1	1	-46	-31	-1
CYTIP	cytohesin 1 interacting protein	-1	1	2	-126	-68	-2
LGALS3	lectin, galactoside-binding, soluble, 3	-2	1	1	-6	-6	1
GPR4	G protein-coupled receptor 4	1	1	2	62	2	27
ACYP2	acylphosphatase 2, muscle type	1	1	2	11	-5	55

Fig. S3. Syncytiotrophoblast (**S**), Cytotrophoblast (**C**), and Endovascular trophoblast (**E**) coordinately differentially expressed genes and proteins

Development • Supplementary information

Table S1. Antibodies

Antigen	Catalog number/ clone	Source	Species	Concentration (µg/ml)
NTS	ab172114	Abcam	Rabbit	10
C4ORF36	ab23703	Abcam	Rabbit	2
PGPEP1	ab220735	Abcam	Rabbit	0.5
CNR1	ab23703	Abcam	Rabbit	4
CK7	7D3	Damsky et al., 1992	Rat	26
FITC-conjugated anti-mouse secondary	715-095-151	Jackson ImmunoResearch	Donkey	15
FITC-conjugated anti-rabbit secondary	711-095-152	Jackson ImmunoResearch	Donkey	15
TRITC-conjugated anti-rat secondary	712-025-153	Jackson ImmunoResearch	Donkey	15

Table S2. Cannabinoid Receptor Agonists and Antagonists

Compound	CAS Registry No	Tocris Cat. No.	Target
(R)-(+)-Methanandamide	157182-49-5	1782	CB_1 Agonist
AM 251	183232-66-8	1117	CB_1 Receptor Antagonist
JWH 133	259869-55-1	1343	CB_2 Agonist
AM 630	164178-33-0	1120	CB_2 Inverse Agonist

Development • Supplementary information
