# Supplementary material for: RNA profiling of laser microdissected human trophoblast subtypes at mid-gestation reveals a role for cannabinoid signaling in invasion
Source: Development. 2021 Oct 19;148(20):dev199626. doi: 10.1242/dev.199626 (PMC8572005; doi:10.1242/dev.199626)
Supplement: Reviewer comments [file develop-148-199626_review_history.pdf]

RNA profiling of laser microdissected human trophoblast subtypes at mid-gestation reveals a role for cannabinoid signaling in invasion

Matthew Gormley, Oliver Oliverio, Mirhan Kapidzic, Katherine Ona, Steven Hall and Susan Fisher

DOI: 10.1242/dev.199626

Editor: Liz Robertson

Review timeline

Original submission:	8 April 2021
Editorial decision:	17 May 2021
First revision received:	21 July 2021
Accepted:	26 August 2021

Original submission

First decision letter

MS ID#: DEVELOP/2021/199626

MS TITLE: RNA Profiling of Laser Microdissected Human Trophoblast Subtypes at Mid-Gestation Reveals a Role for Cannabinoid Signaling in Invasion

AUTHORS: Matthew Gormley, Oliver Oliverio, Mirhan Kapidzic, Katherine Ona, Steven Hall, and Susan Fisher

I have now received all the referees' reports on the above manuscript, and have reached a decision. The referees' comments are appended below, or you can access them online: please go to BenchPress and click on the 'Manuscripts with Decisions' queue in the Author Area.

As you will see, the referees express considerable interest in your work, but have some significant criticisms and recommend a substantial revision of your manuscript before we can consider publication. If you are able to revise the manuscript along the lines suggested, which may involve further experiments, I will be happy receive a revised version of the manuscript. Your revised paper will be re-reviewed by one or more of the original referees, and acceptance of your manuscript will depend on your addressing satisfactorily the reviewers' major concerns. Please also note that Development will normally permit only one round of major revision.

We are aware that you may be experiencing disruption to the normal running of your lab that make experimental revisions challenging. If it would be helpful, we encourage you to contact us to discuss your revision in greater detail. Please send us a point-by-point response indicating where you are able to address concerns raised (either experimentally or by changes to the text) and where you will not be able to do so within the normal timeframe of a revision. We will then provide further guidance. Please also note that we are happy to extend revision timeframes as necessary.

Please attend to all of the reviewers' comments and ensure that you clearly highlight all changes made in the revised manuscript. Please avoid using 'Tracked changes' in Word files as these are lost in PDF conversion. I should be grateful if you would also provide a point-by-point response detailing how you have dealt with the points raised by the reviewers in the 'Response to Reviewers' box. If you do not agree with any of their criticisms or suggestions please explain clearly why this is so.

© 2021. Published by The Company of Biologists under the terms of the Creative Commons Attribution License (https://creativecommons.org/licenses/by/4.0/).

Reviewer 1

Advance summary and potential significance to field

This manuscript describes the generation of transcriptomic and proteomic profiles from specific second trimetser placental cell populations that were isolated by laser microdissection. The strengths of this study are the use of the same placental material to generate these two complimentary data sets, and the identification of some new factors and pathways that may play a role in the function of the respective cell type. Few immunostainings were performed to verify the cell type-specific localization of factors, such as the endocannabinoid receptor 1, inferring potential function in trophoblast invasion.

Comments for the author

As it stands, the study is a robust and valuable data resource, but provides little insight beyond that. There is no doubt that the RNA-seq and mass spectrometry profiles from laser-microdissected material of 4 individual placentas was a substantial amount of work. Immunostainings for four factors confirms the specificity of the data. But no functional evidence into the roles of any of the newly identified factors in these cell types is provided, that aspect remains at the level of speculation. As such, for the most part the manuscripts only details heatmaps, expression trajectories and pathway analyses.
A minor comment is that the section on p.5, bottom half, is unclear. How can the absence of expression of a gene in one cell type reveal its presence in another cell type? Shouldn't these genes come out of the DESeq analysis, just like those highlighted in the previous section?

Reviewer 2

Advance summary and potential significance to field

The authors provide new insight into transcriptomic and proteomic profiles of 2nd trimester human trophoblast cells.

The use of laser capture microdissection represents a strategy for preparing trophoblast cell samples from an early stage of human placentation for 'omic analysis not previously reported.

Through the transcriptomic and proteomic analysis the authors identify a cannabinoid signaling pathway that potentially regulates trophoblasts.

Comments for the author

This is an excellent report that adds to our knowledge of human trophoblast cell development. Some issues that should be considered.
1. Preparations for transcriptomic and proteomic analysis. The authors indicate that samples were obtained from tissue specimens reflecting four time points during the second trimester of pregnancy. The authors need to provide a clear description of the specimens that contributed to each sample. Was one sample from gestation age 15 weeks, another from 17 weeks, another from 18 weeks, and finally from twenty weeks. If so, the four samples were stratified based on gestational age. How many microdissections from each sample were pooled for analysis?

2. The contamination of the endovascular trophoblast preparation with decidual cells, NK cells, etc is an important acknowledgement. This is effective for transcripts previously assigned to a cell type but less apparent for transcripts not previously assigned to endovascular trophoblast or the other cell types.
Instead, would it be more appropriate for the authors to expand the "descriptor" of the samples, indicating that they reflect the behavior of endovascular trophoblast, decidual cells, NK cells, etc. It may be misleading to some readers of the report that the profiles uniquely reflect the behavior of endovascular trophoblast.

© 2021. Published by The Company of Biologists under the terms of the Creative Commons Attribution License (https://creativecommons.org/licenses/by/4.0/).

3. As described, the column cytotrophoblast samples would appear to exhibit some heterogeneity and include progenitor cells and cells progressing along an extravillous trophoblast differentiation pathway. Some comments about the expected heterogeneity of the samples would be helpful.

4. Did the validation result in consistent findings or was heterogeneity observed in the expression of the proteins examined among the villi of a given specimen?
Did expression vary from gestation ages?

5. Over the past few years, there have been significant efforts to perform single cell RNAseq analysis of extravillous trophoblast isolated from the maternal-fetal interface. It would be helpful for the authors to comment on their findings versus the prior single cell RNAseq analyses. Were there similarities or differences? What are the advantages of each research strategy?

6. The authors identified the potential for cannabinoid signaling in endovascular trophoblast. It is not entirely clear why invasion through Matrigel was the parameter assessed. Are endovascular trophoblast actively invading or are endovascular trophoblast responsible for other biological activities? Are cytotrophoblast enzymatically isolated from the 2nd trimester placenta an acceptable surrogate for endovascular trophoblast? The response observed in vitro to cannabinoid agonists is interesting, but does it provide any insights into the role of cannabinoid signaling in endovascular trophoblast?

<u>Reviewer 3</u>

Advance summary and potential significance to field

The Manuscript by Gormley et al used laser capture microdissection (LCM) to study three different trophoblast populations, namely Syncytiotrophoblast (STB), Column Cytotrophoblast (CTB, precursors of invasive extravillous trophoblast cells) and endovascular trophoblasts, in normal 2nd-trimester human placenta. The study mainly focused on profiling differential gene expressions in trophoblast subpopulations.
Authors showed that a subset of extravillous CTBs and endovascular trophoblasts express cannabinoid receptor 1 (CB1) and in vitro studies with isolated CTBs showed that exposure to a CB1 agonist promotes their invasion. The study is important as we have a poor understanding of gene expression patterns in trophoblast cells in a developing human placenta, especially at 2nd trimester as the tissue is hard to obtain. However, I have several concerns with the study as it seems incomplete and the following suggestions should improve the manuscript.

Comments for the author

(1) The field is more advanced now and single-cell genomics strategy is robust to identify gene expression patterns in a tissue at single-cell resolution. The LCM-microarray study has multiple limitations as processed frozen tissues were used for capturing. Also, trophoblast populations from 4 different placentae at different gestational age and thus it is a representation of mixed cell population. Also, the endovascular trophoblast population shows high expression of Prolactin and IGFBP1, which are highly expressed in endometrial cells.
Thus, most probably those signals are no specific to endovascular cells. As the author has easy access to 2nd trimester tissue they should perform single-cell RNA seq to complement the study and to obtain better understanding of gene expression in individual cell types.

(2) In figure 5 authors showed protein expressions of a few genes including Neurotensin and C4orf36. It is not clear why they chose only those genes, especially authors simply mentioned that they were interested in the expression of C4orf36 without expression additional justification. Although the protein expression patterns is of interest, the study and data at its present form seems out of context. If they want to establish the unique cell type expression patterns, Authors should include protein expression analyses of additional genes, which are specifically induced at the mRNA level in specific trophoblast subtypes. They should also show variations with distinct gestational age. If they only wants to focus on Neurotensin, it is important to show the expression pattern across gestation and measure whether pregnancy induces the protein level in maternal circulation (rather than speculating such a conclusion).

© 2021. Published by The Company of Biologists under the terms of the Creative Commons Attribution License (https://creativecommons.org/licenses/by/4.0/).

(3) In figure 6 authors studied isolated CTBs. The isolated CTBs come from both anchoring and floating villi and only a subpopulation represents extravillous CTB. Thus, studies on CB1 expression and function in that context is inconclusive. Also, from Fig. 6 A,B,C it is not clear whether or no CB1 is expressed in CTBs and STBs of floating villi. More detailed expression analyses including placental samples from 1st to 3rd trimester placentae should have been analyzed to establish the importance of CB1-mediated pathway.

(4) Also, an induction of invasion with a pharmacological agonist is not a robust data to definitively conclude importance of CB1 signaling. In this context, this reviewer suggest to include studies with recently established human trophoblast stem cells as models and use loss of function approach.

First revision

Author response to reviewers' comments

Response to Reviewers: We thank the reviewers for their thoughtful consideration and insightful comments. Incorporating their recommendations has significantly improved the quality of the original submission. As outlined below, we have revised the manuscript to address specific concerns, suggestions, or questions. New text in the revision is highlighted in yellow, and the location of these changes in the revised manuscript is indicated in parenthesis in our responses below.

Reviewer 1 comments.

Comment 1: But no functional evidence into the roles of any of the newly identified factors in these cell types is provided…

Response 1: We share the reviewer's desire to determine the functional role of the many new factors we identified. For clarity, we focused on one factor, cannabinoid receptor 1 (CNR1). Though well studied in the reproductive tissues of the mouse (Sun and Dey, 2012; PMID: 23223073), there are no equivalent studies in humans. In figure 6, we demonstrated the *in situ* localization of CNR1, quantified its endogenous agonists, and measured the effect of CNR1 and CNR2 agonists and antagonists on trophoblast invasion. These findings have translational importance. In the past 9 years alone, 17 states across the USA have legalized recreational cannabis (marijuana) use. The principal psychoactive ingredient in marijuana, tetrahydrocannabinol (THC), is also a CNR1 agonist. Our data provides a cautionary tale about cannabis intake during pregnancy, which the American College of Obstetricians and Gynecologists estimates to be 5% of woman.

Comment 2: …the section on p.5, bottom half, is unclear.

Response 2: We removed it.

Reviewer 2 comments.

Comment 1a: The authors need to provide a clear description of the specimens that contributed to each sample. Was one sample from gestation age 15 weeks, another from 17 weeks, another from 18 weeks, and finally from twenty weeks. If so, the four samples were stratified based on gestational age.

Response 1a: We added the text suggested by the reviewer, "Biopsies of the maternal-fetal interface were taken from four placentas with gestational ages stratified across the second trimester (15, 17, 18 and 20 weeks, respectively)." (PAGE 11).

Comment 1b: How many microdissections from each sample were pooled for analysis?

© 2021. Published by The Company of Biologists under the terms of the Creative Commons Attribution License (https://creativecommons.org/licenses/by/4.0/).

Response 1b: We have added clarifying text, "For RNA, 10 slides with 4 sections per slide were prepared for each specimen. For protein, 5 slides with 8 sections per slide were prepared. To limit degradation, each slide used for RNA isolation was microdissected for no more than 30 minutes, which was extended to 1 hour for protein recovery. The regions of interest were collected into different caps and pooled across all 40 tissue sections." (PAGE 12).

Comment 2: Would it be more appropriate for the authors to expand the "descriptor"…It may be misleading to some readers of the report that the profiles uniquely reflect the behavior of endovascular trophoblast.

Response 2: We modified our descriptor to "endovascular compartment (ENDO)" and expanded its definition throughout the manuscript to include "trophoblasts and other cell types." (PAGES 2, 3, 4, 5, 6, 7, 8, 9).

Comment 3: Some comments about the expected heterogeneity of the samples (column cytotrophoblast) would be helpful.

Response 3: We added text to the introduction as recommended by the reviewer. For context, the previous sentence is also included. "In the other pathway, the cells leave the placenta, forming bridges, termed cell columns, that connect to the uterus and are the conduit for CTBs that invade its wall. The process is accompanied by a dramatic phenotypic switch in which the formerly epithelial cells adopt many vascular properties (Damksy and Fisher 1998; PMID 9818178)." (Page 3).

Comment 4a: Did the validation result in consistent findings or was heterogeneity observed in the expression of the proteins examined among the villi of a given specimen?

Response 4a: We added additional text to the relevant figure to further describe the results, "Neurotensin (NTS) was detected in a vesicular pattern localized to the apical region of syncytiotrophoblasts (STB). Within any one tissue section the vesicle density varied among regions although no areas lacked immunoreactivity. Furthermore, no differences based on gestational age were observed. C4ORF36, which localized to STBs, had a uniformly dense punctate pattern. No variation was observed across the second trimester." (Figure 5 legend).

Comment 4b: Did expression vary from gestation ages?

Response 4b: Please see our response to the previous comment.

Comment 5: It would be helpful for the authors to comment on their findings versus the prior single cell RNAseq analyses. Were there similarities or differences? What are the advantages of each research strategy?

Response 5: To the best of our knowledge, only one paper has reported single-cell RNASeq data from human second trimester cytotrophoblasts (Liu et al 2018; PMID: 30042384). Comparisons of our RNA results with the published work showed a 91% overlap (2728 of 2986 transcripts). Unfortunately, they confirmed the trophoblast protein expression of only two genes, Ribonucleotide Reductase Regulatory Subunit M2 (RRM2) and Syncytin-2 (ERVFRD-1), the latter appearing among the peptides we detected using mass spectroscopy (however the spectral count profile did not reach statistical significance).

Comment 6a: It is not entirely clear why invasion through Matrigel was the parameter assessed. Are endovascular trophoblast actively invading or are endovascular trophoblast responsible for other biological activities?

Response 6a: Cytotrophoblast uterine penetration culminates in endovascular invasion. Similarly, Matrigel invasion enables differentiation of these specialized cells over 48 hours, the reason we use this assay to parse the functions of CNR1.

Comment 6b: Are cytotrophoblast enzymatically isolated from the 2nd trimester placenta an

© 2021. Published by The Company of Biologists under the terms of the Creative Commons Attribution License (https://creativecommons.org/licenses/by/4.0/).

acceptable surrogate for endovascular trophoblast?

Response 6b: Please see our response to the previous comment.

Comment 6c: The response observed in vitro to cannabinoid agonists is interesting, but does it provide any insights into the role of cannabinoid signaling in endovascular trophoblast?

Response 6c: Yes, we think our results provide new insights as our findings raise the possibility that cannabinoid signaling could alter trophoblast endovascular invasion, which in turn, is a major determinant of pregnancy outcomes.

Reviewer 3 comments.

Comment 1a: The field is more advanced now and single-cell genomics strategy is robust to identify gene expression patterns in a tissue at single-cell resolution. The LCM-microarray study has multiple limitations as processed frozen tissues were used for capturing.

Response 1a: Single-cell and LCM methods yield different types of data. Without further validation using other methods, the location of particular subpopulations is unknown and surmised from developmental trajectories that are revealed in the analysis of single-cell RNASeq data. In contrast, LCM enables capture of cells from geographically defined regions. In addition, the sample processing times are much shorter than those that required for enzymatic dissociation of tissue, which in and of itself could result in changes in gene expression. In addition, STBs from second trimester placentas are very difficult to profile using single-cell methods, as illustrated by the absence of these data in the Liu et al paper referenced above (Liu et al 2018; PMID: 30042384). Moreover, one of our major goals was to compare gene and protein expression, which is not yet possible using single-cell methods.

Comment 1b: Trophoblast populations from 4 different placentae at different gestational age and thus it is a representation of mixed cell population.

Response 1b: We previously demonstrated that there is no significant transcriptional variation within second trimester placental samples: gestational ages 14 – 24 weeks (Winn et al 2007; PMID: 17170095). Here, we focused on second trimester specimens 15 – 20 weeks that were encompassed by the gestational window that was the subject of our former paper.

Comment 1c: Also, the endovascular trophoblast population shows high expression of Prolactin and IGFBP1, which are highly expressed in endometrial cells. Thus, most probably those signals are no specific to endovascular cells.

Response 1c: One of the advantages of laser capture is visualization of the cell types that are being isolated. Since we were microdissecting the lumina of spiral arteries, we were able to exclude the possibility of significant decidual cell contamination. In addition, our previous work shows that extravillous cytotrophoblasts, as well as decidual cells, express prolactin and IGFBP1. However, as stated in the paper, we detected signals from NK cells that reside alongside CTBs in the arterial walls.

Comment 1d: As the author has easy access to 2nd trimester tissue they should perform single-cell RNA seq to complement the study and to obtain better understanding of gene expression in individual cell types.

Response 1d: As stated in our response to this reviewer's first comment, our goal was to use LCM to compare RNA and protein expression in the various second trimester trophoblast subpopulations analyzed. As noted above, STBs of this gestational age have not been successfully profiled using single-cell methods.

Comment 2a: In figure 5 authors showed protein expressions of a few genes including Neurotensin and C4orf36. It is not clear why they chose only those genes, especially authors simply mentioned that they were interested in the expression of C4orf36 without expression additional justification.

© 2021. Published by The Company of Biologists under the terms of the Creative Commons Attribution License (https://creativecommons.org/licenses/by/4.0/).

Response 2a: In the results section we state, "we chose neurotensin, a small peptide gut hormone, with brain and nervous system effects including analgesia (34-36) which to our knowledge was not known to be produced by the human placenta." And further, "where it seems likely that enteric and other effects are possible." Thus, neurotensin was chosen because its expression has not been documented in the human placenta and its presence may explain the common complication of hyperemesis during pregnancy. Concerning C4orf36, "We validated translation of an open reading frame (*CHROMOSOME 4 OPEN READING FRAME 36; C4ORF36*)." In the discussion we state, "we also confirmed that *C4ORF36* is translated." Confirming the transcription and translation of a hereto unnamed gene with unknown function, that previously was only theorized to be transcribed owing to possessing an open reading frame, is the justification.

Comment 2b: If they want to establish the unique cell type expression patterns, Authors should include protein expression analyses of additional genes, which are specifically induced at the mRNA level in specific trophoblast subtypes.

Response 2b: We note that we used a mass spectrometry approach to validate the mRNA findings. Specifically, we confirmed the expression patterns of 102 transcripts.

Comment 2c: They should also how variations with distinct gestational age.

Response 2c: Reviewer 2 also shared this concern. Please see the response to comment 4a.

Comment 2d: If they only wants to focus on Neurotensin, it is important to show the expression pattern across gestation and measure whether pregnancy induces the protein level in maternal circulation (rather than speculating such a conclusion).

Response 2d: In this study we focused on cannabinoid receptor 1 (CNR1). We agree with the reviewer that measuring neurotensin in maternal blood across gestation is important, but analyzing levels of this neuropeptide as function of gestational age is outside the scope of our current study.

Comment 3a: In figure 6 authors studied isolated CTBs. The isolated CTBs come from both anchoring and floating villi and only a subpopulation represents extravillous CTB. Thus, studies on CB1 expression and function in that context is inconclusive.

Response 3a: See response to Reviewer 2, comment 6a.

Comment 3b: Also, from Fig. 6 A,B,C it is not clear whether or no CB1 is expressed in CTBs and STBs of floating villi.

Response 3b: We added additional text to the relevant figure to further describe the results, "Cytotrophoblasts in the cell columns showed variable CB1 expression, while syncytiotrophobasts were negative (data not shown)." (Figure 6 legend).

Comment 3c: More detailed expression analyses including placental samples from 1st to 3rd trimester placentae should have been analyzed to establish the importance of CB1-mediated pathway.

Response 3c: Our goal was to verify CB1 expression during the gestational ages analyzed, rather than profile expression across pregnancy.

Comment 4a: Also, an induction of invasion with a pharmacological agonist is not a robust data to definitively conclude importance of CB1 signaling. In this context, this reviewer suggest to include studies with recently established human trophoblast stem cells as models and use loss of function approach.

Response 4a: The use of well characterized pharmacological agents has been an extremely powerful approach to understand CB1 biology (Wang et al 2004; PMID 15378054). The reviewer's

© 2021. Published by The Company of Biologists under the terms of the Creative Commons Attribution License (https://creativecommons.org/licenses/by/4.0/).

suggestion to use established human trophoblast stem cells as a model is interesting. Thus, we reviewed the RNASeq profiles of the cells in a relevant dataset (Okae et al 2018; PMID: 29249463). Unfortunately, *CNR1* mRNA is not detectable in their cells. Whether or not the receptor is upregulated as the cells differentiate remains to be determined.

Second decision letter

MS ID#: DEVELOP/2021/199626

MS TITLE: RNA Profiling of Laser Microdissected Human Trophoblast Subtypes at Mid-Gestation Reveals a Role for Cannabinoid Signaling in Invasion

AUTHORS: Matthew Gormley, Oliver Oliverio, Mirhan Kapidzic, Katherine Ona, Steven Hall, and Susan Fisher
ARTICLE TYPE: Research Article

I am happy to tell you that your manuscript has been accepted for publication in Development, pending our standard ethics checks.

Reviewer 1

Advance summary and potential significance to field

The authors have made a reasonable effort to address all reviewers' points within the scope of feasibility. I agree that additional scRNA-seq data would not achieve the same goal as the current study. The impact remains limited largely to data deposition, but having these data publicly accessible will be a great benefit to the field.

Comments for the author

N/A

Reviewer 2

Advance summary and potential significance to field

The authors have provided new insights into the phenotypes of trophoblast cells situated at the maternal-fetal interface.

Comments for the author

the authors have satisfactorily addressed my concerns

Reviewer 3

Advance summary and potential significance to field

The Manuscript focuses on differential gene and protein expression profiles in primary trophoblast cells of second trimester human placenta to identify novel signaling pathways that are involved in trophoblast invasion and endovascularization. Authors used laser capture microdissection (LCM) to study three different trophoblast populations, namely Syncytiotrophoblast (STB), Column Cytotrophoblast (CTB, precursors of invasive extravillous trophoblast cells) and endovascular trophoblasts, in normal 2nd-trimester human placenta. Authors showed that a subset of extravillous

© 2021. Published by The Company of Biologists under the terms of the Creative Commons Attribution License (https://creativecommons.org/licenses/by/4.0/).

CTBs and endovascular trophoblasts express cannabinoid receptor 1 (CB1) and in vitro studies with isolated CTBs showed that exposure to a CB1 agonist promotes their invasion. The study is important as we have a poor understanding of gene expression patterns in trophoblast cells in a developing human placenta, especially at 2nd trimester as the tissue is hard to obtain.

Comments for the author

The rebuttal letter and revised manuscript address most of my concerns. I do not have additional comments or suggestions.

© 2021. Published by The Company of Biologists under the terms of the Creative Commons Attribution License (https://creativecommons.org/licenses/by/4.0/).
